# Enhanced therapeutic potential of paeoniflorin and vitamin B12 in intracerebropeduncle ethidium bromide-induced multiple sclerosis-like pathology

**DOI:** 10.3389/fphar.2026.1792674

**Published:** 2026-04-10

**Authors:** MD Nasiruddin Khan, Sidharth Mehan, Divya Choudhary, Ravi Rana, Ghanshyam Das Gupta, Acharan S. Narula

**Affiliations:** 1 Division of Neuroscience, Department of Pharmacology, ISF College of Pharmacy, Moga, Punjab, India (Affiliated to IK Gujral Punjab Technical University), Jalandhar, Punjab, India; 2 Department of Pharmaceutics, ISF College of Pharmacy, Moga, Punjab, India (Affiliated to IK Gujral Punjab Technical University), Jalandhar, Punjab, India; 3 Narula Research, LLC, Chapel Hill, NC, United States

**Keywords:** Akt, demyelination, ERK1/2, ethidium bromide (EBRO), gdnf, Gfra1, GSK3-beta, multiple sclerosis (MS)

## Abstract

The study investigates the neuroprotective potential of paeoniflorin (PNN) in mitigating the multifaceted pathology of multiple sclerosis (MS) in an ethidium bromide-induced (EBRO) rat model. A comprehensive approach utilizing *in silico*, *in-vitro*, and *in-vivo* methodologies reveals that PNN targets key molecular pathways implicated in MS, including the GDNF/GFRA1/RET/AKT/ERK1/2/GSK3-Beta signaling cascade. PNN (50 mg/kg, 100 mg/kg, *p.o*.) administration, both as monotherapy and in combination with VB-12 (30 mg/kg, *p.o*.), demonstrated significant efficacy in reducing EBRO-induced neurodegeneration, demyelination, synaptic dysfunction, and neuroinflammation. Behavioral assessments such as the rotarod, beam crossing, and Morris water maze tests highlighted PNN capacity to restore motor coordination, spatial memory, and cognitive function. Combination therapy with VB12 (30) further enhanced these outcomes, demonstrating synergistic therapeutic benefits. Histological and molecular analyses revealed that PNN100 alleviates demyelination, reduces inflammatory cytokines TNF-α, IL-1β, and restores anti-inflammatory markers (IL-10) in brain homogenates, CSF, and blood plasma. Moreover, PNN normalized neurotransmitter imbalances, including elevated glutamate and reduced GABA, dopamine, serotonin, and acetylcholine levels, highlighting its role in restoring excitatory-inhibitory balance. ELISA studies confirmed PNN ability to modulate apoptotic markers Bax, Bcl-2, and Caspase-3 and upregulate neurotrophic factors GDNF, and GFRA1 while downregulating hyperactivated pathways like AKT, ERK1/2, and GSK3-Beta. Additionally, hematological parameters disrupted by EBRO were significantly restored by PNN, indicating its systemic anti-inflammatory and hematoprotective effects. This research provides the first evidence of PNN’s role in modulating the GDNF/GFRA1/RET/AKT/ERK1/2/GSK3β pathway in MS. Its synergistic action with VB12 underscores its potential as a combinatorial therapeutic strategy. The findings pave the way for innovative treatment approaches to improve outcomes for MS patients by addressing neurodegeneration, inflammation, and systemic immune dysregulation.

## Graphical Abstract

**Figure:**
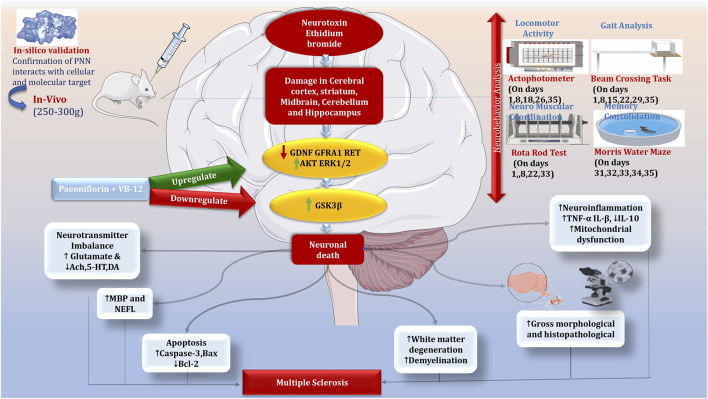

## Introduction

Multiple sclerosis (MS) is a demyelinating autoimmune disease characterised by the loss of the myelin sheath, the formation of demyelinating plaques, and inflammatory and degenerative changes that affect oligodendrocytes (Ahuja et al., 2017; Chhabra and Mehan, 2023; Sharma T. et al., 2024). This leads to white matter injury, demyelination, neuroinflammatory responses, and several pathological mechanisms, including optic neuritis, axonal degeneration, cognitive deficits, loss of learning and memory, oxidative stress, mitochondrial dysfunction, and abnormalities in neurotransmitter levels (Dobson and Giovannoni, 2019; Khan et al., 2024).

MS is a heterogeneous disorder caused by an interaction of genetic and environmental variables and sleep deprivation (Baecher-Allan et al., 2018; Upadhayay and Mehan, 2021; Kumar N. et al., 2023). This includes genetic predisposition, nutritional deficiency, insufficient vitamin D and vitamin B12 levels, smoking, adolescent obesity, immune system dysregulation, bacterial infections, Epstein-Barr virus, gliotoxin, T cell over-activation, and B cell production; they all affect the motor sensory neuron (Kapoor and Mehan, 2020; Sharma T. et al., 2024).

With millions of people affected by MS, a complex inflammatory disease that can cause severe physical and cognitive disability, it is imperative to focus on treatment discovery for MS. With few effective treatment options and a rising number of MS patients, developing new and efficient medicines can significantly improve patient quality of life and meet unmet medical needs. Additionally, advances in molecular pharmacology offer opportunities for more effective treatment of the illness.

Recently, our lab established and validated the EBRO model, resulting in MS-like behavioral and pharmacological alterations in the brain (Chhabra et al., 2023). Animal models are essential for understanding the pathogenic mechanisms, progression, risk factors, and potential treatments for MS (Kumar et al., 2021). EBRO, a neurotoxin, is administered through intracerebropeduncle (ICP) injections into the white matter regions of the brain to induce demyelination in the MS model (Sharma et al., 2021). EBRO disrupts myelin sheaths, oligodendrocytes, and astrocytes by intercalating with chromosomal mitochondrial DNA (Kapoor et al., 2022). It leads to mitochondrial and cognitive dysfunction, destruction of oligodendrocytes and astrocytes, deterioration of the myelin sheath, apoptosis, and axonal degeneration (Singh et al., 2021; Kumar N. et al., 2024). In comparison with EAE model for demyelination the EBRO model reproducible focal and predictable demyelination which is better for studying remyelination after PNN administration. Moreover, preliminary studies have shown that EBRO-ICP injections in the brain produce behavioral, complete blood count (CBC), gross anatomical, neurochemical, and histological abnormalities similar to those observed in MS (Chhabra et al., 2023).

Molecular targets include GDNF, GFRA1, RET, ERK1/2, and GSK3-Beta; these are important in understanding how neurodegenerative diseases work and how to treat them (Tassigny et al., 2015; Yoshino and Ishioka, 2015; Kirouac et al., 2017; Thornton et al., 2018; Jmaeff et al., 2020; Kommaddi et al., 2024; Sahu et al., 2021). A neurotrophic factor called GDNF helps dopaminergic and motor neurons live and grow. This shows that it protects neurons in conditions like Parkinson’s disease (PD) (Drinkut et al., 2016) and ALS (Baloh et al., 2022; Mukherjee et al., 2025; Rana et al., 2025). GFRA1 and its co-receptor modulate the interaction between GDNF and RET, a tyrosine kinase that initiates PI3K/AKT and ERK1/2 signalling pathways essential for neuronal survival, proliferation, and differentiation (Jmaeff et al., 2020; Khan et al., 2025). Neurodegenerative disorders are associated with dysregulation of RET signaling, leading to impaired activation of downstream AKT and ERK1/2 pathways, which contribute to neuroinflammation, neuronal loss, and demyelination (Mol et al., 2023). ERK1/2 is involved in cellular development, differentiation, and apoptosis, and it also contributes to the etiology of AD (Kirouac et al., 2017) and PD (Mesa-Infante et al., 2022). Additionally, this pathway supports synaptic plasticity and stress resistance (Choudhary et al., 2025; Gupta S. et al., 2025). GSK3β is a widely expressed kinase that controls neuronal survival and axonal development (Gupta A. K. et al., 2025). Using drugs to change these pathways, like stopping GSK3-Beta in AKT, could lead to new neuroprotective medicines that prevent neurons from dying and help them grow again (Ramires Júnior et al., 2023). Studies show that decreasing GDNF, GFRA1, RET, AKT, and ERK1/2, and increasing GSK3-Beta pathways are linked to neurodegenerative diseases such as AD (Kirouac et al., 2017), PD (Kaur et al., 2015), ALS (Sahu et al., 2022), and HD (Mehan et al., 2017). There have also been studies that link these changes to neuropsychiatric disorders like depression and schizophrenia, as well as autism (Kumar et al., 2025), and epilepsy (Yamagishi et al., 2024).

Research on many patients with MS has shown changes in the concentrations of multiple antioxidants in brain tissue and cerebrospinal fluid (CSF) (Burgetova et al., 2022). The downregulation of pathways, including GDNF, GFRA1, and RET, coupled with the overexpression of AKT, ERK1/2, and GSK3-Beta signaling, has been implicated (Beaudry-richard et al., 2025). The hyperactivation of GSK3-Beta signaling is frequently linked with neuroinflammation and axonal damage in MS (Leibinger et al., 2017).

Preclinical experiments for MS motivate the use of active phytoconstituents due to their potential to offer fewer side effects than traditional synthetic drugs and to modulate multiple pathways involved in inflammation and neuroprotection (Omrani et al., 2025). Phytoconstituents also synergise to enhance treatment efficacy and improve MS management, thereby improving patient outcomes and therapeutic efficacy. This exploration expands the range of treatment options for managing complex neurological diseases (Zhou et al., 2025).

The root of Paeonia lactiflora Pall yields Paeoniflorin (PNN), a terpenoid glycoside (Chen et al., 2023). It is distributed throughout the plant, such as flowers, stems, leaves, fruits, seeds, and rhizomes. Previous research studies have demonstrated the therapeutic activity of PNN in various diseases, including kidney diseases (Zhang M. Y. et al., 2023), neuroinflammatory bowel diseases (Wu K. C. et al., 2021), rheumatoid arthritis (Yang et al., 2024), hypertension (Yu et al., 2022), gastric cancer (Zhang M. et al., 2023), and allergic diseases (Zhao et al., 2021), as well as its anti-inflammatory and antioxidant properties (Kim et al., 2023).

Various preliminary studies observed that preventive therapeutic effects on various neurological disorders, including AD (Zhang M. et al., 2023), PD (Wu M. et al., 2021). PNN has been found to have therapeutic potential in the model of MS, as it alleviates symptoms in the experimental autoimmune encephalomyelitis (EAE) model (Zhang et al., 2017). PNN inhibits dendritic cell activity, leading to reduced neuroinflammation and demyelination, Th17 cell development, and IL-6 production, all of which are major contributors to MS pathogenesis (Zhang and Wei, 2020).

Previous studies on PNN demonstrated neuroprotective effects in a variety of neurological illnesses by modulating GDNF, GFRA1, RET, AKT, ERK1/2, and GSK3-Beta signaling in subarachnoid hemorrhage (SAH) (Wang T. et al., 2020), and PD (Wang et al., 2022). Another study found that PNN suppresses the production of pro-inflammatory cytokines in Aβ-induced C6 glial cells (Cho et al., 2020).

PNN also shows a neuroprotective effect in various diseases, such as cardiovascular disease (Ren et al., 2023), metabolic disorders, autoimmune illnesses, and cancer (Li et al., 2023; Fang et al., 2024). It modulates signaling pathways such as GDNF/RET, GFRA1, RET, AKT, ERK1/2, and GSK3-Beta to promote endothelial cell survival and vascular regeneration (Mesa-Infante et al., 2022; Sun H. et al., 2024).

PNN has been reported to have neuroprotective activity via modulating GDNF, GFRA1, RET, AKT, ERK1/2, and GSK3-Beta signaling, attenuating myelin degradation, oligodendrocyte mortality, axonal degeneration, and demyelination associated with neurochemical deficiencies leading to MS (Liu et al., 2006; Chen et al., 2017). Preclinical studies found that PNN medication reduced demyelination and disease relapse in an EAE model of relapsing-remitting MS (RRMS) (Zhang et al., 2017). PNN was also shown to exhibit anti-inflammatory and cytoprotective properties in a primary co-culture of rat astroglial and microglial cells (Wang et al., 2024).

Vitamin B12 (Cobalamin) is crucial for brain health and the proper functioning of the nervous system, playing a key role in myelin production, DNA synthesis, and the metabolism of fatty acids and amino acids (Boachie et al., 2021). Deficiency of VB-12 can lead to various brain diseases and neurological disorders, such as AD (Douaud et al., 2013), PD (Schaffner et al., 2019), HD (Pradhan et al., 2023), MS (Jonnalagadda et al., 2023), ALS (Wang M. et al., 2020), Autism (Zhang et al., 2016), and epilepsy (Kathiravan et al., 2021). Leading to cognitive decline, muscle weakness, as well as memory loss and dementia (Aguilar-Navarro et al., 2023). A preclinical study found that reduced serum levels, macrocytosis, and CSF findings must indicate VB-12 deficiency (Beaudry-richard et al., 2025). This results in defective myelin sheath development due to the incorporation of nonphysiological fatty acids into neuronal lipids and the methylation of the major myelin basic protein, MBP (Krugmann et al., 2020).

In this study, we aimed to investigate the neuroprotective effects of PNN, focusing on its potential to safeguard neural health. We conducted a series of *in-vitro* and *in-vivo* experiments to explore the mechanisms underlying PNN neuroprotective properties and to assess its viability as a therapeutic agent for neurological conditions, including MS. Additionally, *in silico* analyses were performed to examine PNN interactions with receptors such as GDNF, GFRA-1, RET, Akt, Erk1/2, and GSK3-Beta. These computational findings were subsequently validated through *in-vitro* and *in-vivo* experiments to confirm the compound’s neuroprotective effects in MS. The outcomes of this study may offer valuable insights into the potential of PNNs to promote neural health and develop new treatments for neurodegenerative diseases and related disorders.

## Materials and methods

### Molecular docking

Molecular docking is key in neurological disease research, aiding drug discovery, understanding mechanisms, and advancing personalized medicine (Chen et al., 2020). In AD, PD, and MS, it identifies targets like enzymes, receptors, and protein aggregates, enabling high-throughput screening of small molecules (Jang et al., 2018). This refines drug candidates to enhance binding affinity. Docking reveals protein-molecule interactions, shedding light on pathways like oxidative stress and inflammation (Khan et al., 2022). It also provides insights into complex targets such as GPCRs and ion channels, guiding therapy design. Docking predicts genetic variations in drug targets, supporting tailored treatments and biomarker identification (Yang et al., 2021). Despite challenges like crossing the BBB, its impact includes identifying inhibitors and modulators in various diseases. The investigation explores the interactions between the drug PNN and selected molecular targets, utilizing the crystal structures of the GDNF receptor (https://www.rcsb.org/structure/1AGQ) (PDB ID: 1AGQ) (Eigenbrot and Gerber, 1997), GFRA-1 receptor (https://www.rcsb.org/structure/1q8d) (PDB ID: 1Q8D) (Leppänen et al., 2004), RET receptor (https://www.rcsb.org/structure/2ivu) (PDB ID: 2IVU) (Knowles et al., 2006), Akt receptor (https://www.rcsb.org/structure/3O96) (PDBID: 3O96) (Wu et al., 2010), Erk1/2 receptor (https://www.rcsb.org/structure/6GDQ) (PDB ID: 6GDQ) (Heightman et al., 2018), and GSK3-Beta receptor (https://www.rcsb.org/structure/1q41) (PDB ID: 1Q41) (Bertrand et al., 2003) from the RCSB Protein Data Bank. Before molecular docking, several preparatory steps were performed on the retrieved crystal structure. These included removing water molecules and ions and adding nonpolar hydrogens (Pratihar et al., 2023). The protein underwent minimization and optimization using AutoDock Tool v1.5.7 (https://autodocksuite.scripps.edu/adt) as an initial step (Morris et al., 2010). Following energy minimization, the reference drug PNN was converted into the pdbqt format using PyRx, and molecular docking was subsequently conducted with the PyRx virtual screening tool (https://sourceforge.net/projects/pyrx) (Dallakyan and Olson, 2015). To ensure efficient docking, standard-sized grids were carefully positioned at the default locations identified by the docking algorithm within the receptor’s active site pocket of (PDB ID: 1AGQ) at the coordinates X = 16.69, Y = 29.01, Z = 16.44, (PDB ID: 1Q8D) X = 14.61, Y = 29.86, Z = 17.21, (PDB ID: 2IVU) X = −28.24, Y = 1.75, Z = −17.91, (PDB ID: 3O96) X = 9.92, Y = −5.29, Z = 14.19, (PDB ID: 6GDQ) X = −0.006, Y = 6.08, Z = 40.41, and (PDB ID:1Q41) X = 27.74, Y = 8.06, Z = 39.49. Interactions in the most favorable docking poses were analyzed using Discovery Studio, version 21.1.0.20298 (Kumar S. et al., 2023).

### Ethical recommendation

Sixty-four adult male Wistar rats, each weighing between 250 and 300 g, were utilised in this investigation. Male rats were specifically used to minimize hormonal variability associated with the oestrous cycle of females rats, which may influence neuroinflammatory responses. The rats were obtained from the Central Animal House of the ISF College of Pharmacy, Moga, Punjab. The experimental protocol was approved by the Institutional Animal Ethics Committee (IAEC) under registration number 816/PO/ReBiBt/S/04/CPCSEA, identified as protocol number ISFCP/IAEC/CCSEA/Meeting No.06/12/2024/Protocol No.52, in accordance with the guidelines established by the Government of India. The rats were housed in polyacrylic cages measuring 38 cm × 32 cm × 16 cm, with two rats per cage, and were supplied with soft bedding. The animals were maintained under standardized husbandry and settings, featuring unlimited access to food and water, humidity levels of 55% ± 10%, a temperature of 22 °C ± 2 °C, and a 12-h light and dark cycle with automated lighting. The rats were allowed to acclimate for 7 days before the start of the trial (Mehan et al., 2018; Duggal et al., 2020).

### Chemicals and drugs

Neurotoxin ethidium bromide was obtained from Sigma-Aldrich (U.S.A.). PNN was sourced from the Drug Discovery and Development Centre at RTI International in North Carolina, USA. PNN was dissolved in 0.9% normal saline and administered orally (Gu et al., 2016; Zhang et al., 2017; Cheng et al., 2021). VB12 was purchased from Sun Pharma, Mumbai, India. The adjuvant VB12 was given orally and dissolved in 0.9% normal saline (Erfanparast and Tamaddonfard, 2015; Ikeda et al., 2015; Al-Khamis, 2016). All chemicals used in the experiment were of HPLC grade. Before use, fresh drugs and chemicals were meticulously prepared.

### Experimental protocol timeline

The experiment lasted for 35 days; 0.1% EBRO dissolved in 0.9% saline unilaterally was given to the experimental rats through the intracerebropeduncle (ICP) route to induce multiple sclerosis (MS). The ICP-EBRO treatment was administered from day 1 to day 7. From day 8 to 35, PNN was administered orally at two doses (50 mg/kg and 100 mg/kg, *p.o*.). The adjuvant drug VB12 (30 mg/kg, *p.o.*) was also given orally. Several animal behavioral tests were carried out throughout the experimental protocol, which was carried out from day one to day 35th, such as the rotarod test (RRT), beam crossing task (BCT), locomotor activity (LA) measured with an actophotometer, and the Morris water maze (MWM). These assessments were conducted on designated days to evaluate the phenotypic traits of multiple sclerosis (MS) in the experimental rats. The animals were deeply anesthetized and euthanized on day thirty-six after completing the experimental procedures. Their brains were carefully removed and preserved in formalin. Following this, the brains were analyzed for biochemical evaluations, gross pathological changes, histological alterations, and Luxol fast blue staining to identify any reduction in white matter fibers (Kumar et al., 2021; Sharma and Mehan, 2021; Singh et al., 2021; Kapoor et al., 2022; Khera et al., 2022; Upadhayay et al., 2022) Figure 1.

**FIGURE 1:**
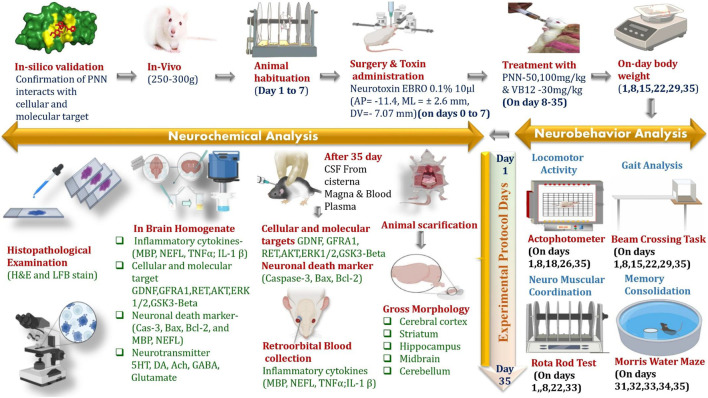
Experimental Protocol schedule.

### Experimental animal grouping

The animals used in the experiment were randomly divided into eight groups: Group 1: Sham Control; Group 2: Vehicle Control; Group 3: PNN Perse (100 mg/kg, *p.o.*); Group 4: EBRO (10 μL, *i.c.p*.); Group 5: EBRO (10 μL, *i.c.p.*) + PNN (50 mg/kg, *p.o.*); Group 6: EBRO (10 μL, *i.c.p.*) + PNN (100 mg/kg, *p.o.*); Group 7: EBRO (10 μL, *i.c.p.*) + VB12 (30 mg/kg, *p.o.*); Group 8: EBRO (10 μL, *i.c.p.*) + VB12 (30 mg/kg, *p.o.*) + PNN (100 mg/kg, *p.o.*). The sham control group is included to eliminate the bias that may be due to the surgical procedure, whereas the vehicle control group is included to ensure that the observed therapeutic effects are due to PNN and not the solvent used. Inclusion of both groups ensured accurate differentiation between procedural and treatment-related effects, thereby improving experimental transparency.

### Experimental model of MS

Mehan and colleagues utilized a well-established and validated experimental model of multiple sclerosis induced by EBRO in this study (Kumar et al., 2021; Sharma and Mehan, 2021; Singh et al., 2021; Kapoor et al., 2022; Upadhayay et al., 2022). Wistar rats were habituated to laboratory conditions after being retrieved from the animal facility. Before the surgery began, a sterile environment was established. To induce anesthesia, the rats received an intraperitoneal injection of 75 mg/kg of ketamine. Once anesthetized, the rat was placed on a sterile, heated platform connected to a well-equipped rodent ventilator (Giri et al., 2023). To prevent contamination, the entire scalp of the anesthetized rats was treated with a Povidone-iodine solution and then wiped clean. The scalp was shaved, and a small mid-sagittal incision was made to expose the soft tissues. Cotton balls soaked in normal saline were continuously applied to the wound to control excessive blood loss. The rats’ eyes were lubricated with CMC eye solutions to prevent dehydration. The skull was exposed, and the rat’s head was affixed to the stereotaxic frame using the ear and incision bars (Stoelting Co., Wood Dale, IL, USA). The positions of lambda and bregma were identified to determine the injection coordinates for ICP (Mehan et al., 2020).

### Cannula implantation

A burr hole was created in the skull, and a 2.5 mm cannula was inserted at the designated stereotaxic coordinates: DV (dorsal/ventral) = 7.07 mm; AP (anterior/posterior) = 11.6 mm; ML (medial/lateral) = 2.6 mm. The cannula was quickly positioned into the rat’s skull and secured with dental cement. A surgical suture and needle were used to close the incision. Before each ICP injection, a removable plastic ear pin was utilized to stabilize the cannula. A calibrated Hamilton syringe was then used to unilaterally deliver 10 μL of 0.1% EBRO in 0.9% saline to the experimental rats (Chhabra et al., 2023; Kumar et al., 2021).

### Post-surgical treatment

After surgery, the experimental rats were given personalized care in polyacrylic enclosures lined with soft towels until they regained consciousness, which took about 1–2 h post-anesthesia. The temperature was maintained at 25 °C ± 3 °C. To reduce physical stress, glucose water and milk were provided in the polyacrylic cages for two to 3 days. To prevent septic infection after surgery, each rat received an intraperitoneal injection of gentamicin (40 mg/kg) twice daily, and lignocaine gel was applied to the sutured area to alleviate pain. Neosporin powder was applied to the skin for 3 days to prevent contamination. From day 1 to day 7, the intracerebropeduncle pathway (ICP) was injected with 10 μL of 0.1% EBRO over 10 min at a rate of 1 μL/min, using a 0.4 mm outer diameter hypodermic needle connected to a hamilton microliter syringe 10 μL. After the injection, the Hamilton syringe was held for 5 min to allow diffusion of the neurotoxin into the cerebrospinal fluid and to prevent backflow. After a week, the rats were given a nutritionally balanced diet of water and chow. All physiological parameters were monitored daily, including weight changes, signs of dehydration, potential infections, and other clinical symptoms (Khera et al., 2022; Upadhayay et al., 2022).

### Evaluated parameters

#### Neurobehavioral experiments

##### Rotarod performance test

The Rotarod test (INCO Group of Companies, Ambala) was used to evaluate motor coordination and balance across treatment groups. This test was performed on the 1st, 8th, 22nd, and 33rd days of the experimental protocol. RRT speed was set at 15 rpm, and the time (in seconds) that each rat fell was recorded over 5 min (Kapoor et al., 2022).

##### Beam crossing task (BCT)

The beam-crossing test (INCO Group of Companies, Ambala) was conducted across various treatment groups to assess the animals’ susceptibility to stress induced by height and motor coordination. Each animal was evaluated for locomotion, foot slippage, and neuromuscular coordination on days 1, 8, 15, 22, 29, and 35. The number of foot slips per rat per trial was recorded for 2 min (Tiwari et al., 2021).

##### Locomotor activity

Wistar rat locomotor activity was measured using an Actophotometer (Swastika, India). The spontaneous animal locomotor activity of the experimental animals was assessed on days 1, 8, 18, 26, and 35. Each animal was placed in the center of the instrument. The readings recorded by the automated actophotometer during 5-min intervals reflected locomotion and were counted every 5 minutes (Kumar et al., 2025).

##### Morris water maze (MWM)

The Morris water maze test (MWM) is a spatial navigation task used to assess experimental animals’ memory and spatial learning. This task is closely linked to cognitive impairments and synaptic plasticity in the hippocampus. Rats were given access to a swimming area, and their escape latency time (ELT) to reach underwater platform was measured on days 31, 32, 33, 34. On the 35th day, a memory consolidation assessment was conducted. The platform was removed, and the time rats spent in the quadrant where the platform had previously been located was recorded for 120 s. The time spent in the target quadrant (TSTQ) was analyzed, indicating a degree of memory consolidation (Singh et al., 2021).

#### Assessment of weight alterations

##### Rat body weight

The primary diagnostic indicator of disease progression is a decrease in body weight. The body weight of the rat was measured on days 1, 8, 15, 22, 29, and 35 of the study (Minj et al., 2021).

##### Relative brain-to-body weight ratio

After the experimental rats were sacrificed on day 36 of the study, their brain weights were measured. A brain-to-final body weight ratio was calculated to assess EBRO-induced changes in brain mass in MS rats. The relative brain-to-body weight ratio is calculated as (brain weight/body weight) × 1,000 (Mukherjee et al., 2025).

#### Neurochemical testing

##### Isolation, preparation, and storage of samples and tissues

On day 36th, the rats were anesthetized with Ketamine (75 mg/kg, i.p.) until fully sedated, as confirmed by the toe-pressing reflex. Their skulls were shaved and sterilized to allow non-invasive cerebrospinal fluid collection. The rats were positioned on a specialized table, and their snouts and mouths were placed into a conical aperture.

CSF extraction was performed using a specially designed curved needle, detaching a 30G insulin syringe needle and bending its tip with metal forceps to create an angle of 90–120°. After this, the infusion catheter of the intravenous setup was disconnected, and the narrow tubing of the infusion set was attached to the curved 30G needle. A 1 mL needleless syringe was then connected to the other end. Once the location of the foramen magnum was identified, the curved needle was inserted around its edge to access the cerebellomedullary cistern. The needle length was carefully chosen to avoid damaging the brain tissue. CSF was extracted by pulling back the syringe plunger connected to the flexible tubing while keeping the curved needle steady within the rat’s skull. After collecting the cerebrospinal fluid, the needle was gently withdrawn, and the tubing was removed from the syringe. The CSF was transferred to a microcentrifuge tube in volumes ranging from 70 to 120 μL. This minimally invasive method allows the animals to resume their normal activities within 5–10 min (Bergadano et al., 2019).

### Blood collection

Blood was then collected from the rat via the retro-orbital route using a capillary tube. The capillary tube was inserted behind the nictitating membrane at the inner corner of the eye, carefully puncturing the sinus. Blood was drawn from the capillary tube into a sterile and EDTA-containing tube through capillary action using careful rotation and retraction. A blood sample in an EDTA tube was used for a comprehensive CBC analysis on a veterinary automated haematology analyser. Additional blood samples in tubes were centrifuged at 10,000 g for 15 min to separate the plasma. The plasma was then stored in a deep freezer at −80 °C for subsequent neurochemical analyses. Rats were given sodium phenobarbital (270 mg/mL, *i.p.*) and were put under deep and sacrificed by decapitation. After the rats were euthanised, their whole brains were removed and rinsed with ice-cold isotonic saline.

Some brains were preserved in 4% paraformaldehyde for histological and gross pathological examinations. After a sagittal incision, the remaining brains had their anatomical components extracted with micro spatulas. These components were homogenized with chilled 0.1 M (w/v) phosphate-buffered saline at pH 7.4. After another centrifugation at 10,000 g for 15 min, the supernatant was carefully collected from the brain homogenate. Aliquots were stored in a deep freezer at −80 °C for later biochemical analysis (Bhalla and Mehan, 2022).

### Complete blood analysis

Using a Veterinary Auto Haematology Analyser, we conducted a thorough blood analysis that measured various parameters such as hemoglobin, total leukocyte count, red blood cell count, hematocrit (HCT), mean corpuscular volume (MCV), mean corpuscular hemoglobin (MCH), mean corpuscular hemoglobin concentration (MCHC), platelet count, and a differential leukocyte count, which identifies different types of white blood cells (neutrophils, lymphocytes, monocytes, eosinophils, and basophils). Blood samples were collected by retro-orbital puncture and placed into EDTA containers to prevent coagulation and ensure accurate analysis. Each CBC sample typically contained 10–60 μL of blood, providing sufficient volume to precisely measure all parameters (Gumbar et al., 2023).

### Gross pathological analysis

On day 36th, after the experimental regimen ended, the animals were euthanized by intraperitoneal injection of sodium phenobarbital (270 mg/mL, i.p.) followed by decapitation. Their brains were removed from the skulls, stored in chilled normal saline, and subjected to a thorough pathological examination. A digital camera performed a macroscopic analysis of the entire brain, including all coronal sections. The grey area surrounding these sections was measured to calculate the volume of demyelinated tissue (mm3) for each brain segment. To assess the degree of white matter degeneration, the volume of demyelination for each coronal segment was determined using the formula (length × breadth × height) (Singh et al., 2021).

### ELISA assay

ELISA kits were used to measure different parameters. The brain, CSF, and blood plasma samples were quantified by comparison with the standard curves. Each sample in every group was measured in duplicate or triplicate. Brain homogenates were mixed with phosphate-buffered saline and then centrifuged at 10,000 rpm for 5 min at 4 °C. In accordance with the manufacturer’s guidelines, the supernatants were carefully removed. The ELISA kits for evaluating cellular and molecular targets included GDNF [E-EL-H1495; Elabscience GFRA1 [PKSH033670; Elabscience]; RET [AN00810P; Elabscience], AKT [E-EL-R0807 98T, Elabscience, Wuhan, China] (Sharma and Mehan, 2021); ERK1/2 [E-AB-70292; Elabscience] (Singh et al., 2021); and GSK3-Beta [KLR0989, KRISHGEN, Maharashtra, India] (Sharma R. et al., 2024).

For measuring MBP [E-EL-R0642; Elabscience, Wuhan, China] and NEFL [E-EL-R2536; Elabscience, Wuhan, China] (Kumar S. et al., 2024). We assessed apoptotic markers, including Bcl-2 [KLR1880; Krishgen Biosystem, Mumbai, India], Bax [KLR0034; Krishgen Biosystem, Mumbai, India], and Caspase-3 [KLR1648; Krishgen Biosystem, Mumbai, India] (Tiwari et al., 2021).

Additionally, neurotransmitters like dopamine [KLR0219; Krishgen Biosystem, Mumbai, India], glutamate [KLR1474; Krishgen Biosystem, Mumbai, India], serotonin [KLR0866; Krishgen Biosystem, Mumbai, India], acetylcholine [KLR0722; Krishgen Biosystem, Mumbai, India] (Albekairi et al., 2022), and GABA [KLR0102; Krishgen Biosystem, Mumbai, India] were evaluated (Jadaun et al., 2022). Also assessed neuroinflammatory cytokines TNF-alpha [KB1145; Krishgen Biosystem, Mumbai, India] and IL-1 Beta [KLR0119; Krishgen Biosystem, Mumbai, India] (Mehmood Siddiqui et al., 2021), and IL-10 [GENLISA, Krishgen, Maharashtra, India] (Rajdev et al., 2020; Alharbi et al., 2022).

### Histopathological examination

On day 36th, experimental Wistar rats were euthanized and sacrificed. Their brains were removed and preserved for histopathological analysis. After extraction from the skull, each brain was carefully cleaned and cut into 5-mm segments. Coronal slices of the cerebral cortex, hippocampus, striatum, midbrain, and cerebellum were stained with hematoxylin and eosin (H&E) to identify any inflammatory infiltration and abnormalities. The samples were then placed in 4% paraformaldehyde in PBS at pH 7.4 for 8–12 h at room temperature. The specimens were washed with PBS and immersed in 70% ethanol. The tissue was kept at 37 °C until paraffin embedding was complete. The paraffin blocks were sectioned at 5 μm using a rotary microtome. These sections were stained with hematoxylin and eosin (H&E), and their morphology was examined using a digital microscope (MOTICAM-Ba310 image plus 2.0) at ×40 magnification. The standard neuronal population was evaluated at all coronal slice locations using a fluorescent microscope with an ocular featuring a reticule at a magnification of ×40 (Shandilya et al., 2022; Upadhayay et al., 2022).

### Examination of LFB

The cerebral cortex, striatum, hippocampus, midbrain, and cerebellum were examined for signs of demyelination, and coronal brain sections were stained with LFB to highlight myelinated areas. The paraffin-embedded sections were carefully deparaffinized by immersion in xylene for 10 min each, three times. They were then soaked in 100% ethanol three times, for 5 min each, to remove xylene prior to further processing. The next step involved hydration with 70% ethanol for 5 min. Before staining the myelinated areas, the dewaxed tissue was thoroughly washed with distilled water. It was then placed in a 0.1% Luxol Fast Blue solution, which consisted of 1 g of LFB dye, 1,000 mL of 95% ethanol, and 5 mL of a 10% acetic acid solution. This mixture was incubated overnight at 38 °C (not longer than 16 h). The next day, after a 1-min ethanol rinse, the samples were washed with distilled water. Additionally, 0.05% lithium carbonate was used during the differentiation process, which lasted 5–20 s. Following this slide, 70% ethanol was immersed in 3 times. The procedure was done for 1 minute in the first two Petri dishes. The samples were kept in the third petri dish for 1–10 min. A microscopic examination confirmed the transformation of grey matter into white matter. The slide was rinsed with distilled water. Dehydration was performed for 5 min using 70% and 95% ethanol. Each sample was dehydrated by three 5-min exchanges in 100% ethanol. The slide was cleaned three times with xylene for over 10 minutes. Finally, a coverslip was placed on the slide, which was then analyzed at 40× magnification using a digital microscope (MOTICAM-Ba310 image plus 2.0) (Bhalla and Mehan, 2022; Sharma R. et al., 2024).

### Statistical analysis

A two-way ANOVA with a *post hoc* Bonferroni test was used to analyze the statistical data. The statistical significance level was set at p < 0.01, and the data were displayed as mean ± standard deviation (SD). Each experimental group consisted of eight wistar rats (n = 8). β v/s Sham Control, Vehicle Control, and PNN Perse; δ v/s EBRO; δα1 v/s EBRO + PNN50; δα2 v/s EBRO + PNN100, EBRO + PNN50; and δα3 v/s EBRO + VB12 (30), EBRO + PNN100, EBRO + PNN50.

## Results

### *In-silico* studies

#### Molecular docking analysis

**FIGURE 2:**
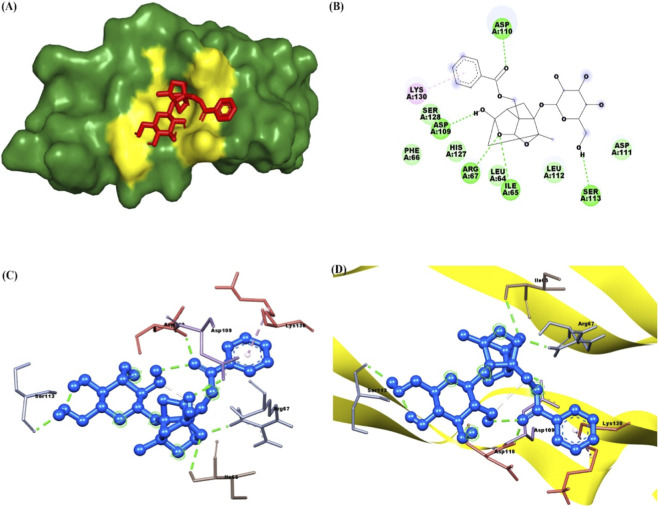
The figures illustrate the docking interaction of paeoniflorin with the GDNF receptor (PDB ID: 1AGQ). **(A)** Shows a surface representation of the GDNF receptor, with the active site highlighted in yellow, where the ligand paeoniflorin (red) is bound. **(B)** 2D interaction diagram displays key interactions between paeoniflorin and the receptor, with hydrogen bonds and hydrophobic interactions clearly indicated. **(C)** Provides a 3D view of the docking, showing paeoniflorin (blue) interacting with GDNF, with hydrogen bonds represented by green dashed lines and key regions labeled. **(D)** Offers a zoomed-in view of the binding site, emphasizing hydrogen bonds and hydrophobic contacts, depicted within a ribbon structure for clarity.

**FIGURE 3:**
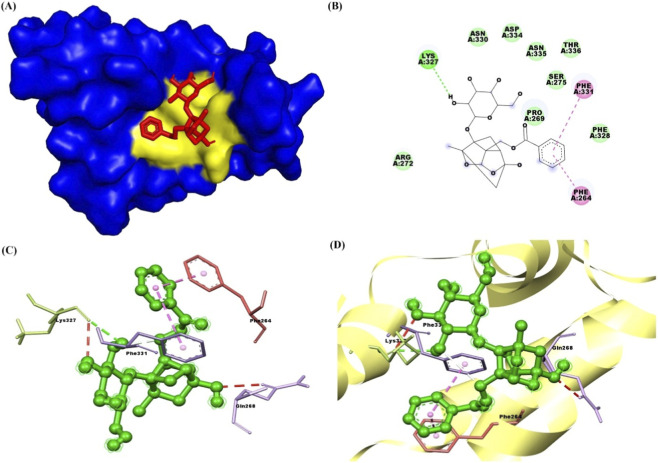
The figures illustrate the docking interaction of paeoniflorin with the GFRA-1 receptor (PDB ID: 1Q8D). **(A)** Shows a surface representation of GFRA-1, with the active site highlighted in yellow, where paeoniflorin (red) is bound. **(B)** Provides a 2D interaction diagram, highlighting key hydrogen bonds and hydrophobic contacts between paeoniflorin and the receptor. **(C)** 3D model depicts the docking of paeoniflorin (green) within GFRA-1, emphasizing the key interaction points. **(D)** Zooms in on the binding site, showing detailed interactions such as hydrogen bonds and hydrophobic contacts, with the receptor represented in a ribbon format.

**FIGURE 4:**
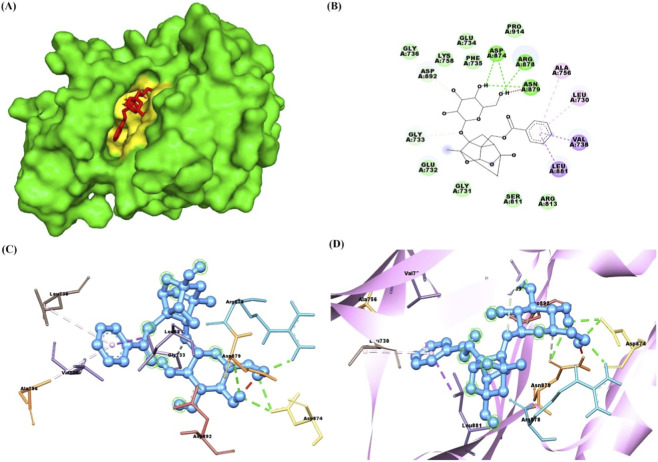
The figures depict the docking interaction of paeoniflorin with the RET receptor (PDB ID: 2IVU). **(A)** Shows a surface representation of RET with paeoniflorin (red) bound in the active site, highlighted in yellow. **(B)** Presents a 2D interaction diagram, illustrating key hydrogen bonds and hydrophobic contacts between paeoniflorin and the receptor. **(C)** 3D model reveals paeoniflorin (blue) docked within RET, emphasizing important interaction sites. **(D)** Provides a close-up of the binding interface, focusing on hydrogen bonds and hydrophobic interactions, represented within a ribbon diagram for clarity.

**FIGURE 5:**
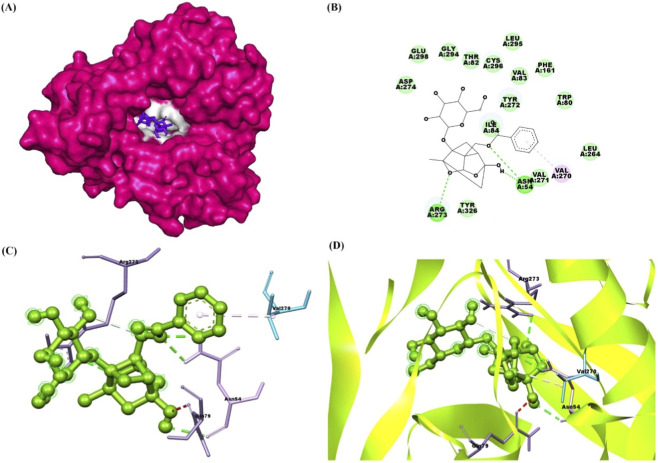
The figures depict the docking interaction of paeoniflorin with the Akt receptor (PDB ID: 3O96). **(A)** Shows a surface representation of Akt with paeoniflorin (purple) bound in the active site, highlighted in white. **(B)** Presents a 2D interaction diagram, illustrating key hydrogen bonds and hydrophobic contacts between paeoniflorin and the receptor. **(C)** 3D model displays paeoniflorin (green) docked within Akt, emphasizing important interaction points. **(D)** Offers a close-up of the binding site, focusing on hydrogen bonds and hydrophobic interactions, represented within a ribbon diagram for clarity.

**FIGURE 6:**
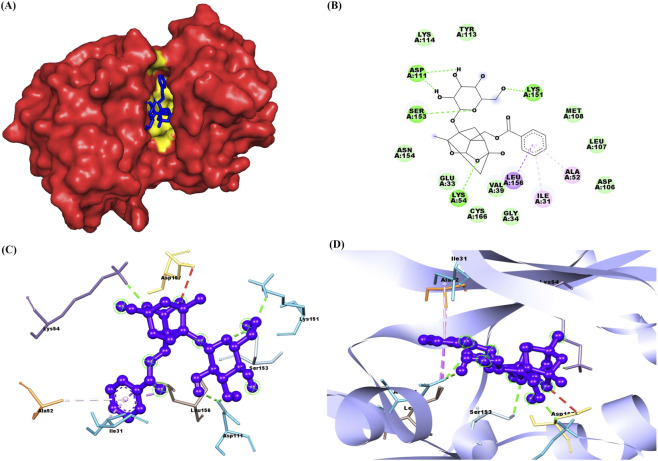
The figures depict the docking interaction of paeoniflorin with the Erk1/2 receptor (PDB ID: 6GDQ). **(A)** Shows a surface representation of Erk1/2 with paeoniflorin (blue) bound in the active site, highlighted in yellow. **(B)** Presents a 2D interaction diagram, illustrating key hydrogen bonds and hydrophobic contacts between paeoniflorin and the receptor. **(C)** 3D model highlights paeoniflorin (purple) docked within Erk1/2, showing important interactions. **(D)** Provides a close-up view of the binding site, focusing on hydrogen bonds and hydrophobic interactions, represented within a ribbon diagram.

**FIGURE 7:**
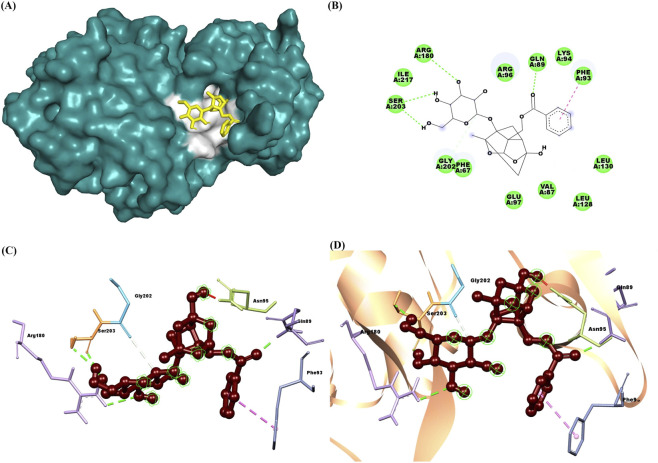
The figures illustrate the docking interaction of paeoniflorin with the GSK3-Beta receptor (PDB ID: 1Q41). **(A)** Shows a surface representation of GSK3-β with paeoniflorin (yellow) bound in the active site, highlighted in white. **(B)** Presents a 2D interaction diagram, showing key hydrogen bonds and hydrophobic contacts between paeoniflorin and the receptor. **(C)** 3D model displays paeoniflorin (brown) docked within GSK3-β, highlighting important interactions. **(D)** Provides a close-up view of the binding site, focusing on hydrogen bonds and hydrophobic interactions, depicted within a ribbon diagram.

The docking results of PNN with the GDNF receptor (PDB ID: 1AGQ) reveal a strong and specific interaction, supported by a docking score of −6.7 kcal/mol, indicating a favorable binding affinity. The ligand efficiency, calculated at 0.269, suggests a reasonably efficient interaction between the ligand and the receptor relative to its size. Paeoniflorin forms van der Waals interactions with key residues such as Ser128, Phe66, His127, Leu64, Leu112, and Asp111, stabilizing the ligand within the binding site. Additionally, conventional hydrogen bonds are formed with residues Ser113, Ile65, Arg67, Asp109, and Asp110, further enhancing the binding affinity and specificity through strong directional interactions. Pi-alkyl interactions with Lys130 also contribute to stability by providing hydrophobic contact points that reinforce the ligand’s position. These interactions suggest that paeoniflorin binds effectively to GDNF, potentially modulating its activity and signaling. Figure 2 shows paeoniflorin docking with the GDNF receptor (PDB ID: 1AGQ), highlighting key interactions, including hydrogen bonds and hydrophobic contacts. The molecular docking analysis of PNN with GFRA-1 receptor (PDB ID: 1Q8D) indicates several key interactions that support the stability of the ligand-receptor complex, with a docking score of −7.8 kcal/mol and a ligand efficiency of 0.314. PNN forms a conventional hydrogen bond with Lys 327, which is crucial for establishing a strong polar interaction and anchoring the ligand in the binding pocket. Additionally, Pi-Pi interactions are observed between Phe 264 in a T-shaped configuration and Phe 331 in a stacked arrangement, further enhancing binding affinity through aromatic stacking. Van der Waals interactions also play a significant role in stabilizing the ligand through residues like Asn 330, Asp 334, Asn 335, Thr 336, Ser 275, Pro 269, Arg 272, and Phe 328. These interactions provide a complementary environment that helps secure paeoniflorin in its optimal binding conformation. The combination of hydrogen bonds, Pi-Pi stacking, and Van der Waals forces, along with the docking score and ligand efficiency, suggests a favorable binding affinity of paeoniflorin with GFRA-1, highlighting its potential as a modulator of the receptor for therapeutic purposes. Figure 3 shows paeoniflorin docking with the GFRA-1 receptor (PDB ID: 1Q8D), highlighting key interactions, including hydrogen bonds and hydrophobic contacts, with surface, 2D, 3D, and zoomed-in views of the binding site. The docking results for PNN with the RET receptor (PDB ID: 2IVU) reveal a complex network of interactions that contribute to the stability and binding affinity of the ligand within the receptor’s active site. Van der Waals interactions, involving residues such as Gly 731, Glu 732, Gly 736, Glu 734, Phe 735, Lys 758, Ser 811, Arg 813, and Pro 914, play a crucial role in stabilizing PNN by providing surface complementarity. Additionally, conventional hydrogen bonds with Asp 874, Arg 878, and Asn 879 further strengthen the ligand’s binding, positioning it effectively within the receptor. Carbon-hydrogen bonds, involving Asp 892, Gly 733, and Asn 879, contribute additional stabilizing interactions. Hydrophobic interactions, such as Pi-sigma interactions with Leu 881 and Val 738, and Pi-alkyl interactions with Leu 730 and Ala 756, enhance the ligand’s affinity for the RET receptor by promoting favorable hydrophobic contacts. The docking score of −8.8 kcal/mol indicates a strong binding affinity, and the ligand efficiency of 0.354 further supports the favorable interaction between PNN and RET. Figure 4 illustrates paeoniflorin binding to the RET receptor (PDB ID: 2IVU), featuring surface, 2D, 3D, and close-up views that highlight key hydrogen bonds and hydrophobic interactions at the active site. The molecular docking results of PNN with Akt receptor (PDB ID: 3O96) reveal a docking score of −8.3 kcal/mol, indicating a favorable and strong binding affinity between the ligand and the receptor. The interactions between PNN and Akt are stabilized by key conventional hydrogen bonds with Arg 273 and Asn 54, which play a critical role in anchoring the ligand within the receptor’s active site. Additionally, a pi-alkyl interaction with Val 270 further enhances the ligand’s stability through hydrophobic interactions. A network of van der Waals interactions involving residues such as Glu 298, Gly 294, Thr 82, Asp 274, Cys 296, Leu 295, Val 83, Phe 161, Tyr 272, Ile 84, Val 271, Leu 264, Trp 80, and Tyr 326 contributes to a snug fit within the binding pocket. These non-covalent interactions collectively support the proper positioning of the ligand and enhance binding stability. Furthermore, the ligand efficiency of 0.334 indicates that paeoniflorin effectively interacts with Akt, balancing binding affinity with molecular size. These results suggest that paeoniflorin may effectively modulate Akt activity, with therapeutic implications for cellular processes such as growth and survival. Figure 5 illustrates PNN docking with the Akt receptor (PDB ID: 3O96), showcasing surface, 2D, 3D, and close-up views. Key hydrogen bonds and hydrophobic interactions at the active site are highlighted for clarity. In the molecular interaction analysis, several types of bonding interactions are observed between the ligand and the surrounding residues. Conventional hydrogen bonds are identified with the residues Asp 111, Ser 153, Lys 54, and Lys 151, indicating strong electrostatic interactions that play a significant role in stabilizing the ligand within the binding site. Pi-alkyl interactions are noted with Ile 31 and Ala 52, reflecting hydrophobic interactions that contribute to the non-polar nature of the binding environment. Additionally, a pi-sigma interaction is found with Leu 156, which typically aids in stabilizing the aromatic moiety of the ligand. The van der Waals interactions are observed with several residues, including Lys 114, Tyr 113, Met 108, Leu 107, Asn 154, Glu 33, Val 39, Cys 166, Gly 34, and Asp 106. These weaker, yet significant, interactions provide additional stabilization and shape complementarity within the binding pocket. The overall docking score for this complex is −7.7 kcal/mol, suggesting a favorable binding affinity, while the ligand efficiency is 0.310, indicating a reasonable balance between molecular size and binding affinity for the ligand in this docking configuration. Figure 6 illustrates paeoniflorin docking with the Erk1/2 receptor (PDB ID: 6GDQ), featuring surface, 2D, 3D, and close-up views. It highlights key hydrogen bonds and hydrophobic interactions at the active site for clarity. The docking results of paeoniflorin with the GSK3-β receptor (PDB ID: 1Q41) reveal several key interactions, along with a docking score of −7.9 kcal/mol and a ligand efficiency of 0.318, indicating a stable and potentially high-affinity binding. Conventional hydrogen bonds involving Ser 203, Arg 180, and Gln 89 suggest strong anchoring of paeoniflorin within the active site, contributing to the specificity and stability of the interaction. A pi-pi stacking interaction with Phe 93 further enhances this stability by providing additional non-covalent binding forces. The Van der Waals interactions with residues such as Ile 217, Arg 96, Phe 67, Lys 94, Val 87, Glu 97, Leu 130, and Leu 128 indicate that paeoniflorin fits well into the binding pocket, contributing to the overall binding strength through numerous close contacts. Additionally, the carbon-hydrogen bond with Gly 202 further stabilizes the ligand’s orientation. The combination of these interactions, along with the favorable docking score, suggests that paeoniflorin has a strong affinity for GSK3-β, which could inhibit the kinase’s activity, highlighting its potential as a therapeutic agent targeting this enzyme. Figure 7 illustrates paeoniflorin docking with the GSK3-β receptor (PDB ID: 1Q41), showcasing surface, 2D, 3D, and close-up views. It highlights key hydrogen bonds and hydrophobic interactions at the active site for clarity.

### Neuroprotection by PNN in the reduction of behavioral changes in EBRO-induced rat model of multiple sclerosis

#### PNN improves locomotion behaviour

**FIGURE 8:**
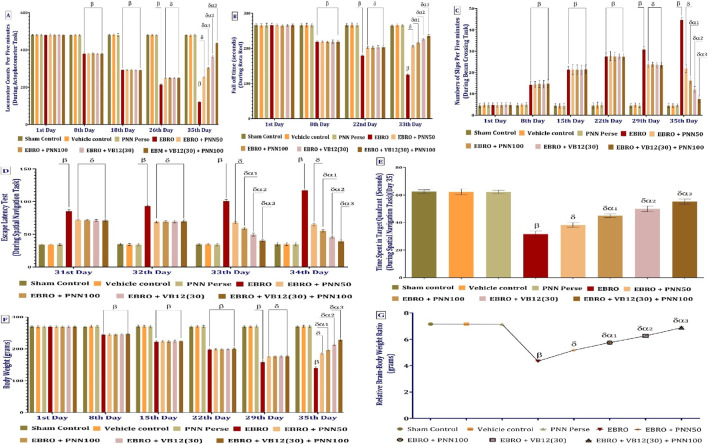
**(A–G)** PNN neuroprotective role in mitigating EBRO-induced behavioral alterations in MS rat model: Locomotion Behaviour **(A)**; Fall off time **(B)**; Number of foot slips **(C)**; Escape latency time **(D)**; TSTQ **(E)**; Body weight **(F)** and relative brain-body ratio **(G)**. To identify significant differences between groups, a one-way ANOVA and Tukey’s *post hoc* test were used for statistical analysis **(E,G)**. The statistical significance level was set at p < 0.01, and the data were displayed as mean ± standard deviation (SD). There were eight wistar rats (n = 8) in each experimental group. β v/s Sham Control, Vehicle Control, and PNN Perse; δ v/s EBRO; δα1 v/s EBRO + PNN50; δα2 v/s EBRO + PNN100, EBRO + PNN50; and δα3 v/s EBRO + VB12 (30), EBRO + PNN100, EBRO + PNN50. A two-way ANOVA with a *post hoc* Bonferroni test was used to analyze the statistical data **(A–F)**. The statistical significance level was set at p < 0.01, and the data were displayed as mean ± standard deviation (SD). Each experimental group consisted of eight wistar rats (n = 8). β v/s Sham Control, Vehicle Control, and PNN Perse; δ v/s EBRO; δα1 v/s EBRO + PNN50; δα2 v/s EBRO + PNN100, EBRO + PNN50; and δα3 v/s EBRO + VB12 (30), EBRO + PNN100, EBRO + PNN50.

On the 1st, 8th, 18th, 26th, and 35th days of the experimental protocol, locomotor behaviour was measured with an actophotometer. There were no appreciable variations in the groups’ movements on the 1st day. In contrast to the Sham Control, Vehicle Control, and PNN Perse group, the EBRO-induced group showed decreased locomotor activity by the 8th day. By day 26th, the EBRO + PNN50 and EBRO + PNN100 groups exhibited increased locomotor activity compared to the EBRO-induced group. The EBRO + VB12 (30) and EBRO + PNN100 groups exhibited comparable improvements. On the 35th day, the PNN100 group demonstrated greater enhancement in locomotor behaviour compared to the PNN50 group, suggesting a dose-dependent effect of PNN on activity levels. Additionally, the combination group exhibited a markedly greater increase in locomotor activity than the PNN monotherapy groups, highlighting the potentiating effect of EBRO + VB12 (30) when administered with high-dose PNN. The analysis of the locomotion activity involved a two-way ANOVA, which produced an F-statistic of F (7,56) = 13,006, (p < 0.01), Figure 8A.

#### PNN reduces the fall-off time

Rotarod performance was tested on days 1, 8, 22, and 33. On day 1st, there was no statistically significant difference in motor coordination across the treatment groups, indicating a progressive baseline. On day 8th, EBRO therapy significantly decreased fall-off time, indicating enhanced motor function. On day 22nd, EBRO + PNN50 and EBRO + PNN100 therapies significantly increased fall-off time compared with the EBRO group, indicating that motor coordination improved dose-dependently. EBRO + VB12 (30) co-administration slightly improved the fall-off time. On day 33, the EBRO + PNN100 group demonstrated superior motor function and longer fall-off time than the EBRO + PNN50 group, indicating a dose-dependent therapeutic effect of PNN. The combination of EBRO + PNN100 and EBRO + VB12 (30) enhanced PNN monotherapy demonstrates synergistic potential and neuroprotective benefits. The analysis of the fall of time involved a two-way ANOVA, which produced an F-statistic of F (7,56) = 572.8 (p < 0.01), Figure 8B.

#### PNN reduces the number of foot slips

On days 1st, 8th, 15th, 22nd, 29th, and 35th, the Beam Crossing Task was used to assess neuromuscular coordination, foot slips, and gait abnormalities. A uniform baseline was established on day 1, with no significant differences between the experimental groups. Compared to the Sham Control, Vehicle Control, and PNN Perse groups, the EBRO group showed a significantly higher rate of foot slips by day 8th, suggesting early motor impairment. Compared with the EBRO group, the number of foot slips on days 22nd, 29th, and 35th was significantly reduced by long-term treatment with EBRO + PNN50 and EBRO + PNN100. Significantly, the group receiving EBRO + PNN100 on day 35th demonstrated better neuromuscular coordination and a greater reduction in foot slip number than the group receiving the lower-dose EBRO + PNN50, indicating a dose-dependent therapeutic effect. Furthermore, EBRO + VB12 (30) treatment additionally significantly decreased the number of foot slips. EBRO + PNN100 + EBRO + VB12 (30) produced the greatest improvement over all assessment days. The neuroprotective effectiveness of co-administration is further supported by this combination therapy’s superior effect, suggesting a possible synergistic benefit compared with monotherapy. The analysis of the number of foot slips involved a two-way ANOVA, which produced an F-statistic of F (7,56) = 2,600, (p < 0.01), Figure 8C.

#### PNN reduces the escape latency time (ELT)

The Morris Water Maze test was used to measure the experimental rats’ Escape Latency Time (ELT) on days 31st^,^ 32nd, 33rd^,^ and 34th to evaluate their memory and cognitive function. Compared to the Sham Control, Vehicle Control, and PNN Perse groups, the EBRO group showed a significant decrease in ELT, suggesting compromised spatial learning and memory. Conversely, compared with the EBRO group, long-term PNN treatment at EBRO + PNN50 and EBRO + PNN100 doses led to a progressive decline in ELT on days 31st and 32nd, indicating enhanced cognitive function. On days 33 and 34, the EBRO + PNN100 group showed a more pronounced decrease in ELT than the EBRO + PNN50 group, indicating a dose-dependent improvement in long-term memory and learning capacity. Moreover, combination therapy with EBRO + PNN100 and EBRO + VB12 (30) was more effective than PNN alone. The analysis of the escape latency time involved a two-way ANOVA, which produced an F-statistic of F (7,56) = 2,612, (p < 0.01) Figure 8D.

#### PNN increases the time spent in the target quadrant (TSTQ)

Memory consolidation in rats was assessed on day 35 of the experiment by measuring the time spent in the target quadrant. Compared to the Sham Control, Vehicle Control, and PNN Perse groups, the EBRO group spent a lot less time in the target quadrant. Oral administration in the EBRO + PNN50 and EBRO + PNN100 groups increased the time spent in the target quadrant compared with the EBRO group. Notably, the PNN100 group showed a significantly greater improvement than the PNN50 group, suggesting a dose-dependent effect of PNN on memory consolidation. EBRO + VB12 (30) was administered to the PNN50 group, both as a stand-alone treatment and in combination with high-dose PNN100, to further evaluate the efficacy of the treatment regimen. The PNN100 + VB12 (30) group spent significantly more time in the target quadrant than the PNN monotherapy groups, indicating enhanced memory consolidation with the combination treatment. The analysis of the time spent quadrant involved a one-way ANOVA, which produced an F-statistic of F (7,49) = 392.3, (p < 0.01), Figure 8E.

#### PNN restores body weight changes

Weekly body weight measurements were made during the trial to assess PNN effectiveness in EBRO-induced rats. Initially, there were no significant differences in body weight among the groups. Compared with the Sham Control group, Vehicle Control group, and PNN Perse group, the EBRO-induced group showed a progressive decrease in body weight on days 8th, 15th, and 22nd. In comparison to the EBRO-induced group, the EBRO + PNN 50 and EBRO + PNN 100 groups showed a discernible and dose-dependent increase in body weight by the 29th and 35th days. In comparison to the EBRO-induced group, the EBRO + VB12 (30) group also demonstrated a notable improvement in body weight over time. Significantly, the EBRO + PNN100 group regained body weight more effectively than the EBRO + PNN50 group by day 35, suggesting a dose-dependent therapeutic effect. Furthermore, compared with the PNN monotherapy groups, the combination groups EBRO + PNN 100 and EBRO + VB12 (30) showed a significantly greater improvement in body weight recovery, indicating a synergistic effect of the combination therapy. The analysis of the rat body weight involved a two-way ANOVA, which produced an F-statistic of F (7,56) = 3,352, (p < 0.01), Figure 8F.

#### PNN improves the brain-to-body weight ratio

The neuroprotective effects of PNN in rats with experimentally induced multiple sclerosis were assessed on day 36th by measuring the relative brain-to-body weight ratio. Compared to the EBRO-induced group, the Sham Control, Vehicle Control, and PNN Perse groups exhibited significantly higher brain-to-body weight ratios, suggesting preservation of neural integrity. Compared with the EBRO group, a dose-dependent, significant increase in the brain-to-body weight ratio was observed in the EBRO + PNN50 and EBRO + PNN100 groups, indicating a neuroprotective effect of PNN. Comparing the VB12 (30) group to the EBRO-induced group, the first group demonstrated a notable improvement in this parameter. Among all treatment groups, the combination group EBRO + PNN100 and EBRO + VB12 (30) showed the greatest restoration of the brain-to-body weight ratio, indicating a synergistic effect of combination therapy in boosting neuroprotection. The analysis of the brain-to-body rat ratio involved a one-way ANOVA, which produced an F-statistic of F (7,49) = 2,561, (p < 0.01), Figure 8G.

### PNN neuroprotective role in mitigating EBRO-induced neurochemical alterations in MS rat model

#### PNN modulates GDNF-GFRA1- AKT-ERK1/2- GSK-3Beta levels

**FIGURE 9:**
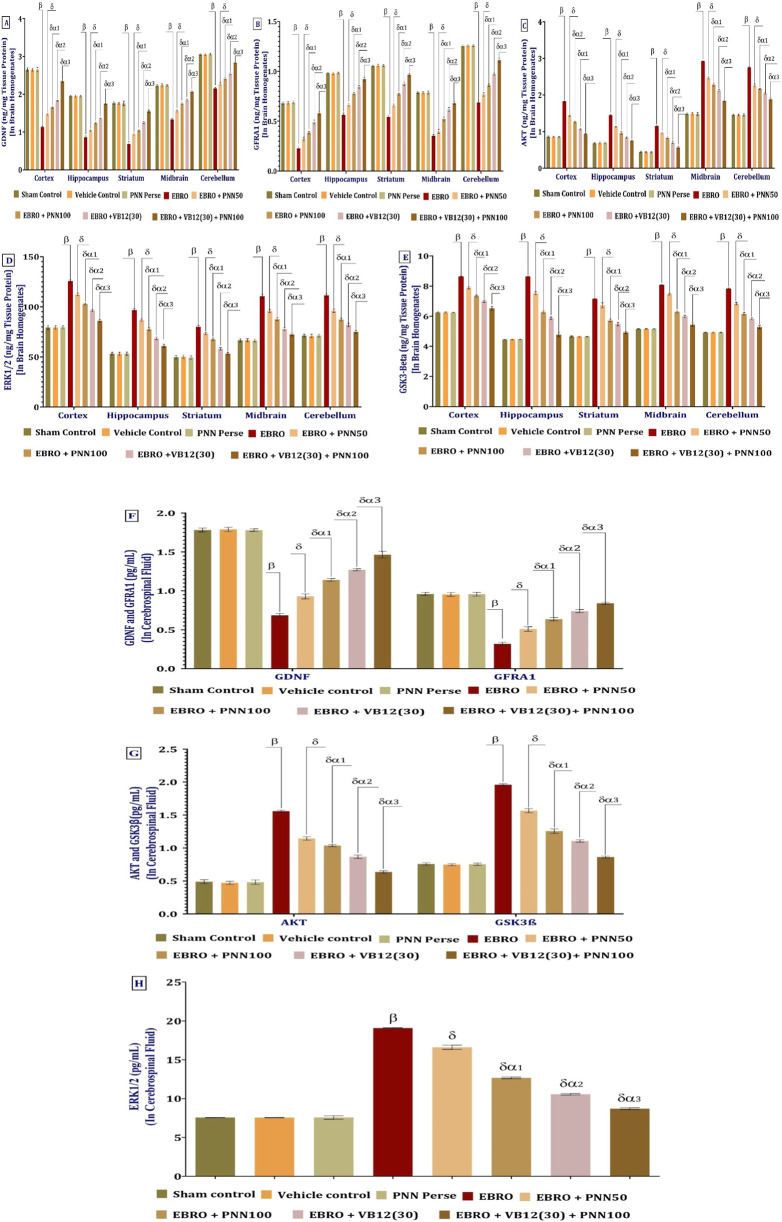
**(A–H)** PNN neuroprotective role in mitigating EBRO-induced alterations in levels of cellular and molecular targets in MS rat model: GDNF **(A)**, GFRA1 **(B)**, AKT **(C)**, ERK1/2 **(D)**, GSK3-Beta **(E)**, in brain homogenates, and GDNF, GFRA1, AKT, ERK1/2, GSK3-Beta in CSF levels **(F–H)**. Statistical analysis was performed using a one-way ANOVA followed by Tukey’s *post hoc* test to determine significant differences among groups **(A–H)**. Data were presented as mean ± standard deviation (SD), with statistical significance set at *p* < 0.01. Each experimental group consisted of eight wistar rats (n = 8). β v/s Sham Control, Vehicle Control, and PNN Perse; δ v/s EBRO; δα1 v/s EBRO + PNN50; δα2 v/s EBRO + PNN100, EBRO + PNN50; and δα3 v/s EBRO + VB12 (30), EBRO + PNN100, EBRO + PNN50. To identify significant differences between groups, a one-way ANOVA and Tukey’s *post hoc* test were used for statistical analysis **(A–H)**. The statistical significance level was set at p < 0.01, and the data were displayed as mean ± standard deviation (SD). There were eight wistar rats (n = 8) in each experimental group. β v/s Sham Control, Vehicle Control, and PNN Perse; δ v/s EBRO; δα1 v/s EBRO + PNN50; δα2 v/s EBRO + PNN100, EBRO + PNN50; and δα3 v/s EBRO + VB12 (30), EBRO + PNN100, EBRO + PNN50.

The ELISA method was used to measure GDNF protein levels in rat brain homogenates from the cerebral cortex, hippocampus, striatum, midbrain, and cerebellum, as per the experimental protocol. Compared with the Sham Control, Vehicle Control, and PNN Perse groups, the EBRO-induced group exhibited significant downregulation of GDNF levels across all brain regions examined, indicating neurodegeneration and impaired neurotrophic support. In rats with EBRO + PNN100 and EBRO + PNN50 administration, a dose-dependent increase in GDNF levels was observed. GDNF levels increased significantly in the PNN100 group compared to the PNN50 group, indicating that the higher dose had a greater neuroprotective effect. To assess therapeutic enhancement, PNN50 and VB12 (30) were administered as monotherapies and in combination in the high-dose group (PNN100 + VB12 (30). Remarkably, the combination group outperformed all other treatment groups in upregulating GDNF, effectively reduced the EBRO-induced suppression. These results underscore the potential neurotrophic and neuroprotective roles of PNN, demonstrating its ability to restore GDNF levels across key brain regions in MS, particularly when combined with VB12 (30). The analysis of the GDNF involved a one-way ANOVA, which produced an F-statistic of cerebral cortex F (7,49) = 3,000, p < 0.01, hippocampus F (7,49) = 2,810, p < 0.01, Striatum F (7,49) = 827.4, p < 0.01, midbrain F (7,49) = 1,157, p < 0.01, F (7,49) = 1,459, (p < 0.01), Figure 9A.

The ELISA method was used to measure GFRA1 protein levels in rat brain homogenates from the cerebral cortex, hippocampus, striatum, midbrain, and cerebellum, as per the experimental protocol. Compared to the Sham Control, Vehicle Control, and PNN Perse group, the EBRO group, which simulates multiple sclerosis, showed a significant downregulation of GFRA1 levels across all studied brain regions, suggesting neurodegeneration and impaired neurotrophic signaling. In rats treated with EBRO + PNN50 and EBRO + PNN100, a dose-dependent upregulation of GFRA1 levels was observed. The significantly greater increase in GFRA1 expression observed in the PNN100 group compared to the PNN50 group indicates a stronger neuroprotective effect at the higher dose. To assess potential therapeutic enhancement, VB12 (30) was administered at the PNN50 dose both alone and in combination with high-dose PNN100 + VB12 (30). Remarkably, the combination group exhibited a significant increase in GFRA1 levels, surpassing all other treatment groups and effectively moderating the EBRO-induced suppression. These results demonstrate that PNN can restore GFRA1 expression in key brain regions affected by experimental multiple sclerosis, highlighting its neurotrophic and neuroprotective potential, particularly when combined with VB12 (30). The analysis of the GFRA1 involved a one-way ANOVA, which produced an F-statistic of cerebral cortex F (7,49) = 773.1, (p < 0.01), hippocampus F (7,49) = 1,065, (p < 0.01), striatum F (7,49) = (1,698), p < 0.01, midbrain F (7,49) = 1,010, p < 0.01, cerebellum F (7,49) = 2,158, (p < 0.01), Figure 9B.

The ELISA method was used to measure AKT protein levels in rat brain homogenates from the cerebral cortex, hippocampus, striatum, midbrain, and cerebellum, as described in the experimental protocol. Compared with the Sham Control, Vehicle Control, and PNN Perse groups, the EBRO-induced group showed a marked increase in AKT levels after 7 days of EBRO exposure. This suggests increased activation of AKT signaling associated with neuroinflammation or cellular stress. Compared with the EBRO-induced group, chronic administration of PNN resulted in a significant decrease in AKT levels. The PNN 100 group exhibited a noticeably higher reduction in AKT expression than the PNN50 group, demonstrating that this effect was dose-dependent. Additionally, the combination group VB12 (30) + PNN 100 showed the greatest decrease in AKT levels, exceeding those of both the PNN monotherapy and VB12 (30) monotherapy groups. These results suggest that PNN and VB12 (30) act synergistically to downregulate AKT signaling, thereby enhancing their neuroprotective efficacy against EBRO-induced neurotoxicity. The analysis of the AKT involved a one-way ANOVA, which produced an F-statistic of cerebral cortex [F (7,49) = 3,868, p < 0.01], hippocampus F (7,49) = 1,128, (p < 0.01), striatum F (7,49) = 1,286, (p < 0.01), midbrain F (7,49) = 2,294, (p < 0.01), cerebellum F (7,49) = 3,364, (p < 0.01), Figure 9C.

The ELISA method was used to measure ERK1/2 protein levels in rat brain homogenates from the cerebral cortex, hippocampus, striatum, midbrain, and cerebellum, as described in the experimental protocol. Compared to the Sham Control, Vehicle Control, and PNN Perse groups, the EBRO-induced group model of multiple sclerosis exhibited a significant upregulation of ERK1/2 expression, suggesting activation of inflammatory or stress-related signaling pathways. ERK1/2 levels decreased in a dose-dependent manner following continuous administration of PNN50 and PNN100. The reduction was more pronounced in the PNN100 group than in the PNN50 group, indicating a stronger anti-inflammatory or neuroprotective effect at the higher dose. To evaluate potential therapeutic enhancement, VB12 (30) was given at a dose of 50 to both the VB12 (30) group and the PNN100 + VB12 (30) + PNN100 group. Significantly, the Combination group outperformed all other treatment groups and successfully mitigated the EBRO-induced elevation in ERK1/2 levels. According to these findings, PNN may have strong regulatory effects on ERK1/2 signaling pathways, especially when paired with VB12 (30), which could enhance its neuroprotective potential in an experimental model of multiple sclerosis. The analysis of the ERK1/2 involved a one-way ANOVA, which produced an F-statistic of cerebral cortex [F (7,49) = 1,081, p < 0.01], hippocampus F (7,49) = 1,148, (p < 0.01), striatum F (7,49) = 493.9, (p < 0.01), midbrain F (7,49) = 798.5, (p < 0.01), cerebellum F (7,49) = 723.9, (p < 0.01), Figure 9D.

The ELISA method was used to measure GSK3β protein levels in rat brain homogenates from the cerebral cortex, hippocampus, striatum, midbrain, and cerebellum, according to the experimental protocol. Comparing the EBRO-induced group to the Sham Control, Vehicle Control, and PNN Perse groups, a significant increase in GSK3-β expression was seen, indicating increased neuroinflammatory and apoptotic signaling typical of an experimental model of multiple sclerosis. GSK3-β levels decreased significantly following PNN treatment, with a dose-dependent effect. GSK3-β levels were significantly lower in the PNN100 group than in the PNN50, suggesting a stronger neuroprotective effect at the higher dose. Additionally, GSK3-β levels decreased more markedly in the combination group PNN 100 + VB12 (30) than in either monotherapy. The finding highlights the therapeutic potential of PNN100 and VB12 (30) in reducing EBRO-induced neurotoxicity and indicates a synergistic effect of these two compounds in regulating GSK3-β expression. The analysis of the GSK3-Beta involved a one-way ANOVA, which produced an F-statistic of cerebral cortex F (7,49) = 1959, (p < 0.01), hippocampus F (7,49) = 3,973, (p < 0.01), striatum F (7,49) = 1,084, (p < 0.01), midbrain (p < 0.01), cerebellum F (7,49) = 1958, (p < 0.01), Figure 9E.

In the cerebrospinal fluid (CSF) of rats with experimental multiple sclerosis (MS), PNN affects the levels of GDNF, GFRA1, AKT, ERK1/2, and GSK3-β. CSF samples were collected and analyzed to assess the levels of these molecular markers after the experimental procedure was finished. AKT, ERK1/2, and GSK3-β levels were significantly higher in the EBRO-induced group compared to the Sham Control, Vehicle Control, and PNN Perse groups, whereas GDNF and GFRA1 levels were significantly lower. These findings suggest dysregulated neurotrophic support and increased inflammatory signaling. PNN was more successful than PNN50 at achieving baseline levels of these markers when administered chronically, especially at 100 mg/kg (PNN100). Treatment with VB12 (30) alone significantly normalized CSF levels of GDNF, GFRA1, AKT, ERK1/2, and GSK3-β, with even greater normalization observed when combined with PNN100, indicating a synergistic neuroprotective effect. These results demonstrate the potential of PNN100 and VB12 (30), particularly in combination, to modulate key molecular pathways implicated in inflammation and neurodegeneration in an experimental model of multiple sclerosis. The analysis of the CSF involved a one-way ANOVA, which produced an F-statistic of GDNF- F (7,49) = 2044, (p < 0.01), GFRA1 F (7,49) = 952.4, (p < 0.01), AKT F (7,49) = 1808, (p < 0.01), ERK1/2 F (7,49) = 7,166, (p < 0.01), GSK3-Beta F (7,49) = 3,494, (p < 0.01) Figures 9F–H.

#### PNN modulates apoptotic markers

**FIGURE 10:**
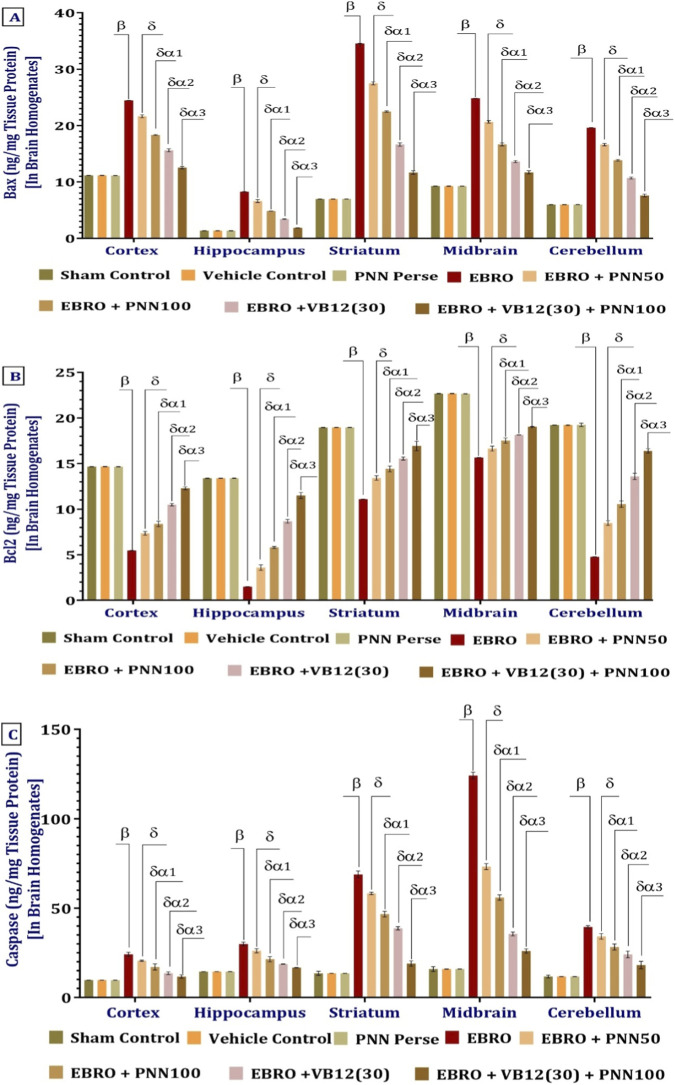
**(A–C)** PNN neuroprotective role in mitigating EBRO-induced alterations in levels of apoptotic markers like Bax **(A)**, Bcl-2 **(B)**, and Caspase-3 **(C)** in brain homogenates. To identify significant differences between groups, a one-way ANOVA and Tukey’s *post hoc* test were used for statistical analysis **(A–C)**. The statistical significance level was set at p < 0.01, and the data were displayed as mean ± standard deviation (SD). There were eight wistar rats (n = 8) in each experimental group. β v/s Sham Control, Vehicle Control, and PNN Perse; δ v/s EBRO; δα1 v/s EBRO + PNN50; δα2 v/s EBRO + PNN100, EBRO + PNN50; and δα3 v/s EBRO + VB12 (30), EBRO + PNN100, EBRO + PNN50.

To assess the degree of neurodegeneration, apoptotic markers, including Bax, Bcl-2, and Caspase-3, were measured in brain tissue homogenates from different regions of rats with experimental multiple sclerosis (MS). Compared with the Sham Control, Vehicle Control, and PNN Perse groups, the EBRO group showed a significant upregulation of Bax expression, suggesting increased pro-apoptotic activity. Bax levels decreased following treatment with PNNf50 and PNN100, with a more pronounced reduction in the PNN100 group than in the PNN50 group. Bax expression was also significantly reduced by VB12 (30) treatment, whether administered alone or in combination with PNN100. These findings suggest that both PNN100 and VB12 (30) possess anti-apoptotic properties, with combination therapy offering enhanced neuroprotection through more effective inhibition of Bax-mediated apoptotic signaling in EBRO-induced neurotoxicity. The statistical significance of differences is evaluated using a one-way analysis of variance (ANOVA) on brain homogenates from different brain regions, such as the cortex. The analysis of the bax involved a one-way ANOVA, which produced an F-statistic of cerebral cortex F (7,49) = 10,132, (p < 0.01), hippocampus F (7,49) = 5,727, p < 0.01], striatum F (7,49) = 29,851, (p < 0.01), midbrain F (7,49) = 8,802, (p < 0.01), cerebellum F (7,49) = 10,963, (p < 0.01), Figure 10A.

The EBRO-induced group exhibited elevated levels of the anti-apoptotic marker Bcl-2 compared to the Sham Control, Vehicle Control, and PNN Perse groups. Given the increased neurodegeneration in the experimental MS model, this elevation may represent a compensatory response. The dose-dependent normalization of Bcl-2 levels following chronic administration of PNN50 and PNN100 suggests a potential regulatory effect on apoptotic pathways. Interestingly, Bcl-2 levels were lower in the VB12 (30) monotherapy group than in the EBRO group, suggesting limited efficacy of VB12 (30) alone in restoring Bcl-2 expression. Notably, the combination therapy of VB12 (30) and PNN100 significantly upregulated Bcl-2 levels, exceeding the effects observed with either treatment alone. This research suggests that PNN100 and VB12 (30) act synergistically, with combined administration potentially enhancing neuroprotective effects and increasing anti-apoptotic activity in EBRO-induced neurodegeneration. The analysis of the bcl-2 involved a one-way ANOVA, which produced an F-statistic of cerebral cortex F (7,49) = 4,433, (p < 0.01), hippocampus F (7,49) = 5,356, (p < 0.01), striatum F (7,49) = 1,116, (p < 0.01), midbrain F (7,49) = 3,870, (p < 0.01), cerebellum F (7,49) = 4,512, (p < 0.01), Figure 10B.

Compared with the Sham Control, Vehicle Control, and PNN Perse groups, brain homogenates from rats exposed to EBRO for 7 days showed a significant increase in Caspase-3 levels, a key pro-apoptotic marker, indicating increased apoptotic activity. PNN50 and PNN100 produced a dose-dependent reduction in Caspase-3 levels. The reduction in the PNN100 group was significantly greater than that observed in the PNN50 group. Moreover, monotherapy with VB12 (30) reduced Caspase-3 expression. Combining EBRO with VB12 (30) or PNN100 significantly reduced Caspase-3 levels, indicating a synergistic anti-apoptotic effect that may enhance neuroprotection against EBRO-induced neurotoxicity. The analysis of the caspase-3 involved a one-way ANOVA, which produced an F-statistic of cerebral cortex F (7,49) = 302.4, (p < 0.01), hippocampus F (7,49) = 459.0, (p < 0.01), striatum F (7,49) = 2,814, (p < 0.01), midbrain F (7,49) = 8,087, (p < 0.01), cerebellum F (7,49) = 472.4, (p < 0.01), Figure 10C.

#### PNN in alleviating the inflammatory cytokines

**FIGURE 11:**
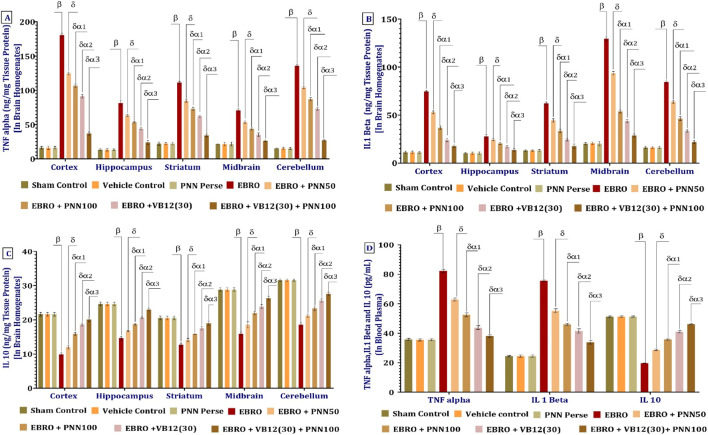
**(A–D)** PNN neuroprotective role in mitigating EBRO-induced alterations in levels of pro-inflammatory cytokines like TNF-α **(A)**, IL-1β **(B)**, IL-10 **(C)** in brain homogenates and TNF-α, IL-1β, and IL-10 in blood plasma **(D)**. To identify significant differences between groups, a one-way ANOVA and Tukey’s *post hoc* test were used for statistical analysis **(A–D)**. The statistical significance level was set at p < 0.01, and the data were displayed as mean ± standard deviation (SD). There were eight wistar rats (n = 8) in each experimental group. β v/s Sham Control, Vehicle Control, and PNN Perse; δ v/s EBRO; δα1 v/s EBRO + PNN50; δα2 v/s EBRO + PNN100, EBRO + PNN50; and δα3 v/s EBRO + VB12 (30), EBRO + PNN100, EBRO + PNN50.

To evaluate the neuroprotective potential of PNN, interleukin-1β (IL-1β) levels were measured in various regions of the rats’ brains. Compared to the Sham Control, Vehicle Control, and PNN Perse groups, the EBRO group exhibited a pronounced neuroinflammatory response, evidenced by a significant increase in IL-1β expression. Extended administration of PNN50 significantly decreased IL-1β levels. The PNN100 group demonstrated greater efficacy than PNN50 in reducing neuroinflammation, indicating a dose-dependent anti-inflammatory effect. Additionally, VB12 (30), a commonly used medication, significantly reduced IL-1β levels. Combining VB12 (30) with PNN100 produced the greatest reduction, indicating a synergistic anti-inflammatory effect and supporting its therapeutic potential in EBRO-induced neuroinflammation. The analysis of the IL-1β involved a one-way ANOVA, which produced an F-statistic of cerebral cortex F (7,49) = 2,121, p < 0.01], hippocampus F (7,49) = 189.3, (p < 0.01), striatum F (7,49) = 1,337, p < 0.01), midbrain F 7,49) = 4,362, (p < 0.01), cerebellum F (7,49) = 3,079, (p < 0.01), Figure 11A.

To evaluate the neuroprotective effects of PNN, tumor necrosis factor-alpha TNF-α levels were measured in various regions of the rats’ brains. TNF-α levels were significantly elevated in rats exposed to EBRO for 7 days compared to the Sham Control, Vehicle Control, and PNN Perse groups, indicating increased neuroinflammatory activity. Long-term PNN treatment reduced TNF-α levels in a dose-dependent manner, with the PNN100 group demonstrating greater anti-inflammatory efficacy than the PNN50 group. Additionally, VB12 (30) treatment significantly reduced TNF-α levels both alone and in combination with PNN100, indicating a synergistic anti-inflammatory effect that may enhance neuroprotection in EBRO-induced neuroinflammation. The analysis of the TNF-α involved a one-way ANOVA, which produced an F-statistic of cerebral cortex F (7,49) = 5,190, (p < 0.01), hippocampus F (7,49) = 1,372, (p < 0.01), striatum F (7,49) = 2,477, (p < 0.01), midbrain F (7,49) = 6,146, (p < 0.01), cerebellum F (7,49) = 6,825, (p < 0.01), Figure 11B.

To assess the neuroprotective potential of PNN, interleukin-10 (IL-10) levels were measured in various brain regions of rats. The EBRO group showed a significant increase in IL-10 levels compared to the Sham Control, Vehicle Control, and PNN Perse groups, indicating a compensatory anti-inflammatory response following EBRO exposure. Chronic administration of PNN resulted in a significant, dose-dependent decrease in IL-10 expression, with the PNN100 group showing greater efficacy than the PNN50 group in reducing IL-10 levels. Additionally, VB12 (30) treatment, alone or in combination with PNN100, significantly reduced IL-10 levels. Combining therapies produced the greatest decrease, indicating a synergistic anti-inflammatory effect and underscoring their potential as a treatment for EBRO-induced neuroinflammation. The analysis of the IL-10 involved a one-way ANOVA, which produced an F-statistic of cerebral cortex F (7,49) = 603.1, (p < 0.01), hippocampus F (7,49) = 711.0, (p < 0.01), striatum F (7,49) = 318.7, (p < 0.01), midbrain F (7,49) = 548.8, (p < 0.01), cerebellum F (7,49) = 926.0, (p < 0.01), Figure 11C.

The neuroprotective effects of PNN were evaluated by measuring the levels of pro-inflammatory cytokines TNF-α, IL-1β, and IL-10 in the blood plasma of rats in an experimental model of MS. Rats exposed to EBRO showed a significant increase in these cytokine levels compared to the Sham Control, Vehicle Control, and PNN Perse groups, indicating an enhanced systemic inflammatory response. Prolonged administration of PNN resulted in a significant reduction of TNF-α, IL-1β, and IL-10 levels in plasma. Notably, treatment with PNN100 was significantly more effective than PNN50, confirming a dose-dependent anti-inflammatory effect. Additionally, the standard medication VB12 (30) significantly reduced pro-inflammatory cytokine levels, both when used alone and in combination with PNN100. In EBRO-induced neuroinflammation, combination therapy produced the most significant reduction, indicating a synergistic effect that enhances systemic anti-inflammatory and neuroprotective outcomes. The analysis of the blood plasma involved a one-way ANOVA, which produced an F-statistic of IL-10 F (7,49) = 3,751, (p < 0.01), IL-1Beta F (7,49) = 2,450, (p < 0.01), and TNF-alpha F (7,49) = 2,151, (p < 0.01), Figure 11D.

#### PNN ameliorates MBP and NEFL levels

**FIGURE 12:**
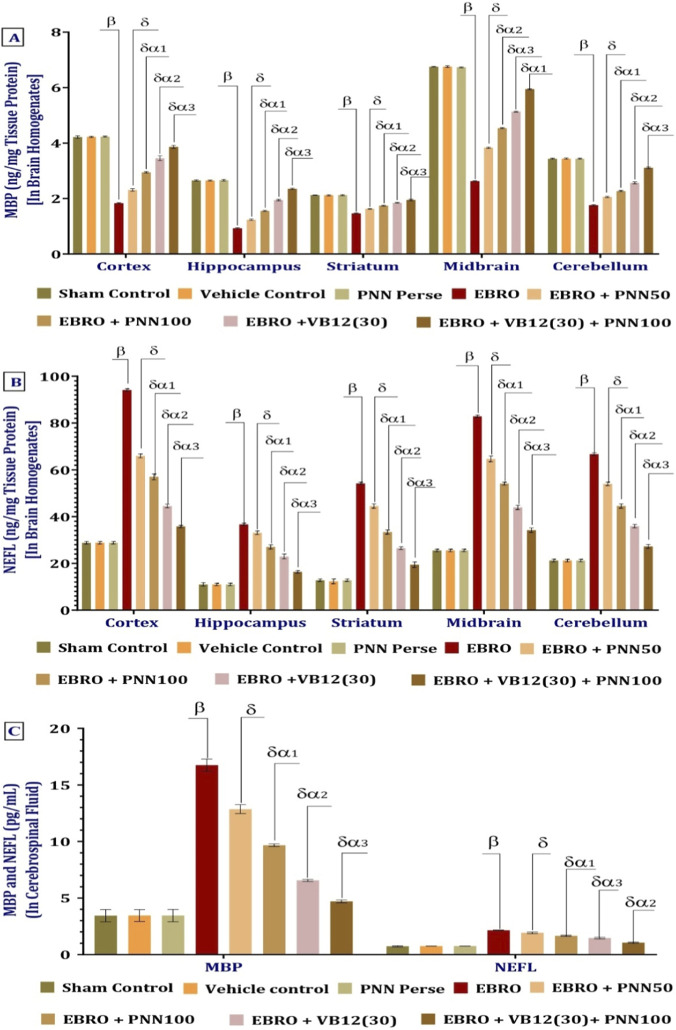
**(A–C)** PNN neuroprotective role in mitigating EBRO-induced alterations in MBP **(A)**, NEFL **(B)** in brain homogenates, and MBP and NEFL levels in CSF **(C)** in MS rat model. To identify significant differences between groups, a one-way ANOVA and Tukey’s *post hoc* test were used for statistical analysis **(A–C)**. The statistical significance level was set at p < 0.01, and the data were displayed as mean ± standard deviation (SD). There were eight wistar rats (n = 8) in each experimental group. β v/s Sham Control, Vehicle Control, and PNN Perse; δ v/s EBRO; δα1 v/s EBRO + PNN50; δα2 v/s EBRO + PNN100, EBRO + PNN50; and δα3 v/s EBRO + VB12 (30), EBRO + PNN100, EBRO + PNN50.

ELISA was used at the end of the experiment to measure myelin basic protein (MBP) levels in brain homogenates. EBRO-induced rats showed significantly lower MBP levels than the Sham Control, Vehicle Control, and PNN Perse groups, suggesting demyelination linked to neurodegeneration. Chronic administration of PNN50 and PNN100 resulted in a dose-dependent increase in MBP levels, with a more pronounced restoration observed in the PNN100 group. Similarly, MBP levels were significantly elevated following VB12 (30) treatment compared to the EBRO group. Among all treatment groups, the combination therapy of VB12 (30) and PNN100 significantly increased MBP expression, surpassing the effects of PNN monotherapy. These results suggest that combination therapy exerts synergistic remyelinating and neuroprotective effects in EBRO-induced demyelination. These findings suggest that PNN increases MBP levels in various brain regions affected by EBRO induction in a dose-dependent manner. Furthermore, PNN100 and VB12 (30) showed a synergistic effect, leading to a more pronounced restoration of MBP levels than monotherapy. The analysis of the MBP involved a one-way ANOVA, which produced an F-statistic of cerebral cortex [F (7,49) = 3,588, p < 0.01], hippocampus F (7,49) = 7,094, (p < 0.01), striatum F (7,49) = 1,396, (p < 0.01), midbrain F (7,49) = 43,982, (p < 0.01), cerebellum F (7,49) = 5,382, (p < 0.01), Figure 12A.

NEFL protein levels in brain homogenates were measured using the Enzyme-Linked Immunosorbent Assay (ELISA) method. Rats treated with EBRO exhibited significantly higher NEFL levels compared to the Sham Control, Vehicle Control, and PNN Perse groups, suggesting axonal damage. Extended administration of PNN50 and PNN100 significantly reduced NEFL levels in a dose-dependent manner compared to the EBRO-induced group, with PNN100 proving more effective than VB12 (30) monotherapy. These results suggest that PNN reduces NEFL protein levels in various brain regions affected by EBRO induction in a dose-dependent manner. The analysis of the NEFL involved a one-way ANOVA, which produced an F-statistic of cerebral cortex F (7,49) = 8,660, (p < 0.01), hippocampus F (7,49) = 1,557, (p < 0.01), striatum F (7,49) = 3,631, (p < 0.01), midbrain F (7,49) = 6,830, (p < 0.01), cerebellum F (7,49) = 5,267, (p < 0.01), Figure 12B.

To evaluate the neuroprotective effects of PNN, cerebrospinal fluid (CSF) samples were collected from EBRO-treated rats, and ELISA was used to measure levels of myelin basic protein (MBP) and neurofilament light chain (NEFL). Rats exposed to EBRO exhibited significantly higher CSF levels of MBP and NEFL compared to the Sham Control, Vehicle Control, and PNN Perse groups. Treatment with PNN50 and PNN100 significantly lowered CSF levels of MBP and NEFL, with PNN100 having a stronger effect. Similarly, VB12 (30) treatment also reduced MBP and NEFL levels compared to the EBRO-induced group, supporting its neuroprotective role. These results demonstrate the neuroprotective potential of PNN treatment, as it effectively reduces MBP and NEFL levels in the cerebrospinal fluid of rats with EBRO-induced multiple sclerosis, particularly at the higher PNN100 dose and when combined with VB12 (30). The analysis of the CSF involved a one-way ANOVA, which produced an F-statistic for MBP: F (7,49) = 1,152 (p < 0.01); for NEF: F (7,49) = 986.8 (p < 0.01); see Figure 12C.

#### PNN ameliorates neurotransmitter levels

**FIGURE 13:**
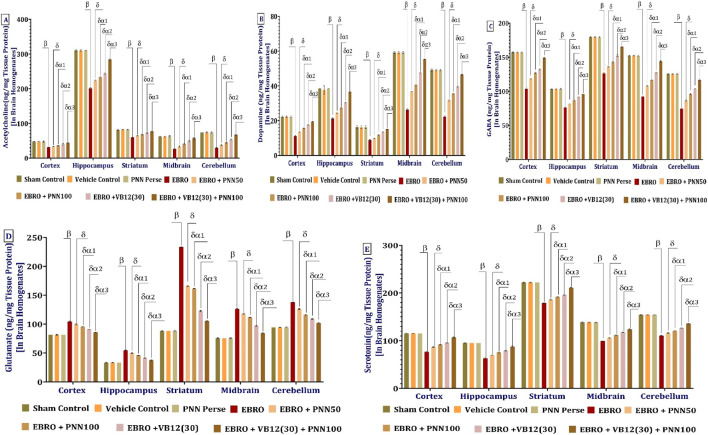
**(A–E)** PNN neuroprotective role in mitigating EBRO-induced alterations in neurotransmitter levels, including Acetylcholine **(A)**; Dopamine **(B)**; GABA **(C)**; Glutamate **(D)**, and Serotonin **(E)** in brain homogenates. To identify significant differences between groups, a one-way ANOVA and Tukey’s *post hoc* test were used for statistical analysis **(A–D)**. The statistical significance level was set at p < 0.01, and the data were displayed as mean ± standard deviation (SD). There were eight wistar rats (n = 8) in each experimental group. β v/s Sham Control, Vehicle Control, and PNN Perse; δ v/s EBRO; δα1 v/s EBRO + PNN50; δα2 v/s EBRO + PNN100, EBRO + PNN50; and δα3 v/s EBRO + VB12 (30), EBRO + PNN100, EBRO + PNN50.

Levels of acetylcholine, dopamine, GABA, glutamate, and serotonin were measured at the end of the experimental protocol. Rats exposed to EBRO exhibited significantly lower levels of acetylcholine, dopamine, GABA, and serotonin compared to the Sham Control, Vehicle Control, and PNN Perse groups. Conversely, glutamate levels were significantly elevated after 7 days of EBRO exposure. Neurotransmitter levels were restored by chronic administration of PNN50 and PNN100, with PNN100 demonstrating greater efficacy. Additionally, VB12 (30) treatment alone increased levels of acetylcholine, dopamine, GABA, and serotonin, while reducing glutamate levels. The most pronounced normalization of neurotransmitter levels occurred with the combination of VB12 (30) and high-dose PNN, indicating a synergistic effect on neurochemical restoration. The analysis of the acetylcholine involved a one-way ANOVA, which produced an F-statistic of cerebral cortex F (7,49) = 288.1, (p < 0.01), hippocampus: F (7,49) = 5,599, (p < 0.01), striatum: F (7,49) = 387.8, (p < 0.01), midbrain: F (7,49) = 8,104, (p < 0.01), cerebellum F (7,49) = 1,264, (p < 0.01), Figure 13A. Dopamine: cerebral cortex: F (7,49) = 899.4, (p < 0.01), hippocampus: F (7,49) = 6,183, (p < 0.01), striatum: F (7,49) = 249.8, (p < 0.01), midbrain: F (7,49) = 7,246, p < 0.01], cerebellum: F (7,49) = 3,424, (p < 0.01), Figure 13B. GABA: cerebral cortex: F (7,49) = 5,130, (p < 0.01), hippocampus: F (7,49) = 2,165, (p < 0.01), striatum: F (7,49) = 4,906, (p < 0.01), midbrain: F (7,49) = 7,709, (p < 0.01), cerebellum: F (7,49) = 5,022, (p < 0.01), Figure 13C. Glutamate: cerebral cortex: F (7,49) = 2089, (p < 0.01), hippocampus: F (7,49) = 1926, (p < 0.01), striatum: F (7,49) = 35,141, (p < 0.01), midbrain: F (7,49) = 5,320, (p < 0.01), cerebellum: F (7,49) = 4,643, p < 0.01), Figure 13D. Serotonin: cerebral cortex: F (7,49) = 4,859, (p < 0.01), hippocampus: F (7,49) = 2,902, p < 0.01], striatum: F (7,49) = 6,902, (p < 0.01), midbrain: F (7,49) = 2,842, (p < 0.01), cerebellum: F (7,49) = 6,760, (p < 0.01), Figure 13E.

#### PNN modulates CBC levels

**FIGURE 14:**
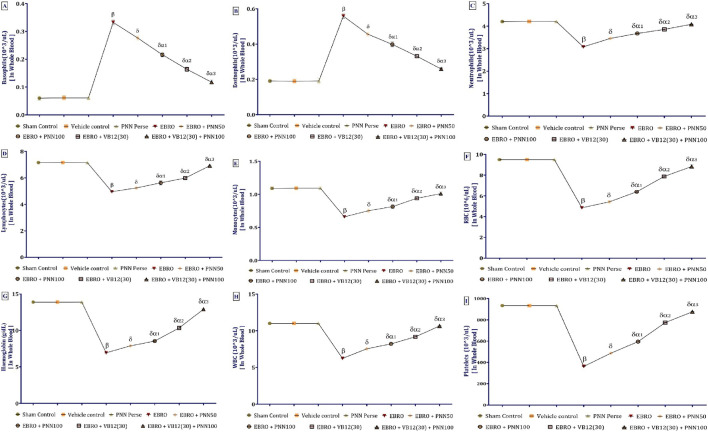
**(A–I)** PNN neuroprotective role in mitigating EBRO-induced alterations in levels of Basophils **(A)**, Eosinophils **(B)**, Neutrophils **(C)**, Lymphocytes **(D)**, Monocytes **(E)**, RBC **(F)**, Haemoglobin **(G)**, WBC **(H)** and Platelets **(I)**. Statistical analysis was performed using a one-way ANOVA followed by Tukey’s *post hoc* test to determine significant differences among groups **(A–I)**. Data were presented as mean ± standard deviation (SD), with statistical significance set at *p* < 0.01. Each experimental group consisted of eight wistar rats (n = 8). β v/s Sham Control, Vehicle Control, and PNN Perse; δ v/s EBRO; δα1 v/s EBRO + PNN50; δα2 v/s EBRO + PNN100, EBRO + PNN50; and δα3 v/s EBRO + VB12 (30), EBRO + PNN100, EBRO + PNN50.

Blood samples were collected from the experimental rats at the end of the trial for complete blood count (CBC) analysis. Red blood cells (RBC), white blood cells (WBC), platelets, hemoglobin levels, and total leukocyte counts were among the parameters measured. Leukocyte subtypes, including neutrophils, lymphocytes, monocytes, eosinophils, and basophils, were also examined. Compared with the Sham Control, Vehicle Control, and PNN Perse groups, rats exposed to EBRO exhibited a marked increase in basophil and eosinophil counts. On the other hand, following the 7th day of EBRO exposure, there was a significant decrease in the levels of neutrophils, lymphocytes, monocytes, RBCs, hemoglobin, WBCs, and platelets. However, these hematological parameters gradually recovered after receiving PNN for an extended period of time. PNN dose-dependent efficacy was demonstrated by the fact that rats given the higher PNN100 dose showed significantly greater improvements in their overall blood profile than those given the lower PNN50 dose. Furthermore, when VB12 (30) was administered in combination with the standard PNN50 dose, there were notable decreases in basophils and eosinophils and increases in neutrophils, lymphocytes, monocytes, red blood cells, hemoglobin, white blood cells, and platelet counts. These results demonstrate the possible hematoprotective benefits of PNN against EBRO-induced hematological disorders, especially when combined with VB12 (30). The analysis of the CBC involved a one-way ANOVA, which produced an F-statistic of basophils F (7,49) = 1,488, (p < 0.01), Figure 14A. Eosinophils: F (7,49) = 1,460, (p < 0.01), Figure 14B. Neutrophils: F (7,49) = 8,605, (p < 0.01), Figure 14C. Lymphocytes: F (7,49) = 1,295, (p < 0.01), Figure 14D. Monocytes: F (7,49) = 401.9, (p < 0.01), Figure 14E. RBC: F (7,49) = 3,166, (p < 0.01), Figure 14F. Haemoglobin F (7,49) = 31,505, (p < 0.01), Figure 14G. WBC: F (7,49) = 32,997, (p < 0.01), Figure 14H. Platelets: F (7,49) = 175,209, (p < 0.01), Figure 14I.

### Neuroprotection by PNN in the reduction of gross morphological changes in EBRO-induced rat model of MS

#### Whole brain (WB)

- (WB-Image-A-B-C)

**FIGURE 15:**
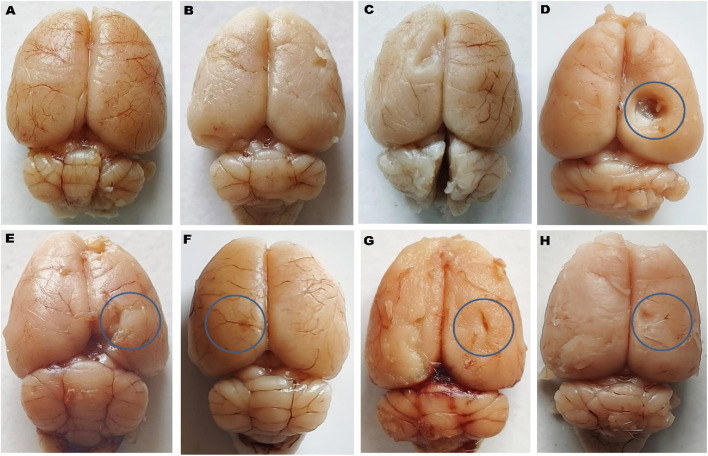
**(A–H)** Neuroprotection by PNN reduces gross morphological changes in the whole brain in the rat model of MS. In WB-image **(A)** Sham Control, **(B)** Vehicle Control, **(C)**PNN Perse, **(D)** EBRO, **(E)** EBRO + PNN50, **(F)** EBRO + PNN100, **(G)** EBRO + VB12 (30), **(H)** EBRO + VB12 (30) + PNN100 groups. (WB-Image-A-B-C) In sham control, vehicle control, and PNN Perse groups, the whole brain appeared normal and well-organised. There was no indication of swelling or abnormal coloration. (WB-Image-D) ICP administration EBRO group exhibited severe morphological degeneration. The brain was smaller overall, with evident cortical thinning and moderate ventricular enlargement. The cerebellum was diminished, and the brain surface exhibited abnormalities and tissue damage. (WB-Image-E) In the PNN50 group, the size of the brain showed some improvement, and the enlargement of the ventricles was less. (WB-Image-F) The PNN100 group displayed a whole brain that was normal in size and shape, with well-developed cortical and cerebellar structures. (WB-Image-G) The EBRO + VB12 (30) group showed enhanced brain morphology and less structural damage. (WB-Image-H) The EBRO + VB12 (30) + PNN100 group showed normal brain sizes and cerebellar structures with no observable surface abnormalities. The combined therapy PNN100 and VB12 (30) produces a synergistic effect in the whole brain.

**FIGURE 16:**
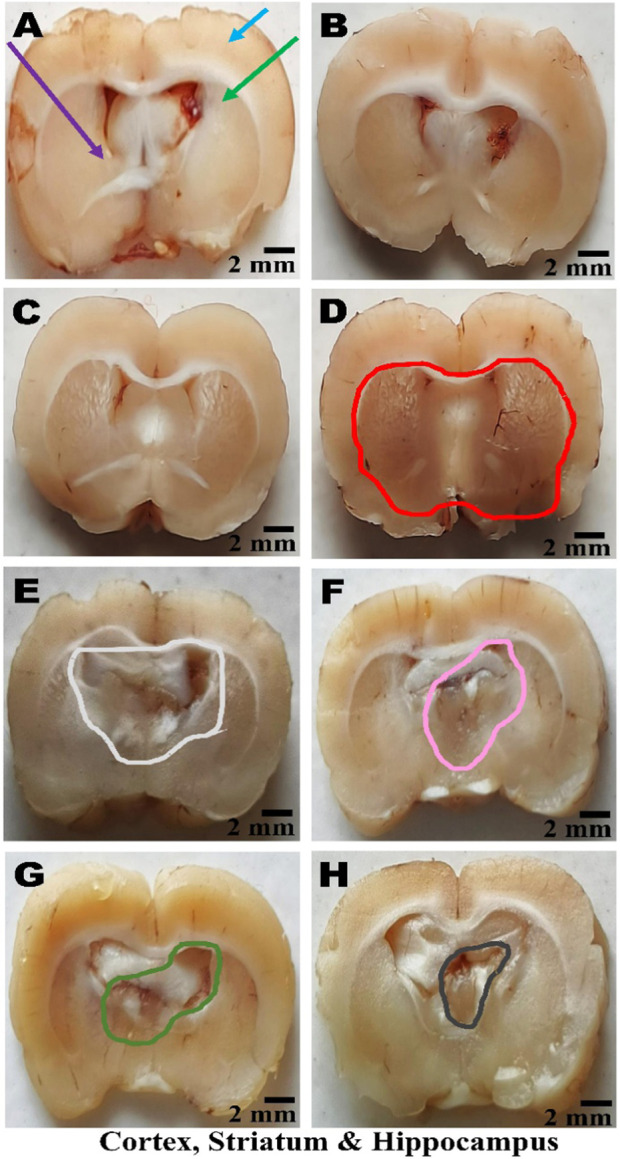
**(A–H)** Neuroprotection by PNN reduces gross morphological changes in cerebral cortex, hippocampus, and striatum in rat models of MS. (Coronal-Section-Image) **(A)** Sham Control, **(B)** Vehicle Control, **(C)**PNN Perse, **(D)** EBRO, **(E)** EBRO + PNN50, **(F)** EBRO + PNN100, **(G)** EBRO + VB12 (30), and **(H)** EBRO + VB12 (30) + PNN100 groups. A gross morphological study of the cerebral cortex, hippocampus, and striatum indicated that PNN had certain effects in an EBRO-induced animal model of multiple sclerosis. (Coronal-Section-Image-A-B-C) In the sham control, vehicle control, PNN Perse groups displayed healthy brain regions and sufficient white matter as well as normal size, shape, and structure. (Coronal-Section-Image-D) The EBRO group exhibited wide-ranging structural damage. The cerebral cortex had thin, irregular surface outlines that mediated cortical atrophy. The hippocampus was smaller and flattened, reflecting demyelination. The striatum appeared to be smaller and less distinct. (Coronal-Section-Image-E) The EBRO + PNN50 group resulted in moderate structure preservation at those sites. Cerebral cortex thickness was restored, the hippocampus was enhanced, and the striatal borders were recovered. (Coronal-Section-Image-F) The EBRO + PNN100 group showed improved gross morphology as well as some recovery in size, shape, and structure. The cerebral cortex showed improved structural integrity with distinct outlines; the hippocampus regained almost the same volume and shape as initially; and the striatum was well-defined, indicating a greater effect. (Coronal-Section-Image-G) EBRO + VB12 (30) improved size, shape, and structure in the cerebral cortex, hippocampus, and striatum. (Coronal-Section-Image-H) In the EBRO + VB12 (30) + PNN100 group, the retained size, shape, and structure in the cerebral cortex, hippocampus, and striatum were shown. Cortical areas were free from atrophy, and the hippocampus remained normally curved and maintained its normal volume. Collectively, the findings suggest a potential synergistic effect in attenuating EBRO-induced morphological alterations, which may offer therapeutic promise in mitigating multiple sclerosis-associated gross structural changes in the cerebral cortex, hippocampus, and striatum of the rat brain. [Note: Cerebral Cortex denoted by light blue colour, hippocampus denoted by green colour, Striatum denoted by purple colour, EBRO group denoted by red colour, EBRO + PNN50 group denoted by olive green colour, EBRO + PNN100 group denoted by pink colour, EBRO + VB12 (30) group denoted by orange colour, EBRO + VB12 (30) + PNN100 group denoted by black colour].

**FIGURE 17:**
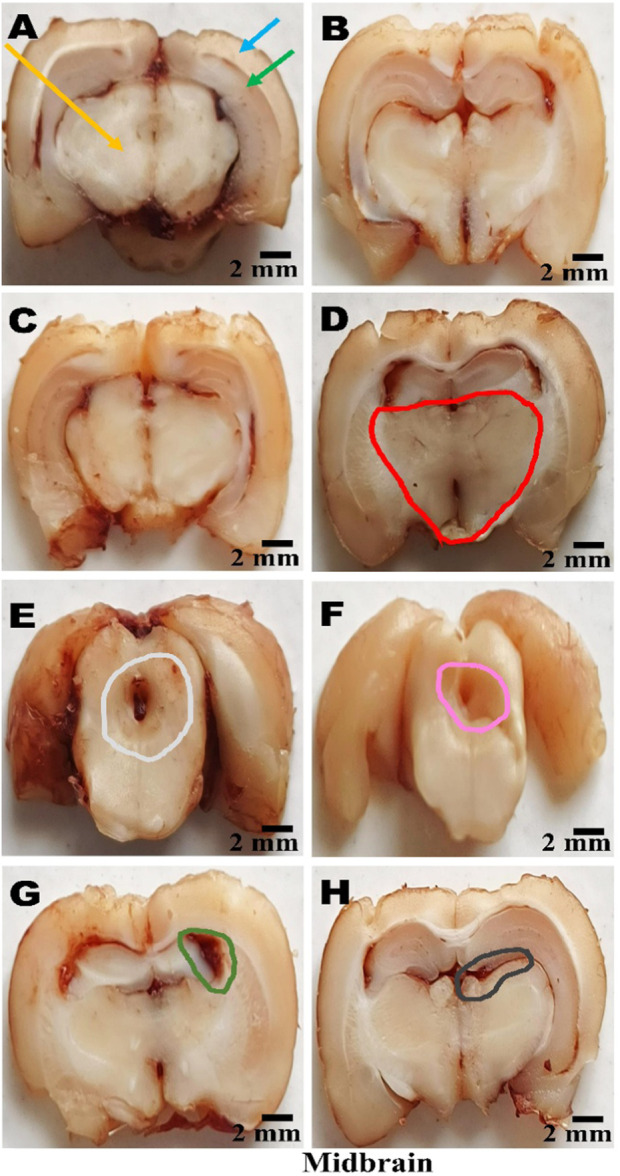
**(A–H)** Neuroprotection by paeoniflorin reduces gross morphological changes in the midbrain in rat models of MS. (Coronal-Section-Image) **(A)** Sham Control, **(B)** Vehicle Control, **(C)**PNN Perse, **(D)** EBRO, **(E)** EBRO + PNN50, **(F)** EBRO + PNN100, **(G)** EBRO + VB12 (30), and **(H)** EBRO + VB12 (30) + PNN100 groups. (Coronal-Section-A-B-C) In the sham control, vehicle control, and PNN Perse groups, the midbrain was structurally normal with maintained volume, smooth surface morphology, and distinct borders, indicating normal structure and lack of pathological changes. (Coronal-Section-Image-D) The neurotoxin EBRO group exhibited abnormalities of the midbrain. Demyelination was evidenced by regional atrophy, surface irregularities, and reduced volume. (Coronal-Section-Image-E) In the PNN50, there is a partial restoration of midbrain structure. Although some variability remained, the overall structure was recovered. (Coronal-Section-image-F) The EBRO + PNN100 group displayed normal midbrain volume and surface appearance, demonstrating the PNN100 dose-dependent effect. (Coronal-Section-Image-G) The EBRO + VB12 (30) group showed retained morphological preservation and minimal surface abnormalities. (Coronal-Section-Image-H) The EBRO + VB12 (30) + PNN100 group exhibited restored midbrain structure with no observable signs of damage. Collectively, the findings suggest a potential synergistic effect in attenuating EBRO-induced morphological alterations, which may offer therapeutic promise for mitigating multiple sclerosis-associated gross structural changes in the midbrain of the rat. [Note: Cerebral Cortex denoted by light blue colour, hippocampus denoted by green colour, midbrain denoted by yellow colour, EBRO group denoted by red colour, EBRO + PNN50 group denoted by green colour, EBRO + PNN100 group denoted by pink colour, EBRO + VB12 (30) group denoted by orange colour, EBRO + VB12 (30) + PNN100 group denoted by black colour].

**FIGURE 18:**
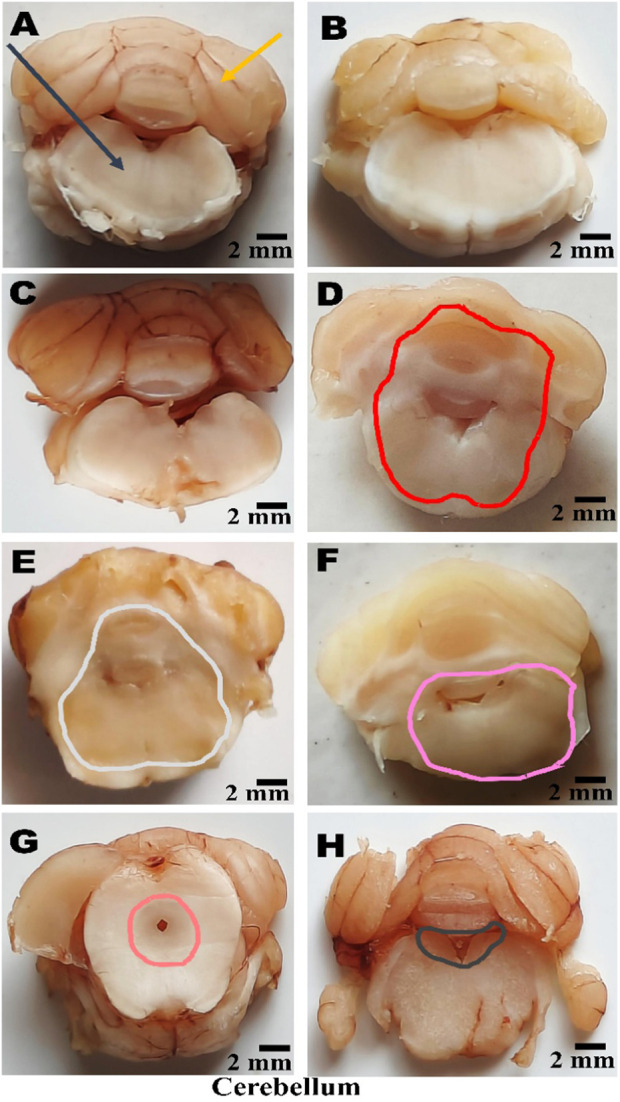
**(A–H)** Neuroprotection by PNN reduces gross morphological changes in cerebellum in rat models of MS. (Coronal-Section-Image) **(A)** Sham Control, **(B)** Vehicle Control, **(C)**PNN Perse, **(D)** EBRO, **(E)** EBRO + PNN50, **(F)** EBRO + PNN100, **(G)** EBRO + VB12 (30), and **(H)** EBRO + VB12 (30) + PNN100 groups. (Coronal-Section-A-B-C) In the sham control, vehicle control, and PNN Perse groups, the cerebellum showed normal morphology, with well-defined folia, smooth surface, and retained volume, indicating healthy tissue with no pathological changes. (Coronal-Section-D) In the EBRO group, the cerebellum exhibited atrophy characterized by folial flattening, irregular borders, and a reduction in overall tissue volume. These morphological alterations are consistent with demyelination and are observed in multiple sclerosis. (Coronal-Section-E) The EBRO + PNN50 groups showed a partially maintained cerebellar structure. While some surface abnormalities, tissue volume, and folia clarity recover. (Coronal-Section-F) The EBRO + PNN100 group exhibited restored cerebellar morphology. (Coronal-Section-image-G) In the EBRO + VB12 (30) group, restored cerebellar size, shape, and structure were displayed. (Coronal-Section-Image-H) The EBRO + VB12 (30) + PNN100 group showed retained size, shape, and structure in the cerebellum. [Note: Note: Cerebellum denoted by blue colour, midbrain yellow denoted by green colour, EBRO + PNN50 group denoted by green colour, EBRO + PNN100 group denoted by pink colour, EBRO + VB12 (30) group denoted by orange colour, EBRO + VB12 (30) + PNN100 group denoted by black colour].

In the sham control, vehicle control, and PNN Perse groups, normal brain structures were displayed, with a full-size shape and structure. (WB-Image-D) The neurotoxin EBRO group showed profound structural loss, including reduced brain weight, surface abnormalities, and tissue volume loss. (WB-image-E) The EBRO + PNN50 group showed restoration of brain morphology and small structural improvement, as well as a relative brain body weight ratio. (WB-Image-F) In the EBRO + PNN100 group, a recovery of brain structure and normal gross morphology was displayed, indicating a dose-dependent effect. (WB-Image-G) The EBRO + VB12 group exhibited a moderate effect with improved brain volume and size, shape, and structure. (WB-Image-H) In the EBRO + VB12 (30) + PNN100 group, displayed recovery of brain morphology, Figures 15A–H.

ii. Cerebral Cortex, Hippocampus, and Striatum

(Coronal-Section-A–B-C) In the (A) Sham Control, (B) Vehicle Control, and (C) PNN Perse group conditions, the cerebral cortex, hippocampus, and striatum exhibited preserved structure and no demyelination. Brain regions maintained normal volume, cellular structure, and surface morphology, and these findings confirm that myelin. (Coronal-Section-D) The EBRO neurotoxin group showed demyelination, cortical thinning, hippocampal structural disruption, and striatal atrophy. (Coronal-Section-E) In the EBRO + PNN50 group, definitive structural regeneration was observed, as evidenced by moderate tissue structural repair and attenuation of structural defects. (Coronal-Section-F) The EBRO + PNN100 group exhibited a dose-dependent effect, evidenced by extensive improvement, preserved cortical structure, and restoration of hippocampal and striatal structure. (Coronal-Section-G) The EBRO + VB12 (30) group showed substantial improvement, including enhanced cortical surface morphology and partial restoration of hippocampal and striatal tissue. (Coronal-Section-H) The EBRO + VB12 (30) + PNN100 group demonstrated improvement, with cerebral cortex, hippocampus, and striatum brain morphology in normal size, shape, and structure, Figures 16A–H.

iii. Cerebral Cortex, Hippocampus, and Midbrain

(Coronal Sections-Image-A-B-C) In the (A) Sham Control, (B) Vehicle Control, and (C) PNN Perse groups, the midbrain exhibited a well-preserved structure with no demyelination. (Coronal-Section-Image-D) The EBRO group demonstrated midbrain demyelination. (Coronal-Section-Image-E) The EBRO + PNN50 group demonstrated partial recovery, evidenced by moderate structural improvement and decreased midbrain degeneration. (Coronal-Section-Image-F) The EBRO + PNN100 group restored tissue structure and midbrain morphology. (Coronal-Section-Image-G) The EBRO + VB12 (30) group showed leading to increased midbrain structural features and tissue repair. (Coronal-Section-image-H) The EBRO + VB12 (30) + PNN100 group exhibited restoration of midbrain size, shape, and structure, Figures 17A–H.

iv. Cerebellum

(Coronal Sections-A-B-C) The cerebellum of the (A) sham control, (B) vehicle control, and (C) PNN Perse groups exhibited preserved structure without demyelination. The cerebellar layers remained intact, with normal volume, cellular organisation, and foliation, indicating preserved structural stability and myelin sheath integrity. (Coronal-Section-D) The EBRO group exhibited pronounced cerebellar demyelination, evidenced by folial loss, reduced tissue mass, and widespread cellular disorganisation. (Coronal-Section-E) The EBRO + PNN50 group exhibited partial recovery, with subtle improvements in cerebellar structure and a reduction in cortical layer damage. (Coronary-Section-F) The EBRO + PNN100 group demonstrated marked improvement and dose-dependent effect, as evidenced by the reorganisation of cerebellar size and shape, and restoration of normal structural morphology. (Coronal Section-Image-G) The EBRO + VB12 (30) group showed restoration of cerebellar size, shape, and structure. (Coronal-Section-Image-H) The EBRO + VB12 (30) + PNN100 group displayed more improvement, cerebellar morphology, Figures 18A–H.

### PNN neuroprotective role in mitigating EBRO-induced demyelination volume in MS rat model

#### In the cortex, hippocampus, and striatum regions

**FIGURE 19:**
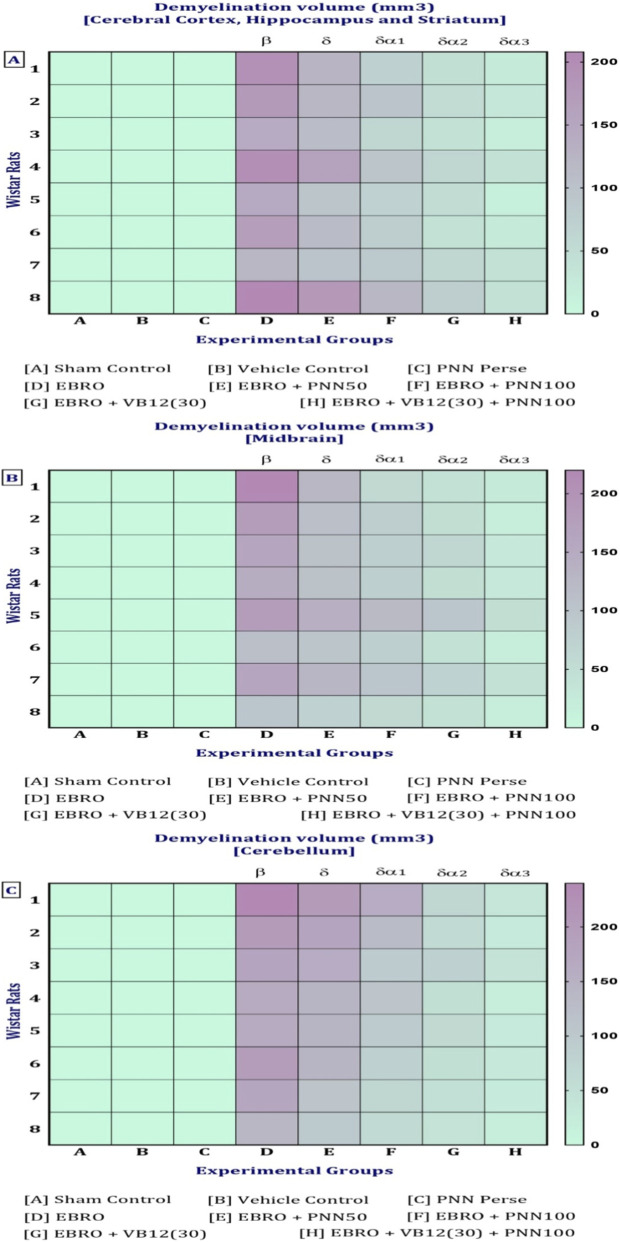
**(A–C)** PNN neuroprotective role in mitigating EBRO-induced demyelination volume alterations in certain rat brain regions, including the cerebral cortex, hippocampus, striatum **(A)**, mid-brain, **(B)** cerebellum **(C)**. Statistical analysis was performed using a one-way ANOVA followed by Tukey’s *post hoc* test to determine significant differences among groups **(A–C)**. Data were presented as mean ± standard deviation (SD), with statistical significance set at *p* < 0.01. Each experimental group consisted of eight wistar rats (n = 8). β v/s Sham Control, Vehicle Control, and PNN Perse; δ v/s EBRO; δα1 v/s EBRO + PNN50; δα2 v/s EBRO + PNN100, EBRO + PNN50; and δα3 v/s EBRO + VB12 (30), EBRO + PNN100, EBRO + PNN50.

No apparent demyelination was observed in the cerebral cortex, hippocampus, and striatum of rats in the sham control, vehicle control, and PNN Perse groups. However, a marked increase in demyelination was observed on the 7th day of EBRO induction relative to the control groups. Treatment with PNN50 and PNN100 significantly reduced the extent of demyelination relative to the EBRO group. Additionally, there was a significant decrease in demyelination in the VB12 (30). Interestingly, in EBRO-induced rats, the group that received VB12 (30) and high-dose PNN showed the greatest recovery in demyelination volume. The analysis of demyelination volume used a one-way ANOVA, which produced an F-statistic for the cerebral cortex, hippocampus, and striatum (F (7,49) = 144.9, p < 0.01; Figure 19A).

#### In the midbrain and cerebellum region

The midbrain and cerebellum regions of rats in the Sham Control, Vehicle Control, and PNN Perse groups showed no signs of demyelination. But after being exposed to EBRO for 7th days, there was a noticeable rise in demyelination when compared to the sham control, vehicle control, and PNN Perse groups. When PNN50 and PNN100 were administered, demyelination was significantly reduced compared with the EBRO group; the effect was more pronounced in the PNN100 group than in the PNN50 group. Compared with the EBRO group, the VB12 (30) group also showed significantly less demyelination. Significantly, in EBRO-induced rats, the combination treatment of VB12 (30) and PNN100 produced the greatest recovery of demyelination volume. The analysis of the demyelination volume involved a one-way ANOVA, which produced an F-statistic of midbrain F (7,49) = 103.5, (p < 0.01), Figure 19B. Cerebellum: F (7,49) = 148.7, (p < 0.01), Figure 19C.

### Neuroprotection by paeoniflorin in the reduction of H&E-stained histopathological changes in EBRO-induced rat model of multiple sclerosis

#### Cerebral cortex

**FIGURE 20:**
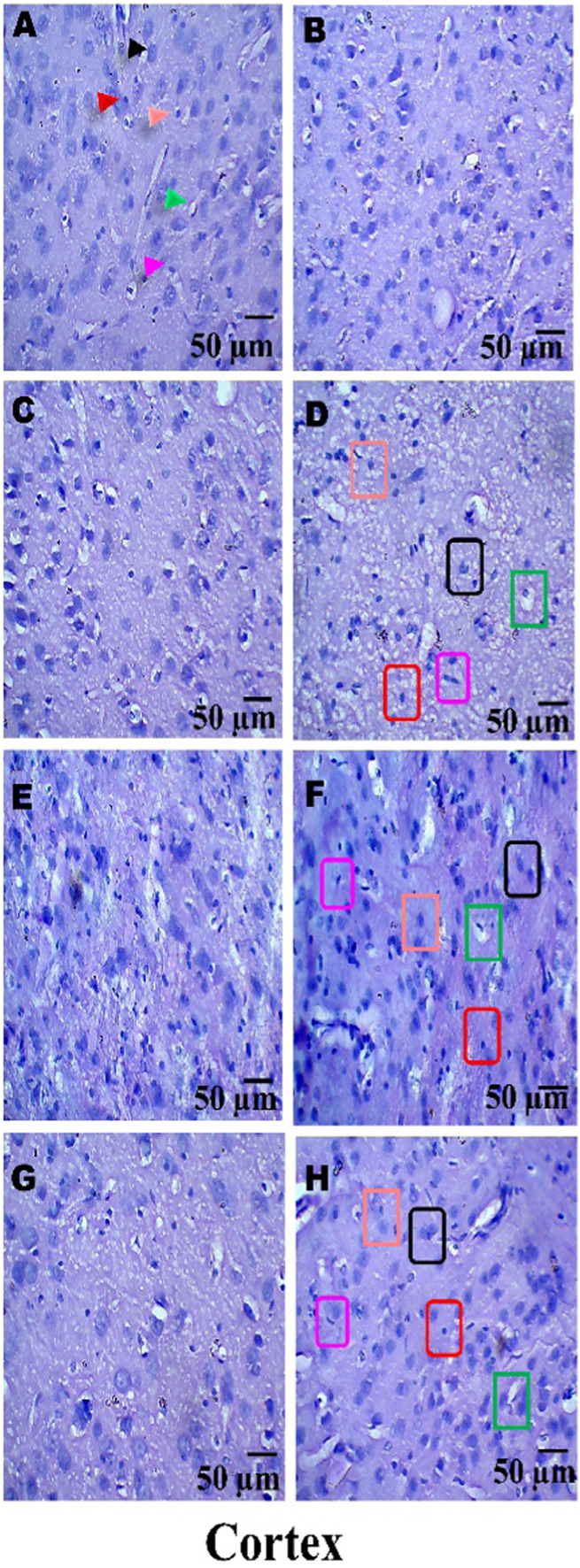
**(A–H)** Neuroprotection by paeoniflorin reduces H&E-stained histopathological changes in the coronal section of the cerebral cortex in the rat model of multiple sclerosis. Haematoxylin and Eosin (H&E) staining is a crucial technique in neurohistology, particularly for studying brain tissues. It enables us to distinguish among various cell types, including oligodendrocytes, astrocytes, and microglia, which comprise the central nervous system’s glial population. Each cell type has distinct histological features that can be visualized using the H&E staining method viewed under a fluorescence microscope at ×40 magnification. The images include **(A)** Sham control, **(B)** Vehicle control, **(C)** PNN Perse, **(D)** EBRO, **(E)** EBRO + PNN50, **(F)** EBRO + PNN100, **(G)** EBRO + VB12 (30), and **(H)** EBRO + VB12 (30) + PNN100. There are branching neurons in the cortex. These are either triangular pyramidal cells or cube-shaped stellate cells, both with vesicular shapes. Astrocytes are star-shaped glial cells that play a crucial role in structurally and metabolically supporting neurons. The nuclei of astrocytes are larger and have lighter staining than those of oligodendrocytes. They are oval and appear in a lighter shade of purple to blue; the orange trigone in these initial micro-H&E panels indicates astrocytes. As you can see in the first three (Micro-H&E Panels-A-B-C) group, the pyramidal cells in the brain’s external pyramidal (EP) layer are arranged in a typical way and shown by the black trigon. These first micro-H&E panels show oligodendrocytes marked by a red trigon. The cytoplasm is well preserved and maintains good structural integrity. The green cube highlights regular blood capillaries with a uniform distribution. The pink trigon in these initial Micro-H&E Panels was identified as microglia. Microglia possess small, elongated nuclei that stain darkly with hematoxylin, appearing deep blue to purple. These small, rod-shaped nuclei, such as those in an inflammatory response, can enlarge and become rounded during activation. The cytoplasm appears reduced, while astrocytes are hypertrophied, as shown by the orange cube. (Micro-H&E-Panel-D) In the neurotoxin EBRO group, altered and aberrant shapes of pyramidal and glial cells are seen. (Micro-H&E-Panel-E) The EBRO + PNN50 group showed some morphological restoration compared with the neurotoxin treatment. (Micro-H&E-Panel-F) The EBRO + PNN100 group displays more regenerated pyramidal and glial cells. All cells exhibit improved structural and morphological integrity, increased nuclear density, and reduced areas of vacuity. High-dose PNN100 had a greater impact on cortical regions affected by EBRO. (Micro-H&E-Panel-G) The cortical tissues in the EBRO + VB12 (30) group show fewer inflammatory cell clusters, less damage to oligodendrocytes and astrocytes, and reduced capillary congestion. Additionally, there is a notable improvement in the structural integrity of pyramidal cells, accompanied by morphological changes. (Micro-H&E-Panel-H). EBRO + VB12 (30) + PNN100 restored pyramidal cell morphology and numbers, as well as the numbers of oligodendrocytes, astrocytes, and microglia, indicating a dose-dependent effect of PNN. It also demonstrates increased neuroprotection, indicating the combined treatment’s synergistic effect. Blood vessels showed dilation and better distribution, as indicated by the green cube. The findings suggest that administering PNN100 + VB12 (30) significantly ameliorates EBRO-induced cerebral tissue changes in adult Wistar rats. (Note: The black cube indicates pyramidal cells, the pink cube represents microglia, the red cube denotes oligodendrocytes, the orange cube denotes astrocytes, and the green cube marks blood vessels) (Magnification: ×40; Scale bar: 50 μm).

We observed cortical neurons stained with hematoxylin and eosin in a fluorescent microscope under ×40 magnification. (Micro-H&E-Panel-A-B-C) The study demonstrated normal morphology and patterns of pyramidal and glial neurons. (Micro-H&E-Panel-D) The EBRO neurotoxin group had larger capillaries with glial cells and irregular pyramidal cells within them. (Micro-H&E-Panel-E) In the EBRO + PNN50 group, some morphological restoration was observed after neurotoxin treatment. (Micro-H&E-Panel-F) The EBRO + PNN100 group restored pyramidal and glial cells. This restoration was achieved by maintaining structural and morphological integrity, including nuclear density restoration. (Micro-H&E-Panel-G) In the EBRO + VB12 (30) group, pyramidal cells displayed some improvement and were dispersed in the cortical tissues. (Micro-H&E-Panel-H) The EBRO + VB12 (30) + PNN100 group produced a marked improvement in the reduction of inflammatory cell clusters, clumping of oligodendrocytes, astrocyte microglial lessening, capillary congestion relief, and improved structural morphology of pyramidal cells, Figures 20A–H.

#### Hippocampus

**FIGURE 21:**
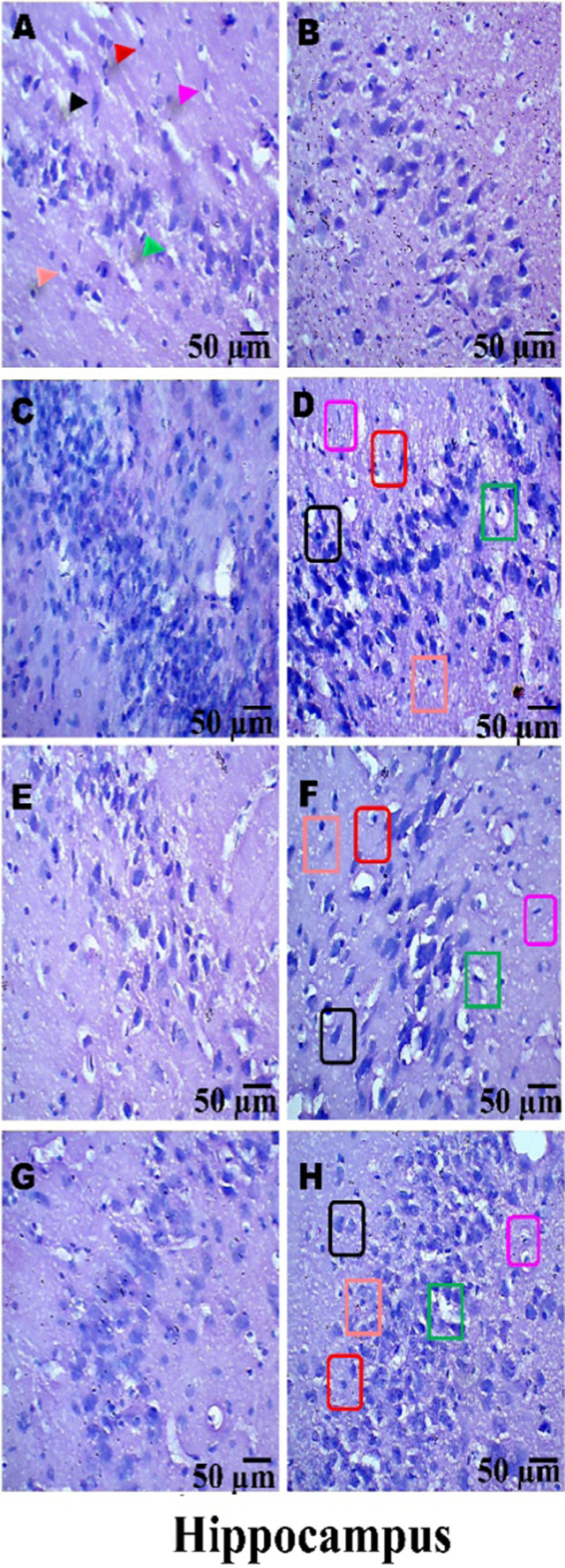
**(A–H)** Neuroprotection by paeoniflorin reduces H&E-stained histopathological changes in coronal section-hippocampus in rat model of multiple sclerosis. Hematoxylin and Eosin (H&E) staining is essential in neurohistology, especially for examining brain tissues such as the hippocampus. Each cell type exhibits unique histological characteristics that can be observed under a fluorescent microscope with H&E staining at ×40 magnification. The picture presented includes **(A)** Sham Control, **(B)** Vehicle Control, **(C)** PNN Perse, **(D)** EBRO, **(E)** EBRO + PNN50, **(F)** EBRO + PNN100, **(G)** EBRO + VB12 (30), and **(H)** EBRO + VB12 (30) + PNN100. The hippocampal tissue displays densely packed pyramidal neurons in the CA1 and CA3 regions and granular cells in the dentate gyrus, each exhibiting distinct vesicular morphology. Astrocytes, which are star-shaped glial cells, are essential for providing structural and metabolic support to neurons in the hippocampus. They are oval and appear in a lighter shade of purple to blue, shown by the orange trigon. The (Micro-H&E-Panel-A-B-C) group displayed the typical arrangement of pyramidal cells in the CA1 and CA3 regions and normal glial cells. Oligodendrocytes feature small, round nuclei that are darkly stained with hematoxylin, and their cytoplasm exhibits strong preservation and structural integrity, as shown by the red trigon. The green trigon indicates the regular distribution of blood capillaries throughout the hippocampus. (Micro-H&E-Panel-D) In the neurotoxin EBRO group, the pyramidal cells appear altered, have an unusual shape, and damage glial cells. (Micro-H&E-Panel-E) The EBRO + PNN50 group showed partial restoration of morphology compared with the neurotoxin-treated group. (Micro-H&E-Panel-F) In the EBRO + PNN100 group, the structural and morphological integrity of the cells has improved, with a higher nuclear density and fewer empty spaces, indicating reduced inflammation and repaired capillaries, as highlighted by the green cube. The high-dose PNN100 treatment demonstrated a more pronounced effect on repairing EBRO-damaged hippocampal tissue. (Micro-H&E-Panel-G) The EBRO + VB12 (30) group shows a noticeable decrease in inflammatory cell clusters, reduced aggregation of oligodendrocytes with astrocytes, and improved capillary structure. The integrity of pyramidal cells is significantly enhanced, as shown by the black cube. (Micro-H&E-Panel-H) In the EBRO + VB12 (30) + PNN100 group, there is a partially restored along with an increase in the number of pyramidal cells, oligodendrocytes, astrocytes, and microglia, suggesting a dose-dependent neuroprotective effect of PNN. The Micro-H&E-Panel hippocampal picture from this group shows reduced inflammation, improved nuclear morphology, and better-preserved blood vessels, as highlighted by the green cube. The combination treatment of the PNN100 + VB12 (30) group improves the hippocampal damage induced by EBRO in adult Wistar rats. (Note: The black cube indicates pyramidal cells, the pink cube represents microglia, the red cube arrow denotes oligodendrocyte cells, the orange cube denotes astrocytes, and the green cube marks blood vessels.) (Magnification: ×40; Scale bar: 50 μm).

We performed histological analysis of the hippocampal slide from adult Wistar rats using a fluorescence microscope at 40× magnification. (Micro-H&E-Panel-A-B-C) groups, the pyramidal neurons in the pyramidal layer had euchromatic nuclei, large cytoplasm, and well-organized capillary structures without damage to glial cells. (Micro-H&E-Panel-D) The EBRO neurotoxin group shows some decreased nuclei that appear condensed. The degenerated regions are not clearly defined with glial cells, congested capillaries, empty spaces, and lines of inflammatory aggregation. (Micro-H&E-Panel-E) The EBRO + PNN50 group showed slightly improved demyelination and other pathological changes in the hippocampal slide. (Micro-H&E-Panel-F) The EBRO + PNN100 group exhibited better quality and organisation of the pyramidal cell layer, with some restoration of blood capillaries. Additionally, there was greater recovery for oligodendrocytes, astrocytes, and microglia. (Micro-H&E-Panel-G) The EBRO + VB12 (30) group significantly improved the structural integrity of pyramidal cells in the hippocampus slide. (Micro-H&E-Panel-H) The EBRO + VB12 (30) + PNN100 group showed decreased clusters of inflammation, regrowth of glial cells, less capillary congestion, and enhanced recovery of pyramidal cells, as evidenced by the presence of cells with black pyknotic nuclei, Figures 21A–H.

#### Striatum

**FIGURE 22:**
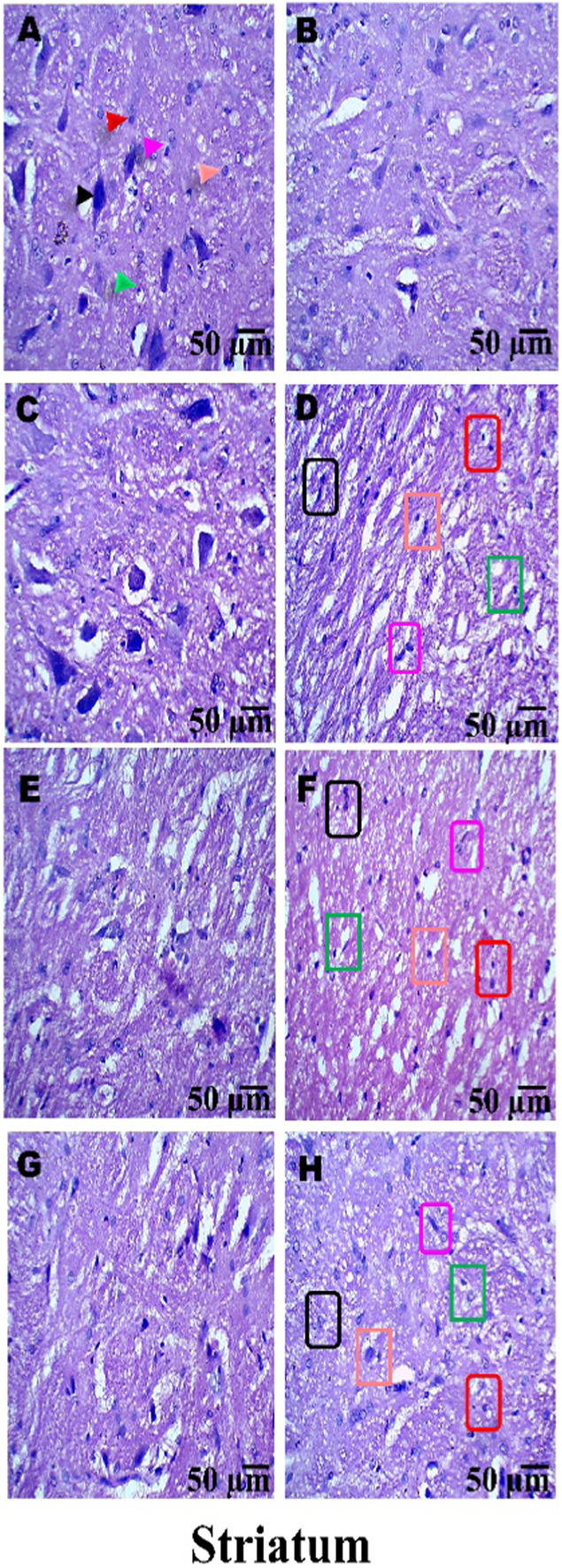
**(A–H)** Neuroprotection by PNN reduces H&E-stained histopathological changes in the coronal section of the striatum in the rat model of MS. Hematoxylin and Eosin (H&E) staining is a crucial method in neurohistology, particularly for examining brain tissues such as the striatum in experimental rat models of MS. Each of these cell types exhibits unique histological characteristics that can be observed under a fluorescent microscope at ×40 magnification. The study presents images from the following treatment groups: **(A)** Sham Control, **(B)** Vehicle Control, **(C)** PNN Perse, **(D)** EBRO, **(E)** EBRO + PNN50, **(F)** EBRO + PNN100, **(G)** EBRO + VB12 (30), **(H)** EBRO + VB12 (30) + PNN100. In the striatum, the tissue exhibits a distinct arrangement of large, multipolar neurons, including medium spiny neurons, vital for motor control. Astrocytes, the star-shaped glial cells, are crucial in providing structural and metabolic support to neurons. The nuclei of astrocytes are larger and more lightly stained than those of oligodendrocytes, appearing in a lighter purple or blue color. In the coronal sections of the Striatum, the orange trigon indicates astrocytes. (Micro-H&E-Panel-A-B-C) All groups show a normal condition. Neuron indicated a black trigon. Oligodendrocytes have small, round nuclei that stain darkly with hematoxylin, while their cytoplasm remains intact and well-preserved, as shown by the red trigon. The green cube illustrates the typical distribution of blood capillaries across the striatum. (Micro-H&E-Panel-D) In the Neurotoxin EBRO group, the pyramidal cells exhibit altered shapes, as indicated by the black trigon, while the pink trigon points to microglia. In their resting state, microglial nuclei are small and elongated, appearing dark blue to purple when stained. However, during activation, such as in response to inflammation or injury, their nuclei enlarge and become round. The cytoplasm of microglia is often faint, and their delicate branching processes are less visible with H&E staining. The green cube arrow in these sections shows distended blood capillaries within the striatum. The red arrow points to areas with scattered, ill-defined oligodendrocytes, whereas the orange circle indicates hypertrophied astrocytes. (Micro-H&E-Panel-E) The EBRO + PNN50 group showed signs of recovery, with moderate restoration of neuronal integrity in oligodendrocytes, astrocytes, and microglia. (Micro-H&E-Panel-F) In the EBRO + PNN100 group, there is clear regeneration of both neurons and glial cells, as indicated by the black, red, orange, pink, and green cubes, and by repaired capillaries highlighted by the green cube, demonstrating restored vascular integrity. High-dose PNN100 significantly affected the striatum, greatly improving the tissue impacted by EBRO. (Micro-H&E-Panel-G) In the EBRO + VB12 (30) group, there is an apparent reduction in inflammatory cell clusters, less aggregation of oligodendrocytes and astrocytes, and decreased capillary congestion. The structural integrity of medium spiny neurons has improved significantly. (Micro-H&E-Panel-H) In the EBRO + VB12 (30) + PNN100 group, neuronal and glial cell mitigated is apparent. The numbers and structure of pyramidal and glial cells were restored, indicating a dose-dependent neuroprotective effect of PNN100. The Micro-H&E-Panel coronal sections of the striatum reveal reduced inflammation, enhanced nuclear morphology, and improved blood vessels. These findings suggest that the combined PNN100 + VB12 (30) treatment significantly enhances striatum tissue changes induced by EBRO in adult Wistar rats. (Note: The black cube indicates pyramidal cells, the pink cube represents microglia, the red cube denotes oligodendrocyte cells, the orange cube denotes astrocytes, and the green cube marks blood vessels) (Magnification: ×40; Scale bar: 50 μm).

*Hematoxylin and Eosin (H&E) staining is essential in neurohistology, especially for examining brain tissues such as the hippocampus. Each cell type exhibits unique histological characteristics that can be observed using H&E staining under a fluorescent microscope at ×40 magnification*. (Micro-H&E-Panel-A-B-C) groups display oval morphology with distinct rounded nuclei and normal shape of glial cells. (Micro- H&E-Panel-D) Neurotoxin EBRO group is characterized by inflammation and demyelination, poor neuronal structure, capillary congestion, dispersed and wrapped oligodendrocytes, and damaged astrocytes and microglia. (Micro-H&E-Panel-E) The EBRO + PNN 50 group showed signs of recovery, with moderate restoration of neuronal integrity in oligodendrocytes, astrocytes, and microglia. (Micro-H&E-Panel-F) The EBRO + PNN 100 group showed lesion repair, as evidenced by improved neuronal morphology, decreased swelling of protoplasmic astrocytes, and a slight restoration of oligodendrocyte morphology. (Micro-H&E-Panel-G) In the EBRO + VB12 (30) group, low doses produce a slight increase in the composition of neurons, oligodendrocytes, and astrocytes, with lower levels of inflammatory agents and space. (Micro-H&E-Panel-H) In the EBRO + VB12 (30) + PNN100, shows a reduction in inflammatory cell aggregates and capillary congestion, the shape of oligodendrocytes, astrocytes, and microglia returns to normal, and there is regeneration of neuronal cells, Figures 22A–H.

#### Midbrain

**FIGURE 23:**
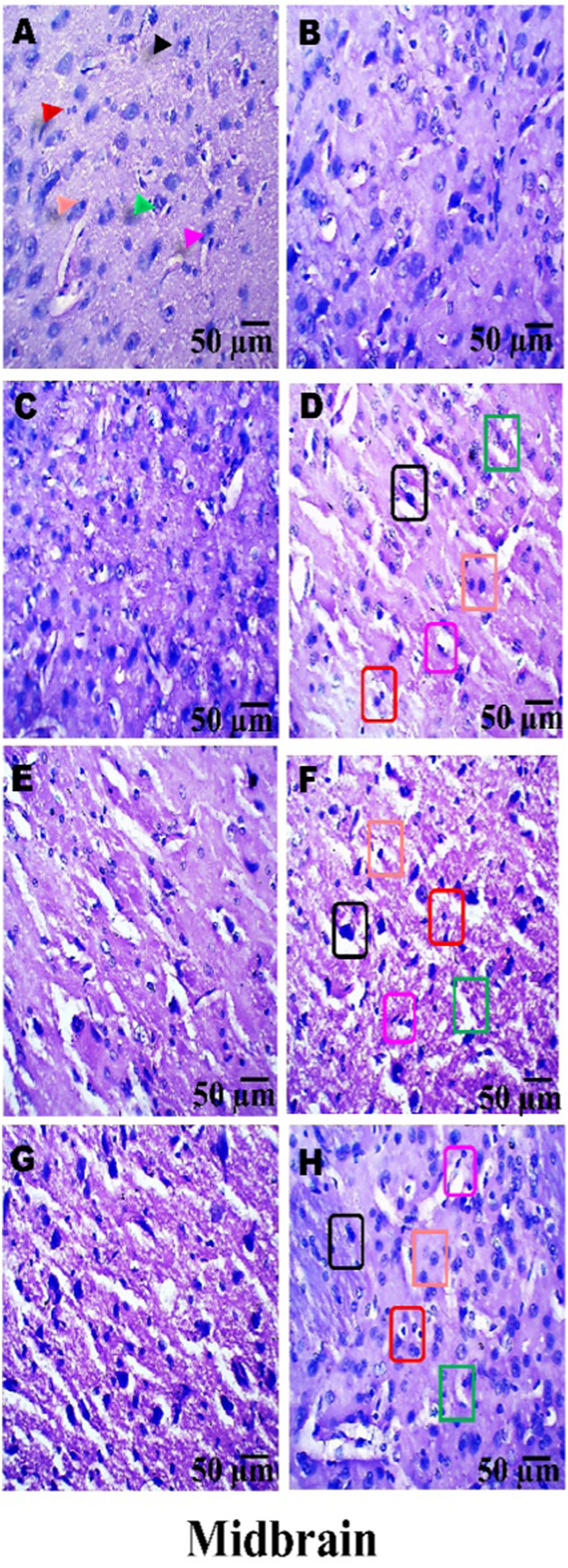
**(A–H)** Neuroprotection by PNN reduces H&E-stained histopathological changes in the coronal section of the midbrain in the rat model of MS. H&E staining is a crucial technique in neurohistology, particularly for examining brain tissues such as the midbrain in an experimental rat model of MS. Each cell type exhibits unique histological characteristics that can be observed under a fluorescent microscope at ×40 magnification. The H&E picture presented in this study shows the following treatment groups: **(A)** Sham Control, **(B)** Vehicle Control, **(C)** PNN Perse, **(D)** EBRO, **(E)** EBRO + PNN50, **(F)** EBRO + PNN100, **(G)** EBRO + VB12 (30), and **(H)** EBRO + VB12 (30) + PNN100. Astrocytes, star-shaped glial cells, play a crucial role in providing structural and metabolic support to neurons. The nuclei of astrocytes are more prominent and exhibit lighter staining than those of oligodendrocytes, often appearing in shades of light purple or blue. In the midbrain, the orange trigon indicates the location of the astrocytes. (Micro-H&E-Panel-A-B-C) groups show the size and shape of neurons and glial cells. The nuclei of oligodendrocytes are small and round, staining darkly with hematoxylin, while their cytoplasm maintains its structural integrity, as shown by the red trigon. The green cube shows regular blood capillaries that are evenly distributed throughout the midbrain. (Micro-H&E-Panel-D) The EBRO neurotoxin group shows neuronal and glial cell damage. The neuron’s shape changes, as indicated by the black triangle. The pink trigon points to microglia, which serve as the immune cells of the central nervous system (CNS), functioning as phagocytes to clear in debris and pathogens. The green cube highlights the presence of distended capillaries in the midbrain, while the red cube arrow points out the scattered and unclear oligodendrocytes. (Micro-H&E-Panel-E) The EBRO + PNN50 group showed signs of recovery in neurons, oligodendrocytes, astrocytes, microglia, and blood vessels, with moderate restoration of neuronal integrity. (Micro-H&E-Panel-F) In the EBRO + PNN100 group, regenerated pyramidal and glial cells are more prominent. Improvement in all cells’ structural and morphological integrity, with increased nuclear density and fewer empty spaces. The repaired capillaries are now more clearly visible, and the high-dose PNN100 treatment shows a more pronounced effect on the midbrain regions affected by EBRO. (Micro-H&E-Panel-G) In the EBRO + VB12 (30) group, fewer inflammatory cell clusters, reduced oligodendrocytes and astrocytes aggregation, and less capillary congestion. Moreover, the structural integrity of neurons has improved significantly. (Micro-H&E-Panel-H) In the EBRO + VB12 (30) + PNN100 group, there is a mitigated in the morphology and numbers of neurons, oligodendrocytes, astrocytes, and microglia, suggesting a dose-dependent neuroprotective effect of PNN. The Micro-H&E-Panel midbrain sections reveal decreased inflammation, better nuclear morphology, and enhanced integrity of blood vessels, as indicated by the green cube arrow. These results imply that the PNN100 + VB12 (30) combination greatly enhances midbrain tissue changes induced by EBRO in adult Wistar rats. (Note: The black cube indicates pyramidal cells, the pink cube represents microglia, the red cube denotes oligodendrocyte cells, the orange cube denotes astrocytes, and the green cube marks blood vessels). (Magnification: ×40; Scale bar: 50 μm).

The midbrain neuron population was studied by fluorescence microscopy at 40× magnification with hematoxylin and eosin staining. (Micro-H&E-Panel-A-B-C) groups exhibit typical neuronal and glial structures, including regular nuclei. Additionally, the oligodendrocytes, astrocytes, and microglia in these groups are undamaged and well-structured. (Micro-H&E-Panel-D) The EBRO group exhibited significant demyelination, resulting in loss of structural integrity in neurons and glia. (Micro-H&E-panel-E) The EBRO + PNN50 group moderately reduces demyelination and normalises glial cell numbers. (Micro-H&E-Panel-F) The EBRO + PNN100 group exhibited reduced degeneration and increased microglial regulation and integrity; neurons, oligodendrocytes, astrocytes, and microglia were distributed appropriately within the lesion. (Micro-H&E-Panel-G) The EBRO + VB12 (30) group showed some recovery in neurons, oligodendrocytes, astrocytes, and microglial cells. (Micro-H&E-Panel-H) The EBRO + VB12 + PNN100 showed marked recovery in the demyelinated area, with reduced gaps, microglial infiltration, and inflammatory elements. The astrocytes, oligodendrocytes, microglia, and neurons returned to near-normal morphological characteristics and sizes, with evidence of improved glial and neuronal distribution (Figures 23A–H).

#### Cerebellum

**FIGURE 24:**
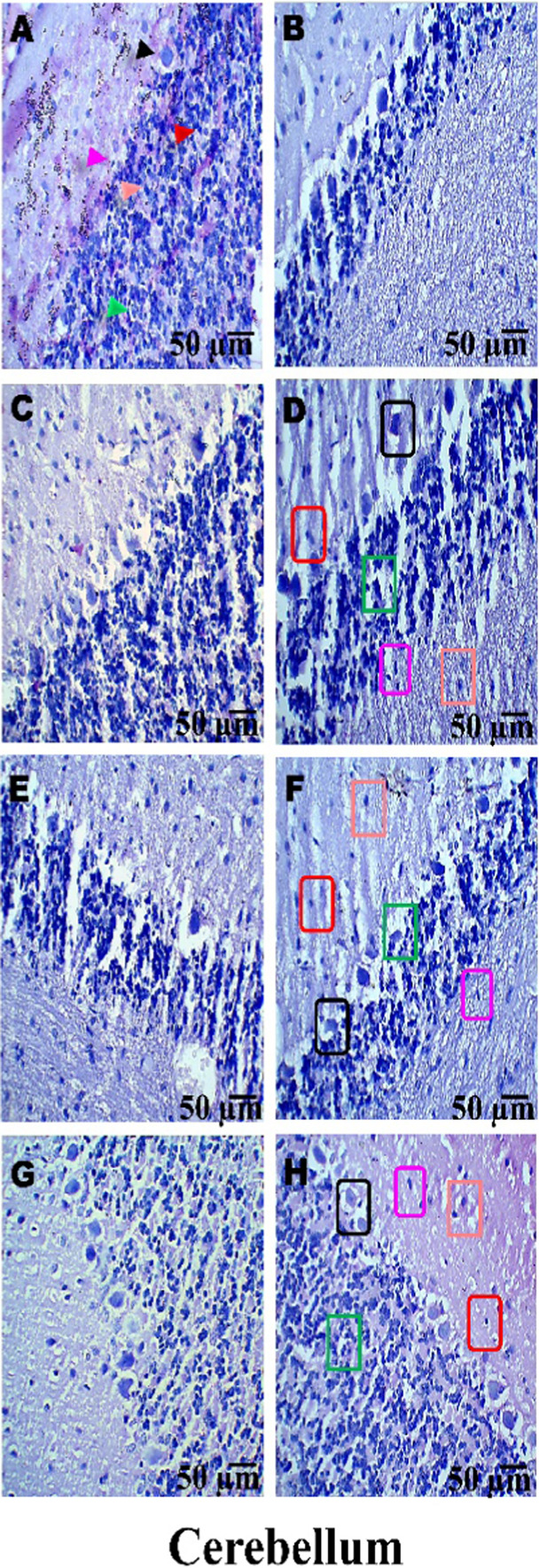
**(A–H)** Neuroprotection by PNN reduces H&E-stained histopathological changes in the coronal section of the cerebellum in the rat model of MS. Micro-H&E-Panel is a key technique in neurohistology, especially for analyzing brain tissues like the cerebellum in experimental rat models of MS. Each of these cells has unique histological features that can be observed using H&E-Panel under a fluorescence microscope at ×40 magnification. The picture presented in this study includes **(A)** Sham Control, **(B)** Vehicle Control, **(C)** PNN Perse, **(D)** EBRO, **(E)** EBRO + PNN 50, **(F)** EBRO + PNN 100, **(G)** EBRO + VB12 (30), and **(H)** EBRO + VB12 (30) + PNN 100. The cerebellum comprises well-organized layers, including the molecular layer, Purkinje cell layer, and granular layer, each containing specific neurons and glial cells. Astrocytes, star-shaped glial cells, provide structural and metabolic support to neurons. The nuclei of astrocytes are larger and lighter-staining than those of oligodendrocytes, typically appearing in shades of light purple or blue and orange trigon, indicating astrocytes in the cerebellar H&E-Panel. (Micro-H&E-Panel A, B, C) groups exhibit typical Purkinje cells and granule neuron configurations. The black trigon points to the Purkinje cells, situated in the cerebellum’s middle layer. The nuclei of oligodendrocytes are small, round, and dark-stained with hematoxylin, while their preserved cytoplasm indicates structural integrity, as shown by the red trigon. The green cube highlights regular blood capillaries evenly distributed throughout the cerebellum. (Micro-H&E-Panel-D) In the EBRO group, the arrangement of cerebellum neurons is altered, as shown by the black trigon. Microglia are dynamic and can change shape in response to injury, displaying small, elongated, dark-stained nuclei in a resting state and enlarged, rounded nuclei upon activation. The green marks distended blood vessels in the cerebellum, while the red cube denotes scattered, ill-defined oligodendrocytes. (Micro-H&E-Panel-E) The EBRO + PNN50 group showed a moderate reduction in demyelination and a normal oligodendrocyte population. (Micro-H&E-Panel-F) In the EBRO + PNN100 group, there was an increase in regenerated Purkinje and granule cells. This suggests that PNN has improved the structural and morphological integrity of all cell types, thereby reducing inflammation and promoting blood vessel repair in the cerebellum. (Micro-H&E-Panel-G) In the EBRO + VB12 (30) group, there are fewer clusters of inflammatory cells, along with less aggregation of oligodendrocytes, astrocytes, microglia, and reduced capillary congestion, as well as Purkinje cells. (Micro-H&E-Panel-H) in the EBRO + VB12 (30) + PNN100 group, there is a restoration of neuronal morphology and numbers, as well as of oligodendrocytes, astrocytes, microglia, and blood vessels, suggesting a dose-dependent neuroprotective effect of PNN. The findings suggest that the combination of PNN100 + VB12 (30) significantly enhances CBL tissue changes induced by EBRO in adult Wistar rats (Note: The black cube indicates pyramidal cells, the pink cube represents microglial cells, the red cube denotes oligodendrocyte cells, the orange cube denotes astrocytes, and the green cube marks blood vessels). (Magnification: ×40; Scale bar: 50 μm).

The micro-H&E sections of the cerebellum stained with hematoxylin and eosin show healthy, well-arranged Purkinje cells. (Micro-H&E-Panel-A-B-C) group, these have specific morphology and organization in neurons, oligodendrocytes, astrocytes, and microglia, forming tightly aggregated granular layer cells that exhibit specific shapes and sizes. (Micro-H&E-Panel D) The neurotoxin EBRO group was causing damage to the cerebellar layers. (Micro-H&E-Panel-E) The EBRO + PNN50 group showed a moderate reduction in demyelination and normal oligodendrocytes, astrocytes, and microglia. (Micro-H&E-Panel-F) The EBRO + PNN100 group exhibited diminished lesion area and a higher degree of microglial regulation and integrity; neurons, oligodendrocytes, astrocytes, and microglia were distributed properly within the lesion. (Micro-H&E-Panel-G) The EBRO + PNN100 indicated improved recovery, higher astrocyte, oligodendrocyte, microglial, and normal Purkinje cell morphology, and increased granular layer density. (Micro-H&E-Panel-H) The EBRO + VB12 (30) + PNN100 group decreased the clustering of neurons, glial cells, and blood vessels. The VB12 (30) + PNN 100 was fully restored after cerebellar deterioration (Figures 24A–H).

### Neuroprotection by PNN in the reduction of LFB-stained histopathological changes in EBRO-induced rat model of MS

#### Cerebral cortex

**FIGURE 25:**
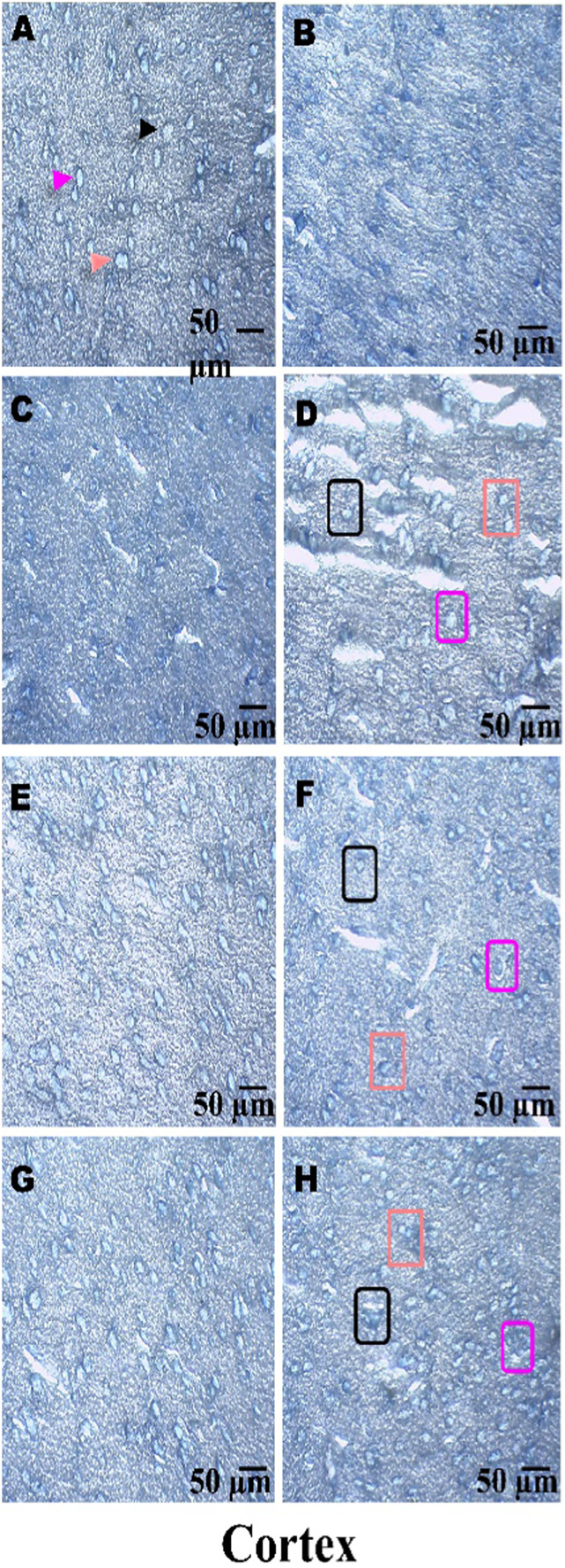
**(A–H)** Neuroprotection by PNN reduces LFB-stained histopathological changes in coronal section-cerebral cortex in rat model of MS. All images displayed on the Micro-LFB-Panel were taken at a ×40 magnification of cortical coronal sections of adult Wistar rats treated with PNN stained. LFB staining is specifically used to examine myelination; it stains the white matter blue and is employed to detect demyelination and remyelination. The experimental groups included **(A)** Sham Control, **(B)** Vehicle Control, **(C)** PNN Perse, **(D)** EBRO, **(E)** EBRO + PNN50, **(F)** EBRO + PNN100, **(G)** EBRO + VB12 (30), **(H)** EBRO + VB12 + PNN100. The (Micro-LFB-Panel-A-B-C) group showed normal myelination with elongation, as shown by a black trigon, and white matter tracts under the grey matter showed healthy myelin sheaths. Neurons were packed densely, and almost no vacuole formation was shown by the orange trigon, and no damage to the white matter. (Micro-LFB-Panel D) The EBRO group exhibited extensive demyelination; loss of blue staining in the LFB indicated severe damage to the myelin sheaths, with very prominent dead macrophages (pink trigon) and myelin breakdown products, vacuole formation indicative of tissue degeneration, and a spongy appearance in the affected areas. (Micro-LFB-Panel-E) The EBRO + PNN50 group shows moderate reductions in demyelination, with partial remyelination evident by restoration of LFB staining. (Micro-LFB-Panel-F) In the EBRO + PNN100 group, myelin recovery was much more substantial, associated with robust LFB staining and well-organized myelinated tracts. The neuroinflammatory markers reveal a significant reduction in vacuoles and macrophages, indicating the potency of high-dose PNN for promoting remyelination and controlling neuroinflammation. (Micro LFB Panel-G) in the EBRO + VB12 (30) group, which protects and repairs the myelin sheath. (Micro-LFB-Panel-H) In the EBRO + VB12 (30) +PNN100 combinations, with extensive remyelination and recovery of white matter integrity. The LFB staining intensity in this group is comparable to that of the control groups, and vacuolar size is markedly reduced. Macrophage infiltration is almost absent, indicating that neuroinflammation is efficiently controlled. This finding shows that the combined administration of VB12 (30) + PNN100 had a therapeutic effect, reducing demyelination and restoring myelin structural integrity in the cortex. PNN dosage, with higher doses being much more capable of repairing EBRO-induced white matter damage and restoring normal cortical functions. (Note: The black cube arrow symbolizes myelination/demyelination; the pink cube arrow shows macrophage infiltration; the orange cube arrow indicates vacuolization). (Magnification = ×40; Scale bar = 50 μm).

We used a fluorescent microscope with ×40 magnification to examine coronal slices of the cortex stained with Luxol Fast Blue (LFB). We performed this to assess myelination and white matter integrity. (Micro-LFB-Panel-A-B-C) Groups all had normal myelination and tract structure. This was evident in the healthy myelin sheaths, which did not change under the clear blue staining. These groups exhibited normal white matter integrity. (Micro-LFB-Panel-D) In the neurotoxin group, the EBRO group showed extensive demyelination, with loss of blue staining and increased vacuoles and macrophages, indicating an inflammatory response and tissue damage. (Micro-LFB-Panel-F) Treatment in the EBRO + PNN100 group resulted in marked remyelination, as evidenced by restoration of LFB blue staining, reduced vacuole formation, and decreased macrophage infiltration, indicating reduced neuroinflammation. (Micro-LFB-Panel-E) The EBRO + PNN50 group showed limited remyelination with partial myelin recovery. (Micro-LFB-Panel-G) EBRO + VB12 (30) led to patchy blue LFB reappearance, indicating neuroprotection and repair of myelin sheaths. (Micro-LFB-Panel-H) VB12 (30) + PNN100 substantially counteracted demyelination, achieving strong recovery of myelin and decreased vacuole formation and macrophage presence, indicating that it was particularly effective in normalising white matter integrity restoration (Figures 25A–H).

#### Hippocampus

**FIGURE 26:**
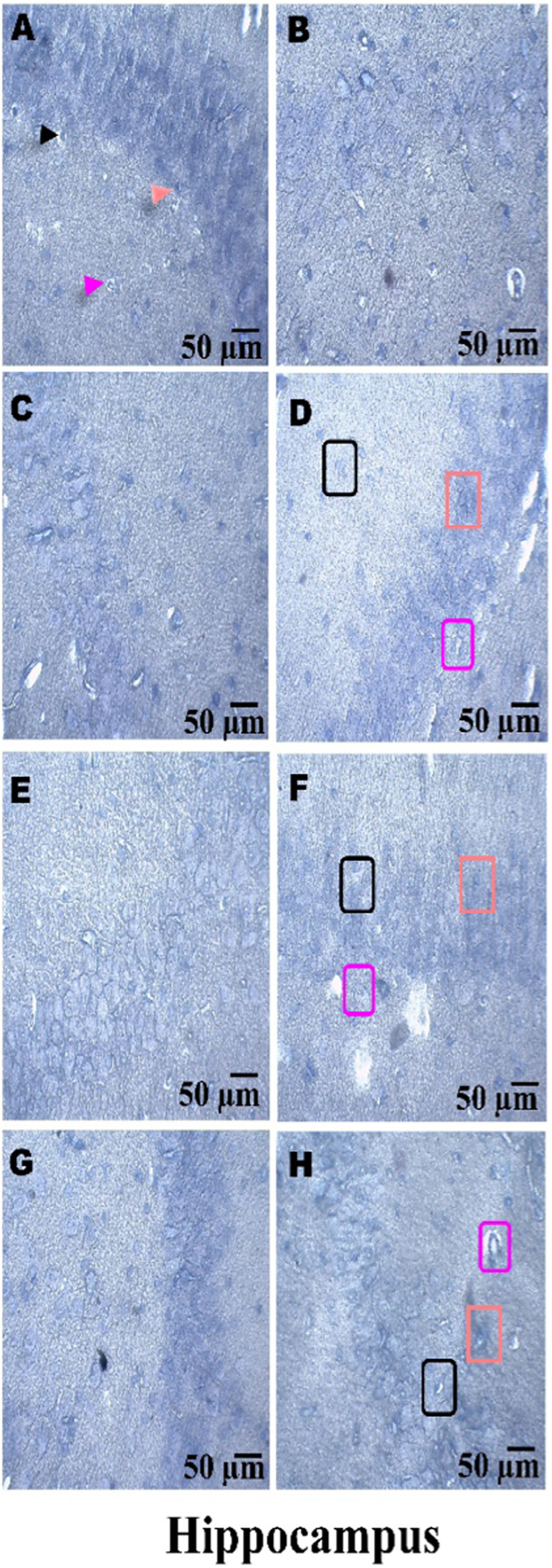
**(A–B)** Neuroprotection by PNN reduces LFB-stained histopathological changes in coronal section-hippocampus in rat model of MS. All the images presented in the Micro-LFB-Panel were made at a magnification of ×40 from the coronal sections of the hippocampus in adult Wistar rats treated with PNN stained. LFB staining is used exclusively to examine myelination; it stains white matter blue and has noted to show demyelination and remyelination. Experimental groups were as follows: **(A)** Sham Control, **(B)** Vehicle Control, **(C)** PNN Perse, **(D)** EBRO, **(E)** EBRO + PNN50, **(F)** EBRO + PNN100, **(G)** EBRO + VB12 (30), **(H)** EBRO + VB12 + PNN 100. (Micro-LFB-Panel-A-B-C) groups exhibited normal myelinated tracks depicted with the black trigon, and white matter tracts under the grey matter showed healthy myelin sheaths. Neurons were densely packed, showing minimal vacuole formation (shown by the orange trigon) as the entire white matter remained undamaged. (Micro-LFB-Panel D) The EBRO group exhibited massive demyelination, with the loss of blue staining in LFB indicating serious damage to the myelin sheaths, plus numerous heavily loaded macrophages as observed by the pink trigon; the products of myelin breakdown are associated with vacuole formation, denoting tissue degeneration while causing a spongy appearance in affected areas. (Micro-LFB-Panel-E) In the EBRO + PNN50 group, there is a moderate decrease in demyelination compared to the EBRO group. The restoration of the LFB staining indicates partial remyelination. (Micro-LFB-Panel-F) In the EBRO + PNN100 group, myelin recovery was much more robust with LFB staining and well-organised myelinated tracts. The neuroinflammatory markers show a significant reduction in vacuoles and macrophages, indicating that high-dose PNN exerts a potent effect on remyelination and neuroinflammatory control. (Micro-LFB-Panel-G) The EBRO + VB12 (30) group represents protection and repair of the myelin sheath. (Micro-LFB-Panel-H) In the EBRO + VB12 (30) + PNN100 combinations, extensive remyelination and recovery of white matter integrity. In this group, the LFB staining intensity is almost the same as that of the control groups, and vacuole size is markedly decreased. Macrophage infiltration is minimal, indicating that neuroinflammation is effectively controlled. This finding shows that the combined administration in the VB12 (30) + PNN100 group had a therapeutic effect, decreasing demyelination and restoring myelin structural integrity in the hippocampus. PNN100 dosage, with higher doses being much more capable of repairing EBRO-induced white matter damage and restoring normal hippocampal functions. (Note: The black cube symbolizes myelination/demyelination; the pink cube shows macrophage infiltration; the orange cube indicates vacuolization). (Magnification = ×40; Scale bar = 50 μm).

A micro-LFB panel of the hippocampus was observed under a fluorescent microscope at ×40 magnification to evaluate myelination and white matter integrity. Micro-LFB-Panel-A-B-C) Groups, the hippocampal sections showed normal myelination and tract structural development, with high blue staining intensity, indicating healthy myelin sheaths and no alterations. These groups served as a baseline for normal hippocampal white matter integrity. (Micro-LFB-Panel-D) In neurotoxin EBRO groups, significant demyelination was accompanied by obvious loss of blue staining, increased vacuole formation, and macrophage infiltration, indicative of an inflammatory response and substantial tissue damage in the hippocampal region. (Micro-LFB-Panel-E) In the EBRO + PNN50 group, limited remyelination is evidenced in the hippocampus by only partial myelin recovery through incomplete blue staining. (Micro-LFB-Panel-F) The EBRO + PNN100 group shows promoted remyelination, as evidenced by the re-establishment of blue staining in the hippocampus, reduced vacuole formation, and reduced macrophage infiltration, indicating a marked decline in neuroinflammation. (Micro-LFB-Panel-G) The EBRO + VB12 (30) group showed patchy blue LFB staining, suggesting neuroprotection with partial repair of myelin sheaths in the hippocampus. (Micro-LFB-Panel-H) In the EBRO + VB12 (30) + PNN100 group, there was a significant improvement in decreasing the demyelination, highly restoring myelin integrity along with decreased vacuole formation and reduction in macrophage presence, Figures 26A–H.

#### Striatum

**FIGURE 27:**
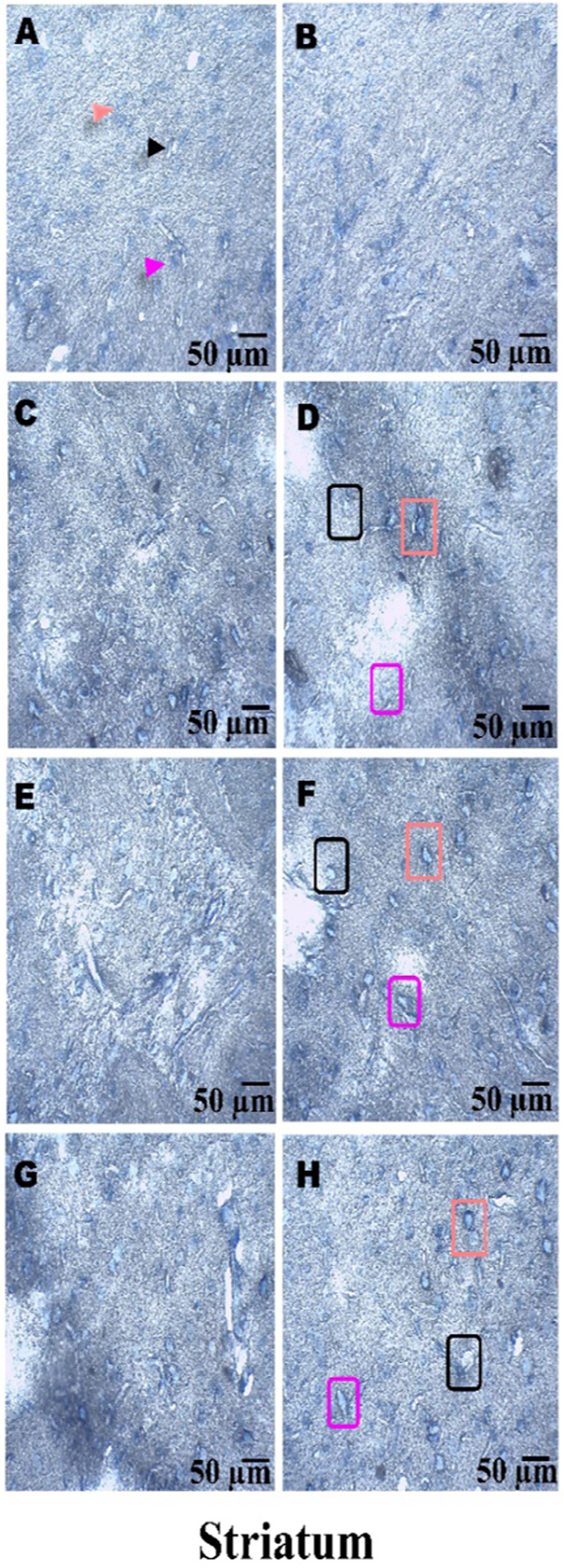
**(A–B)** Neuroprotection by PNN reduces LFB-stained histopathological changes in the coronal section of the striatum in the rat model of MS. All the images presented in the Micro-LFB-Panel were made at a magnification of ×40 from the coronal sections of the striatum in adult Wistar rats treated with PNN staining. LFB staining is used exclusively for the examination of myelination; it stains white matter blue and notes demyelination and remyelination. The experimental groups included **(A)** Sham control, **(B)** Vehicle control, **(C)** PNN Perse, **(D)** EBRO, **(E)** EBRO + PNN50, **(F)** EBRO + PNN100, **(G)** EBRO + VB12 (30), and **(H)** EBRO + VB12 (30) + PNN100. (Micro-LFB-Panel-A-B-C) groups exhibited normal myelinated tracks, followed by the black trigon, and the myelinated fibers right under the grey matter showed healthy myelin sheaths. Neurons clustered together finely, showing very few vacuoles formed (shown by the orange trigon), as the remaining white matter. (Micro-LFB-Panel-D) In the neurotoxin EBRO, more demyelination and the complete loss of blue staining in LFB were observed, revealing severe damage to the myelin sheaths and macrophages seen as the pink trigon. Myelin breakdown products are associated with the formation of vacuoles, signifying tissue degeneration and creating a spongy appearance in affected regions. (Micro-LFB-Panel-E) The EBRO + PNN50 group showed significantly less demyelination than the EBRO group, with partial restoration of LFB staining indicating remyelination. (Micro-LFB-Panel-F) In the EBRO + PNN100 group, myelin recovery was much higher, with robust LFB staining and finely organized myelinated tracks. The neuroinflammatory markers showed a significant reduction in vacuoles and macrophages, indicating that high-dose PNN packs support remyelination and control neuroinflammation. (Micro-LFB-Panel-G) The EBRO + VB12 (30) group indicates that protection and repair of the myelin sheath occur. This group remyelinated and recovered white matter structural integrity; LFB staining intensity was approximately equal to that of controls, with a marked reduction in vacuole size. Furthermore, macrophage infiltration is nearly absent, indicating that neuroinflammation is controlled. (Micro-LFB-Panel-H) VB12 (30) + PNN100 group therapeutic effect through moderating demyelination and regaining the structural integrity of myelin in the striatum. Higher doses of PNN100 are much better than the lower ones in repairing EBRO-induced white matter damage and returning striatal functions to normal. (Note: The black cube symbolizes myelination/demyelination; the pink cube shows macrophage infiltration; the orange cube indicates vacuolization). (Magnification = ×40; Scale bar = 50 μm).

The micro-LFB panel of the striatum was assessed under a fluorescent microscope at 40X for myelination and integrity of white matter. (Micro-LFB-Panel-A-B-C) The striatum sections showed normal myelination and the branching structures of tracts with a high blue staining intensity of healthy myelin sheaths and no alterations. These groups provided a normal striatal white matter integrity. (Micro-LFB-Panel-D) In the neurotoxin EBRO groups, substantial demyelination was also evident, as indicated by pronounced loss of blue staining and vacuole formation, with some macrophage infiltration, reflecting the inflammatory response and actual tissue damage in the striatum. (Micro-LFB-Panel-E) The EBRO + PNN 50 exhibited patches of remyelination in the striatum, indicated by incomplete recovery of blue myelin staining. (Micro-LFB-Panel-F) In the EBRO + PNN100 group, promoted remyelination re-established blue staining in the striatum, with reduced vacuole formation and fewer macrophages, indicating a notable reduction in neuroinflammation. (Micro-LFB-Panel-G) The EBRO + VB12 (30) group showed patchy blue staining for the LFB, indicating neuroprotection and repair of myelin sheaths in the striatum. (Micro-LFB-Panel-H) The EBRO + VB12 (30) + PNN100 group showed greater demyelination mitigated, with evidence of blue staining restoring myelin integrity, accompanied by reduced vacuole formation and a remarkable reduction in macrophage presence (Figures 27A–H).

#### Midbrain

**FIGURE 28:**
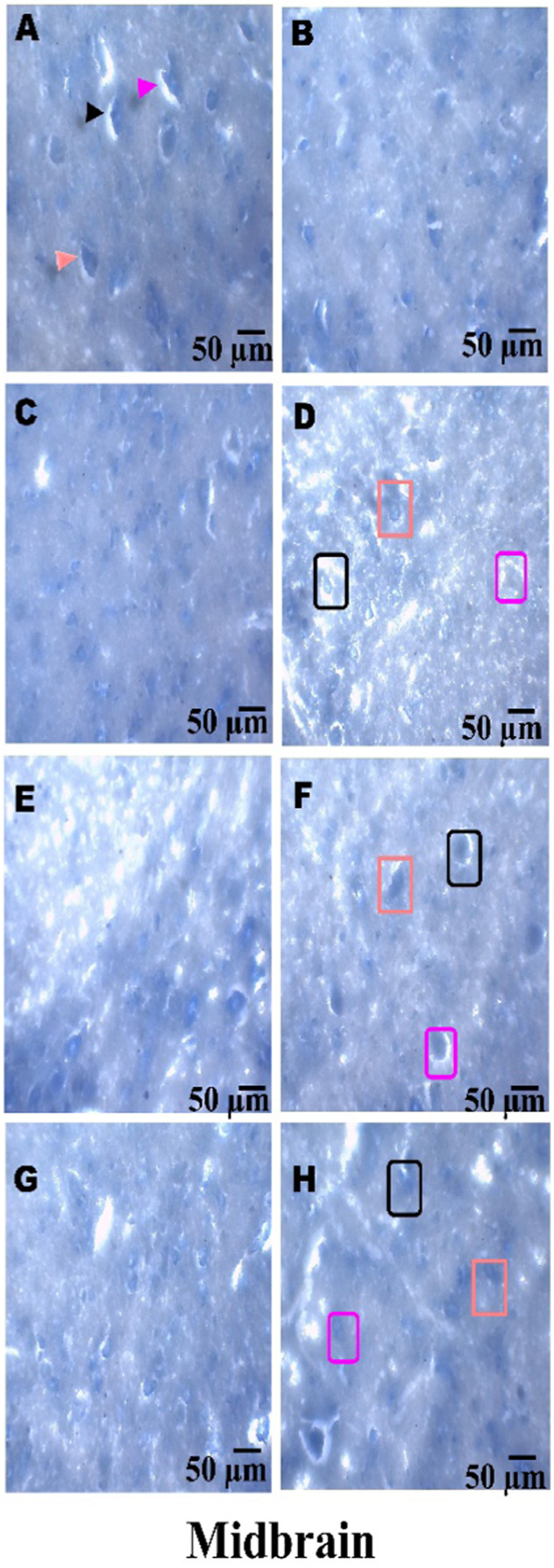
**(A–H)** Neuroprotection by PNN reduces LFB-stained histopathological changes in coronal section-midbrain in rat model of MS. All the images that are represented in the Micro-LFB-Panel were made at a magnification of ×40 from the coronal sections of the adult Wistar rat midbrain with PNN staining. LFB staining is for the examination of only myelination; it stains white matter blue while advising on demyelination and remyelination. The experimental groups were: **(A)** Sham Control, **(B)** Vehicle Control, **(C)** PNN Perse, **(D)** EBRO, **(E)** EBRO + PNN50, **(F)** EBRO + PNN100, **(G)** EBRO + VB12 (30), **(H)** EBRO + VB12 + PNN100. (Micro-LFB-Panel-A-B-C) groups showed normal myelinated tracks depicted with the black trigon, and myelin sheaths under diseased grey matter were normal. The neurons were packed so tightly, with less cellular vacuolation (orange trigon), that the entire white matter remained intact. (Micro-LFB-Panel D) In the EBRO group, extensive demyelination was observed, alongside the absence of blue staining in LFB; this was highly indicative of myelin sheath damage, shown by pink trigons showing numerous heavily-loaded macrophages; breakdown of myelin was found clustered and causing vacuolation, indicating degeneration of affected tissue and a spongy appearance. (Micro-LFB-Panel-E) The EBRO + PNN50 group showed a moderate decrease in demyelination compared with the EBRO group. (Micro-LFB-Panel-F) In the EBRO + PNN100 group, myelin recovery was seen as extremely high LFB staining intensity and well-organised myelinated tracts. As indicated by neuroinflammatory markers, fewer vacuoles and macrophages were observed, suggesting that high-dose PNN has a potent effect on remyelination and on inhibiting neuroinflammation. (Micro-LFB-Panel-G) The EBRO + VB12 (30) group signifies repair and protection of the myelin sheath. (Micro-LFB-Panel-H) The EBRO + VB12 (30) + PNN100 combination denotes widespread remyelination and repair of the integrity of white matter. In this group, LFB staining intensity approaches that of the control groups, with markedly reduced vacuolar sizes. Macrophage infiltration is a clear indication that neuroinflammation is effectively contained. VB12 (30) + PNN100 has a great therapeutic potential for reducing demyelination and restoring the integrative structure of the midbrain. (Note: The black cube symbolizes myelination/demyelination; the pink cube shows macrophage infiltration; the orange cube indicates vacuolization). (Magnification = ×40; Scale bar = 50 μm).

Fluorescent microscopy at ×40 magnification was performed on the micro-LFB panel midbrain to assess myelination and white matter integrity. Micro-LFB-Panel-A-B-C) Groups showed myelination and tract structural development in midbrain slices that appeared normal, with high blue staining intensity indicating healthy myelin sheaths without alterations. These groups had normal midbrain integrity. (Micro-LFB-Panel-D) The neurotoxin EBRO groups exhibited severe demyelination, with pronounced loss of blue staining, increased vacuolar formation, and macrophage infiltration, indicating a pronounced tissue-destructive response in the midbrain. (Micro-LFB-panel-E) The EBRO + PNN 50 group is represented in the midbrain with only partial restoration of myelin through incomplete blue staining. (Micro-LFB-panel-F) In EBRO + PNN100, the treatment promoted remyelination, as evidenced by the re-establishment of blue staining in the midbrain, with less extensive vacuolar formation and macrophage infiltration, indicating a rapid decrease in neuroinflammation. (Micro-LFB-Panel-G) This EBRO + VB12 (30) group exhibits patchy blue staining for LFB, which suggests neuroprotection and partial repair of the myelin sheaths in the midbrain. (Micro-LFB-Panel-H) In the EBRO + VB12 (30) + PNN100, a marked change in the restoration of demyelination is shown, restoring myelin integrity enhanced with even less vacuole formation and reduction in macrophage presence, Figures 28A–H.

#### Cerebellum

**FIGURE 29:**
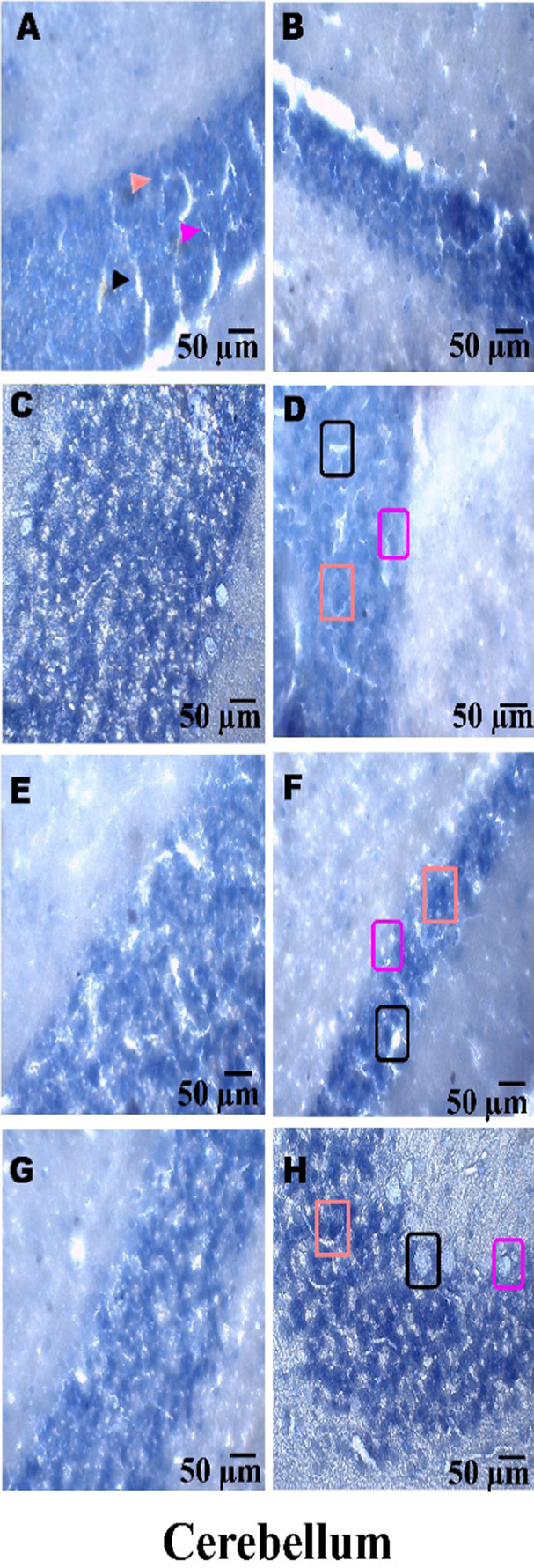
**(A–H)** Neuroprotection by paeoniflorin reduces LFB-stained histopathological changes in coronal section-cerebellum in rat model of multiple sclerosis. All images presented in the Micro-LFB-Panel were captured at a magnification of ×40, using coronal sections from the cerebellum of adult Wistar rats. This LFB staining technique is primarily used to examine myelination, staining white matter blue and highlighting demyelination and remyelination. The experimental groups were categorized as follows: **(A)** Sham Control, **(B)** Vehicle Control, **(C)** PNN Perse, **(D)** EBRO, **(E)** EBRO + PNN50, **(F)** EBRO + PNN100, **(G)** EBRO + VB12 (30), and **(H)** EBRO + VB12 (30) + PNN100. (Micro-LFB-Panel-A-B-C) group exhibited normal myelinated tracks as indicated by black trigons. Additionally, the underlying white and grey matter showed intact myelin sheaths. Neurons appeared densely packed with minimal vacuole formation, as marked by orange trigons; overall, the white matter remained undamaged. (Micro-LFB-Panel-D) group, significant demyelination was observed in the EBRO group. The loss of blue coloration in LFB highlighted severe damage to myelin sheaths and numerous macrophages in affected regions—this association was shown by pink trigons. (Micro-LFB-Panel-E) The EBRO + PNN50 group showed a smaller decrease in demyelination than the EBRO group. (Micro-LFB-Panel-F) The EBRO + PNN100 group closely reveals a more robust recovery characterized by intense LFB staining and well-organized myelinated tracts. Neuroinflammatory markers showed a notable decrease in both vacuoles and macrophages, and a high dose of PNN promoted remyelination while effectively managing neuroinflammation. (Micro-LFB-Panel-G) The EBRO + VB12 (30) group exhibits protection and repair of the myelin sheath. (Micro-LFB-Panel-H) The EBRO + VB12 (30) + PNN100 combination shows extensive remyelination and recovery of white matter integrity. In this group, the LFB staining density is comparable to that of the control groups with reduced vacuolar size. Macrophage infiltration appears to be absent. This result shows that the combination of VB12 (30) + PNN100 has a strong therapeutic effect in decreasing demyelination and restoring myelin structural integrity in the cerebellum. PNN dosage should be more effective in restoring EBRO-induced white matter damage and restoring cerebellar function to normal levels. (Note: The black cube arrow symbolizes myelination/demyelination; the pink cube arrow shows macrophage infiltration; the orange cube arrow indicates vacuolization) (Magnification = ×40; Scale bar = 50 μm).

The Micro-LFB-Panel of the cerebellum was under a fluorescent microscope at ×40 magnification to evaluate myelination and the integrity of white matter. (Micro-LFB-Panel-A-B-C) Group displays have normal myelination and tract structural development, with high blue staining intensity indicating healthy myelin sheaths and no alterations. (Micro-LFB-Panel-D) In the EBRO neurotoxin groups, substantial demyelination was evident, with pronounced loss of blue staining, increased vacuolar formation, and macrophage infiltration, indicating an inflammatory response and extensive tissue damage in the cerebellum. (Micro-LFB-Panel-E) The EBRO + PNN50 group showed only partial myelin recovery, as evidenced by incomplete blue staining. (Micro-LFB-Panel-F) In the EBRO + PNN100 group, remyelination was promoted, as seen by resetting blue staining in the cerebellum, which had decreased vacuole formation and decreased macrophage infiltration, emphasizing an appropriate reduction in neuroinflammation. (Micro-LFB-Panel-G) The EBRO + VB12 (30) group exhibited patchy blue staining for LFB, suggesting neuroprotection with a partial repair of myelin sheaths in the cerebellum. (Micro-LFB-Panel-H) In the EBRO + VB12 (30) + PNN100 group, significant improvement in moderating the demyelination was prominent, with myelin integrity restored, a further significant reduction in vacuole formation, as well as diminished macrophage presence in the cerebellum, Figures 29A–H.

## Discussion

The current study aimed to assess PNN neuroprotective effects in a rat model of EBRO-induced neurodegeneration that replicates the pathological characteristics of MS. Clinically recognized as a chronic neuroinflammatory condition, MS primarily targets the myelin sheath within the central nervous system, leading to progressive neurological dysfunction (Steinman et al., 2022; Bar-or et al., 2023; Harris et al., 2024).

Our study, supported by complementary *in silico* analyses, utilized a broad spectrum of biological samples, including CSF, blood plasma, and key brain regions such as the CC, HC, STR, MB, and CBL. The cognitive and motor processes of learning, spatial memory, neuromuscular coordination, gait abnormality, and locomotor activity, all of which are commonly compromised in MS, are significantly impacted by these brain regions. The identification of potential biomarkers important to MS pathophysiology was facilitated by CSF and blood plasma analyses, which also provided valuable insights into systemic biochemical changes. These included neurotransmitters such as serotonin, glutamate, GABA, acetylcholine, and dopamine, as well as anti-inflammatory cytokines, essential molecular targets, and indicators of apoptosis and neuronal death.

Additionally, hematological parameters were examined to evaluate more extensive systemic changes. To comprehensively investigate the neurochemical, cellular, and systemic disturbances induced by EBRO exposure, our study employed a multifaceted approach integrating histopathological, molecular, and gross morphological analyses. Techniques such as, H&E staining, LFB staining, and whole-brain morphological assessments were utilized to capture the extent of neuronal injury, demyelination, and inflammatory responses. Notably, this study is among the few that concurrently explore these diverse pathological layers within a single experimental MS model.

PNN administration significantly ameliorated EBRO-induced neurodegeneration, as evidenced by restored myelin integrity, preserved neuronal architecture, and modulated expression of key signaling proteins. These findings underscore the novelty of this work in demonstrating the multi-level neuroprotective potential of PNN and its translational relevance in mitigating MS-associated neuropathology. These findings align with previous preclinical studies demonstrating the neuroprotective effects of PNN in animal models of AD (Kong et al., 2020), PD (He et al., 2022), and spinocerebellar ataxia (Kong et al., 2020).

Additionally, our findings provide the first evidence that PNN influences the GDNF/GFRA1/RET/AKT/ERK1/2/GSK3β signaling pathway in an experimental model of MS, shedding light on its underlying mechanisms and therapeutic relevance. Our findings highlight the neuroprotective potential of PNN and VB12 (30) in alleviating demyelination, neuroinflammation, and synaptic dysfunction in MS. The co-administration of PNN and VB12 (30) demonstrated a synergistic therapeutic effect, providing greater efficacy than either agent alone. This study further highlights their complementary roles in restoring neurochemical balance and improving behavioral outcomes disrupted by EBRO-induced MS pathology. The dual treatment effectively mitigated the excitatory-inhibitory imbalance, reduced inflammation, and enhanced motor and cognitive performance, underscoring the potential of this combinatorial approach to address the multifaceted nature of MS.

According to molecular docking studies, PNN exhibits strong binding to a number of important proteins involved in neuroprotective signaling, such as GDNF, GFRA1, RET, AKT, ERK1/2, and GSK3β. These interactions suggest that PNN may modulate these targets to initiate beneficial pathways associated with inflammation regulation, remyelination, and neuronal survival. PNN to regulate excessive or dysfunctional signaling, which is commonly observed in MS, is supported by the favorable binding energies. Computational analyses highlight the promise of PNN as a multi-target therapeutic agent, consistent with experimental findings. Nonetheless, comprehensive investigations, including pathway-specific assessments and receptor-binding studies, are essential to elucidate its precise mechanism of action and establish its therapeutic relevance in MS.

Clinical and preclinical studies have consistently demonstrated that MS is associated with profound neurobehavioral impairments, including deficits in motor coordination, reduced locomotor activity, and impaired spatial memory. These functional abnormalities reflect underlying demyelination, synaptic dysfunction, and neurodegeneration, and are commonly observed in both patients and validated experimental models of MS (Mofateh et al., 2022; Abreu-Corrales et al., 2023; Podda et al., 2024; Essawy et al., 2025; Santos, 2025).

In this study, EBRO-exposed animals exhibited a marked decline in motor performance and spatial memory, reflecting the functional impairments characteristic of MS. Rotarod performance confirmed deficits in motor coordination and balance. At the same time, results from the beam crossing test further highlighted impairments in gait stability and postural control. Subsequent actophotometer assessments revealed a significant reduction in locomotor activity, and Morris water maze performance indicated spatial memory deficits, as evidenced by increased escape latency and decreased time spent in the target quadrant. Treatment with PNN100 combined with VB12 effectively restored these functional impairments, demonstrating their therapeutic potential in improving muscle coordination, locomotor activity, gait abnormalities, spatial memory, and cognitive outcomes in MS. Clinical and preclinical studies have shown that MS is linked to significant decreases in body weight and brain-to-body weight ratio, reflecting underlying neurodegenerative processes (Cozart et al., 2023; Niiranen et al., 2022). In our study, EBRO administration resulted in significant decreases in body weight and the brain-to-body weight ratio, indicative of neurodegenerative changes. In our study, treatment with PNN100 and VB12 (30) successfully restored both body weight and the brain-to-body weight ratio toward normal levels.

Multiple clinical and preclinical studies have shown that in MS, neurotrophic factors like GDNF and its co-receptor GFRA1 are downregulated, while key intracellular signaling molecules like ERK1/2, AKT, and GSK3-β are hyperactivated (Galimberti et al., 2011; Gao et al., 2016; Attia et al., 2023; Sun R. et al., 2024). Our study found that EBRO exposure significantly increased ERK1/2, AKT, and GSK3-β expression, whereas it decreased GDNF and GFRA1 levels in brain homogenates and CSF. In our study, therapeutic administration of PNN100 and VB12 (30) effectively ameliorated these molecular abnormalities by restoring the expression of GDNF and GFRA1, as well as normalizing the activities of AKT, ERK1/2, and GSK3-β to baseline levels. ELISA quantification further corroborated the alterations in neurotrophic and excitotoxic markers. Collectively, these findings demonstrate that PNN100 and VB12 (30) confer neuroprotection in MS by modulating neurotrophic and excitotoxic pathways, preserving synaptic integrity, and attenuating the progression of neuropathological alterations.

Activation of GDNF signaling through GFRA1 and RET leads to downstream upregulation of AKT and ERK1/2 pathways, promoting neuronal survival, anti-apoptotic signaling, oligodendrocyte protection, and remyelination (Li et al., 2022; Suzuki et al., 2025). GDNF signaling promotes dopaminergic and glutamatergic homeostasis, which could explain the restoration of neurotransmitters and the subsequent relief of behavioral parameters (Uzay and Zhang, 2025). Directly coupled molecular neuroprotection and decreased inflammatory load with functional recovery in motor and cognitive behavioral studies and provided them as downstream effects of pathway alteration (Xiaoyu et al., 2024). EBRO toxicity: impairs GDNF/GFRA1/RET signaling; suppresses AKT/ERK stimulation; high GSK3B activity; apoptosis, inflammation, demyelination, neurotransmitter imbalance; behavioral deficits. Restoration of this axis following treatment resulted in partial structural and functional restoration (Leem et al., 2023).

MS is characterized by two key pathological processes: oxidative stress and chronic neuroinflammation, which collectively activate the intrinsic apoptotic pathway. Clinical and preclinical studies consistently report upregulation of pro-apoptotic markers, including caspase-3 and Bax, alongside downregulation of the anti-apoptotic protein Bcl-2, contributing to progressive neuronal loss and disease progression (Seidi and Sharief, 2002; Cid et al., 2003; Ruggieri et al., 2006; Ferreira, 2014). Our study demonstrates that co-administration of PNN100 and VB12 (30) effectively decrease EBRO-induced changes in apoptotic markers in brain homogenates, highlighting their therapeutic potential to preserve neuronal viability and mitigate neurodegeneration in multiple sclerosis.

Clinically, MS is characterized by progressive neurodegeneration, including extensive demyelination, neuronal loss, gliosis, and axonal damage, as consistently reported in neuropathological examinations of MS patient brain tissue (Haider et al., 2016). These pathological features are particularly prominent in regions such as the corpus callosum, hippocampus, cerebellum, and cerebral cortex, thereby contributing directly to the motor, cognitive, and sensory deficits observed in patients with MS (Geurts et al., 2007; Klaver et al., 2013). Preclinical MS models, including immune- and toxin-based paradigms, have effectively recapitulated these neuropathologies. Histological assessments using H&E staining in these models typically reveal widespread neuronal degeneration, vacuolation, and reactive gliosis, while LFB staining demonstrates clear myelin loss (Meyer et al., 2001; Geurts et al., 2007; Norkute et al., 2009; Horstmann et al., 2013; Joost et al., 2022). In our study EBRO-induced MS model exhibited severe histopathological damage, as evidenced by prominent neuronal loss, glial activation, and demyelination across multiple brain regions. Importantly, co-treatment with PNN100 and VB12 (30) preserved neural structure, reduced gliosis, and restored myelin integrity, as shown by improved H&E and LFB staining patterns. These results highlight the neuroprotective and remyelinating efficacy of the combined therapy and reinforce its translational potential in mitigating MS-related neuropathology.

Gross morphological and histopathological alterations serve as key indicators of neuropathology in multiple sclerosis, reflecting extensive demyelination and progressive neurodegeneration. Both clinical observations and preclinical models consistently demonstrate these structural changes, underscoring their relevance in disease progression and therapeutic evaluation. These structural abnormalities are often accompanied by neurobehavioral deficits and imbalances in neurotransmitter systems (Kukanja et al., 2024; Muñoz González et al., 2025). In our experimental model, EBRO exposure resulted in marked regional brain atrophy, decreased brain weight and tissue volume, and reduced neuronal and glial cell densities in key regions, including the cerebral cortex, hippocampus, striatum, midbrain, and cerebellum, findings that align with the characteristic neuropathology of multiple sclerosis. Importantly, treatment with PNN100 and VB12 (30) effectively reduced these pathological alterations, restoring brain morphology, cellular integrity, and neurotransmitter balance, thereby highlighting their therapeutic potential in mitigating MS-associated neurodegeneration.

Furthermore, ELISA analysis of brain homogenates revealed that PNN significantly modulated neurotransmitter levels, highlighting its role in restoring neurochemical balance and mitigating the structural and functional deficits associated with MS. Neurotransmitter levels were measured to determine functional neuronal integrity. The GDNF/GFRA1/RET pathway, which typically maintains dopaminergic and synaptic integrity, is not as well activated when EBRO-mediated inflammatory stress interferes with neurotrophic transmission. Increased GSK3-Beta activity leads to synaptic dysfunction and neurotransmitter imbalance, whereas decreased downstream signaling by AKT and ERK1/2 encourages neuronal stress and apoptosis. By reactivating GDNF-dependent signaling, increasing AKT/ERK1/2 survival pathways, and reducing GSK3-Beta overactivation. Acetylcholine, dopamine, serotonin, glutamate, and GABA are essential for regulating diverse central nervous system functions. In multiple sclerosis, persistent demyelination, chronic neuroinflammation, and synaptic dysfunction lead to profound disruptions in these neurotransmitter systems, as consistently demonstrated across both clinical and preclinical studies (Reale et al., 2012; Di Bari et al., 2016; Arm et al., 2021; Melnikov et al., 2022). The cerebral cortex primarily relies on glutamatergic, GABAergic, and cholinergic signaling for the integration of sensory information and cognitive processing, while the hippocampus utilizes glutamate, GABA, serotonin, and acetylcholine to support learning and memory functions (Yousefi et al., 2012). The striatum and midbrain regulate motor activity through dopaminergic and GABAergic signaling, while the cerebellum integrates GABAergic, glutamatergic, and serotonergic inputs to coordinate both motor and cognitive functions (Tritsch et al., 2014). Findings from this EBRO-induced MS model align with both clinical and preclinical data, demonstrating an imbalance between excitatory and inhibitory neurotransmission, characterized by elevated glutamate levels alongside reduced concentrations of GABA, dopamine, serotonin, and acetylcholine. Treatment with PNN100 and VB12 (30) effectively restored these altered neurochemical levels toward normal levels, suggesting their therapeutic potential in re-establishing synaptic balance and improving neurofunctional outcomes in MS.

MS is characterized by elevated levels of pro-inflammatory cytokines, including TNF-α and IL-1β, alongside reduced expression of anti-inflammatory mediators such as IL-10, reflecting a sustained imbalance in immune regulation as demonstrated in both clinical and preclinical studies (Farsani et al., 2015; Blandford et al., 2021; Kalvaitis et al., 2024). In our EBRO-induced MS model, we observed significant increases in TNF-α and IL-1β, with a concurrent reduction in IL-10 in both brain homogenates and plasma, confirming a robust pro-inflammatory response. In our studies, PNN100 and VB12 (30) effectively normalized these cytokine levels, highlighting their potential to modulate neuroinflammation in MS. Clinical and preclinical investigations have identified reduced MBP and elevated NEFL levels as critical biomarkers of demyelination and axonal damage in MS. In our study, treatment with PNN100 and VB12 (30) effectively restored these biomarkers to normal levels, highlighting their therapeutic potential in preserving CNS structural integrity.

Clinical and preclinical studies in MS have consistently revealed substantial hematological abnormalities, including increased eosinophil and basophil counts, coupled with decreased levels of neutrophils, monocytes, lymphocytes, RBC, hemoglobin, WBC, and platelets (Johnson et al., 2022; Sambasiva and Bairaboina, 2023). These hematologic disruptions reflect underlying peripheral inflammation, anemia, and systemic immunosuppression, which are often correlated with disease severity and progression (Demitto et al., 2020). In our study, the EBRO-induced MS model exhibited pronounced deviations in haematological parameters, characterised by elevated eosinophils and basophils, and reduced neutrophils, lymphocytes, monocytes, RBCs, haemoglobin, and platelet levels, suggesting systemic immune dysfunction, anaemia, and potential bone marrow suppression. Notably, treatment with PNN100 and VB12 (30) significantly restored these altered hematological indices, normalizing leukocyte subsets, promoting erythropoiesis, and improving thrombopoiesis. These findings highlight the therapeutic potential of this combinatorial strategy in correcting peripheral hematological abnormalities associated with MS.

This study offers a new approach to MS research by combining diagnostic and treatment strategies that analyze markers from different biological sources: brain tissue, CSF, and blood plasma. By examining changes in both the central and peripheral nervous systems, we identified key disease processes, including excitotoxicity, impaired synaptic function, inflammation, neuronal damage, and disruptions in chemical and tissue balance. These results highlight the importance of using biomarkers from multiple sources to track disease progression and treatment effects. Notably, treatment with PNN100 and VB12 (30) ameliorated these disease-related changes and confirmed the utility of these biomarkers for monitoring therapy. To our knowledge, this is the first preclinical study to demonstrate that PNN alters disease outcomes and supports the use of biomarkers in MS, providing a foundation for more precise diagnosis and targeted treatment development.

## Conclusion

The study underscores the significant neuroprotective potential of PNN in mitigating the complex pathology of MS through its multifaceted therapeutic effects. Using a well-validated EBRO rat model, the research highlights PNN ability to address key pathological hallmarks of MS, including demyelination, neuroinflammation, synaptic dysfunction, and systemic immune dysregulation. By employing a combination of *in silico*, *in-vitro*, and *in-vivo* analyses, the study demonstrates the effectiveness of PNN in modulating critical molecular pathways, restoring physiological and behavioral deficits, and moderating neurodegenerative changes. The findings not only validate PNN as a standalone therapy but also emphasize its enhanced efficacy when co-administered with VB12 (30), showcasing the synergistic benefits of combinatorial treatment strategies. PNN showed dose-dependent efficacy in reducing neurodegenerative outcomes caused by EBRO, with the higher dose (100 mg/kg) producing pronounced improvements in behavioral, biochemical, molecular, and histopathological parameters. When combined with VB12 (30), the therapeutic outcomes were further enhanced, as evidenced by superior recovery in motor coordination, spatial memory, neurotransmitter balance, and inflammatory markers. The combination therapy also demonstrated robust remyelination and attenuation of neuroinflammation, as evidenced by LFB staining of brain sections and reduced inflammatory cell infiltration. These outcomes suggest that PNN, particularly when combined with VB-12, effectively restores neural integrity and functional performance in MS. At the molecular level, PNN modulates the GDNF/GFRA1/RET/AKT/ERK1/2/GSK3β signalling pathway, which plays a pivotal role in neuroprotection, remyelination, and synaptic plasticity. EBRO-induced downregulation of neurotrophic factors GDNF and GFRA1 and upregulation of pro-apoptotic and inflammatory markers GSK3-Beta, AKT, and ERK1/2 were significantly reduced by PNN treatment, as validated through ELISA. Additionally, PNN normalized neurotransmitter imbalances, including elevated glutamate and reduced levels of acetylcholine, dopamine, serotonin, and GABA, thereby mitigating excitotoxicity and synaptic dysfunction. These results highlight PNN potential to restore homeostasis in the central nervous system by targeting multiple molecular pathways and neurochemical processes. Histopathological analyses further validated the neuroprotective effects of PNN. Brain regions frequently affected in MS, such as the cerebral cortex, hippocampus, striatum, midbrain, and cerebellum, showed significant structural recovery following PNN treatment. Observations included reduced vacuole formation, improved myelin integrity, and decreased gliosis, indicating robust anti-inflammatory and anti-demyelinating properties. Combining PNN100 with VB12 (30) yielded the most striking results, with near-complete restoration of normal brain morphology and cellular architecture. These findings were complemented by gross morphological analyses, which revealed recovery of overall brain size and weight, further underscoring the therapeutic potential of PNN in decreasing EBRO-induced neurodegeneration. PNN also demonstrated systemic benefits by normalizing hematological parameters disrupted by EBRO, including leukocyte, erythrocyte, and platelet counts, thereby addressing systemic immune dysfunction. Moreover, the study provided a comprehensive biomarker-based approach by analyzing markers such as MBP and NEFL in brain tissue, CSF, and blood plasma. The normalization of these biomarkers following PNN treatment offers a valuable translational framework for monitoring disease progression and therapeutic efficacy in MS. This research is among the first to demonstrate the therapeutic relevance of PNN in modulating the GDNF/GFRA1/RET/AKT/ERK1/2/GSK3-Beta pathway in the context of MS. The integration of *in silico* and experimental methodologies provides a robust understanding of PNN mechanisms of action, highlighting its potential as a multi-target therapeutic agent. Furthermore, the synergistic effects of PNN100 and VB12 (30) underscore the importance of exploring combinatorial treatment strategies to address the multifactorial nature of MS. Future studies are warranted to evaluate the long-term safety and efficacy of PNN, optimize dosing regimens, and explore its applicability in other neurodegenerative and neuroinflammatory conditions. Collectively, this study establishes PNN as a promising candidate for MS therapy, offering hope for improved clinical outcomes and quality of life for patients suffering from this debilitating disease.

## Data Availability

The raw data supporting the conclusions of this article will be made available by the authors, without undue reservation.

## Glossary

AD: Alzheimer’s disease
AKT: Protein Kinase B
ALS: Amyotrophic lateral sclerosis
BBB: Blood-brain barrier
CBC: Complete Blood Count
CNS: Central nervous system
CSF: Cerebrospinal Fluid
EBRO: Ethidium Bromide
EDTA: Ethylenediaminetetraacetic Acid
ERK1/2: Extracellular signal-Regulated Kinases 1 and 2
EST: Escape latency time
GDNF: Glial cell line-Derived Neurotrophic Factor
GFRA1: GDNF Family Receptor Alpha 1
GSK3-β: Glycogen Synthase Kinase 3 Beta
H&E: Hematoxylin and Eosin
HD: Huntington’s disease
IAEC: Institutional Animal Ethics Committee
IL-10: Interleukin-10
IL-1β: Interleukin-1-beta
LFB: Luxol Fast Blue
MBP: Myelin Basic Protein
MS: Multiple sclerosis
MWM: Morris Water Maze
NEFL: Neurofilament Light Chain
PD: Parkinson’s disease
PNN: Paeoniflorin
RET: Rearranged during Transfection
TNF-Alpha: Tumor Necrosis Factor Alpha
TNF-α: Tumor Necrosis Factor alpha
TSTQ: Target Quadrant time spent
VB-12: Vitamin B12
